# Data processing solutions to render metabolomics more quantitative: case studies in food and clinical metabolomics using Metabox 2.0

**DOI:** 10.1093/gigascience/giae005

**Published:** 2024-03-15

**Authors:** Kwanjeera Wanichthanarak, Ammarin In-on, Sili Fan, Oliver Fiehn, Arporn Wangwiwatsin, Sakda Khoomrung

**Affiliations:** Siriraj Center of Research Excellence in Metabolomics and Systems Biology (SiCORE-MSB), Faculty of Medicine Siriraj Hospital, Mahidol University, Bangkok 10700, Thailand; Siriraj Metabolomics and Phenomics Center, Faculty of Medicine Siriraj Hospital, Mahidol University, Bangkok 10700, Thailand; Siriraj Center of Research Excellence in Metabolomics and Systems Biology (SiCORE-MSB), Faculty of Medicine Siriraj Hospital, Mahidol University, Bangkok 10700, Thailand; Siriraj Metabolomics and Phenomics Center, Faculty of Medicine Siriraj Hospital, Mahidol University, Bangkok 10700, Thailand; Department of Biostatistics, University of California Davis, Davis, CA 95616, USA; West Coast Metabolomics Center, University of California Davis Genome Center, Davis, CA 95616, USA; Department of Systems Biosciences and Computational Medicine, Faculty of Medicine, Khon Kaen University, Khon Kaen 40002, Thailand; Siriraj Center of Research Excellence in Metabolomics and Systems Biology (SiCORE-MSB), Faculty of Medicine Siriraj Hospital, Mahidol University, Bangkok 10700, Thailand; Siriraj Metabolomics and Phenomics Center, Faculty of Medicine Siriraj Hospital, Mahidol University, Bangkok 10700, Thailand; Department of Biochemistry, Faculty of Medicine Siriraj Hospital, Mahidol University, Bangkok 10700, Thailand; Center of Excellence for Innovation in Chemistry (PERCH-CIC), Faculty of Science, Mahidol University, Bangkok 10700, Thailand

**Keywords:** metabolomics, quantitative analysis, semiquantitative analysis, data processing, normalization, transformation, scaling, R package

## Abstract

In classic semiquantitative metabolomics, metabolite intensities are affected by biological factors and other unwanted variations. A systematic evaluation of the data processing methods is crucial to identify adequate processing procedures for a given experimental setup. Current comparative studies are mostly focused on peak area data but not on absolute concentrations. In this study, we evaluated data processing methods to produce outputs that were most similar to the corresponding absolute quantified data. We examined the data distribution characteristics, fold difference patterns between 2 metabolites, and sample variance. We used 2 metabolomic datasets from a retail milk study and a lupus nephritis cohort as test cases. When studying the impact of data normalization, transformation, scaling, and combinations of these methods, we found that the cross-contribution compensating multiple standard normalization (ccmn) method, followed by square root data transformation, was most appropriate for a well-controlled study such as the milk study dataset. Regarding the lupus nephritis cohort study, only ccmn normalization could slightly improve the data quality of the noisy cohort. Since the assessment accounted for the resemblance between processed data and the corresponding absolute quantified data, our results denote a helpful guideline for processing metabolomic datasets within a similar context (food and clinical metabolomics). Finally, we introduce Metabox 2.0, which enables thorough analysis of metabolomic data, including data processing, biomarker analysis, integrative analysis, and data interpretation. It was successfully used to process and analyze the data in this study. An online web version is available at http://metsysbio.com/metabox.

## Introduction

Metabolomic analysis is widely accepted as a reliable technology for investigating biochemical activities within a cell or tissue of a living organism, and it has been used to address various questions in biology, drug metabolism, food and nutrition, natural products, and biomedicine [1–3]. Typically, the metabolite level in a sample can be determined quantitatively or semiquantitatively. Metabolomic quantitative analysis (absolute quantification) aims to ensure the comparability of metabolite concentrations from measurements obtained at different times or locations. On the other hand, semiquantitative analysis (relative quantification) determines the ratio of metabolite intensity from different samples [4, 5]. Therefore, the absolute concentrations of metabolites represent a benchmark dataset that allows an unbiased comparison across different studies. Due to the limited availability of reference standards, most metabolomic studies are conducted in a semiquantitative manner. However, the inability to compare or correlate the results from different studies remains one of the major limitations of semiquantitative analysis [6]. This is a primary roadblock in the development of metabolomics research. It is therefore essential to encourage the metabolomics community to increase focus on quantitative analyses.

Data processing (DP) plays an important role in semiquantitative and quantitative analyses; the procedures include imputation, normalization, transformation, scaling, and combinations thereof [7]. To date, numerous DP methods have been proposed in metabolomic studies [7–10], each with distinct advantages and pitfalls. Therefore, thorough method evaluations are crucial to pinpointing the best-performing process for a given metabolomic study. Many studies have evaluated and compared DP strategies based on different perspectives, including the normality structure of the data, changes in global variations, reduction of intragroup distance, univariate or multivariate analysis, consistent ranks of putative markers, and classification accuracy [11–15]. DP methods are context dependent and not determined by a sole criterion.

Since quantitative analysis is not applicable in every metabolomic study, choosing proper DP schemes for polishing the peak areas is crucial to best estimate the true metabolite levels. This study aimed to employ another strategy to assess the performance of well-known DP methods. The most desirable DP scheme is the one that yields identical statistical results between the processed data and its quantitative companion. The results obtained constitute a useful and unbiased reference for DP recommendations. The impact of DP method on data distribution, fold-difference patterns between metabolite pairs, and sample variance need to be studied. The DP schemes investigated in this study covered internal standard (IS)–based normalization, transformation, scaling, transformation followed by scaling, and combinations. We used 2 metabolomic datasets representing different types of data: data relating to a food product with definitive markers and clinical metabolomic data with indistinct variations.

Last, we introduced an updated version of the R package Metabox [16] to consolidate a state-of-the-art set of methods for metabolomic analysis from several R packages. Metabox 2.0 enables the in-depth analysis of metabolomic data covering the DP steps, biomarker identification, integrative analysis of multiple data types, and functional interpretation. The software is assembled with ready-to-use R functions that are highly flexible for programming tasks and have broad application potential. This tool was used for all processing steps and analyses in this study.

## Materials and methods

### Metabolomic datasets

Quantitative and semiquantitative metabolomic data were obtained from our recent studies [17, 18]. The first dataset (study I) included the nutrient metabolite composition of various retail milk samples purchased in Thailand [18]. In this study, the analysis was focused on 16 fatty acids (FAs) from 4 milk types: whole bovine milk (*n* = 13), bovine lactose-free milk (*n* = 6), soy milk (*n* = 7), and almond milk (*n* = 3). Each sample was analyzed in triplicate. The dataset contained 10 quality control (QC) samples pooled from a mixture of all the milk samples. The FAs were acquired using gas chromatography coupled to a time-of-flight mass spectrometer (GC-TOFMS; Pegasus BT; Leco Corp., St. Joseph, MI, USA).

The second dataset (study II) contained information on urine samples collected from Ramathibodi Hospital, Thailand [17]. This was done with approval from the Faculty of Medicine Ethics Committee, Ramathibodi Hospital, Mahidol University, Bangkok, Thailand. The urine samples were acquired from 53 healthy subjects (N) and 63 patients with lupus nephritis (LN). The metabolites of the kynurenine pathway (KP) were measured using an ultra-performance liquid chromatography platform coupled to a Xevo TQ-S tandem mass spectrometer (LC-MS/MS) and interfaced by an electrospray ionization source (Waters, Milford, MA, USA).

The mass spectrometry (MS) data from both studies were preprocessed and quantified as described in previous publications [17, 18]. The concentration of each FA was normalized by its molecular weight (µmol), allowing quantitative comparison across studies. The concentration of KP metabolites was normalized by the concentration of urinary creatinine. This follows the standard practice of adjusting the concentration of a metabolite to creatinine filtration in nephrotic syndromes [19, 20]. Additionally, missing value imputation was performed on the milk dataset before data analysis. A minimum value of each metabolite was imputed to a metabolite with missing values higher than 30% groupwise. This step was needed because of the true-negative absence of metabolites under specific conditions, as defined by concentrations that were below the detection limit [21]. If applicable, the nondetected metabolites at random (the percentage of nondetected metabolites <30%) were then imputed by the random forest (RF) method.

### Data processing schemes

This study evaluated the DP schemes commonly applied in a general metabolomic workflow [7]. This included normalization, transformation, scaling, and their combinations.

### Cross-contribution compensating multiple standard normalization

Cross-contribution compensating multiple standard normalization (ccmn) is an IS-based normalization in which metabolite abundances are estimated proportionately to a known IS quantity [22]. Additionally, it considers systematic error and study factors as independent sources of variation on ISs; important information is unaffected by normalization [22]. In contrast to a closely related method, such as normalization using an optimal selection of multiple internal standards (nomis), this method removes unwanted systematic variation based on the variability of single or multiple ISs [23].

### Data transformation

Transformation aims to reduce data skewness, fix heteroscedasticity, and turn multiplicative metabolite relationships into additive relationships [24]. Six transformation methods were assessed in this study, including cube root (cube), logarithm (log2 and log10), generalized log (glog2 and glog10), and square root (sqrt) transformations (Supplementary Table S1). Transformations can reduce the differences between large and small values, whereby large values are scaled down much more than small values [24]. These transformations lead to a depletion in right skewness, which is an observed characteristic of omics data such as metabolomic and transcriptomic data [25]. The cube and glog transformations accept zero and negative values, whereas the sqrt transformation can only manage zero values. In contrast, log transformations can only handle nonzero and nonnegative values. The glog transforms the data using a specific parameter for each dataset [26]. Additionally, it focuses on stabilizing data variance (i.e., keeping the variance constant and independent from the mean) [26, 27].

### Data scaling

Scaling reduces the fold difference between metabolite concentrations based on scaling factors [24]. This is unlike the pseudo-scaling effect of transformations. Here, a scaling factor is determined explicitly for a particular metabolite. This study compared 6 scaling methods: auto, level, pareto, power, range, and vast scaling (Supplementary Table S1). The auto, pareto, range, and vast scaling estimates are scaling factors that are based on data dispersion. In contrast, level scaling is based on the mean value [24]. Power scaling performs an average subtraction in combination with the sqrt transformation [28].

### Analysis workflow

**Figure 1:**
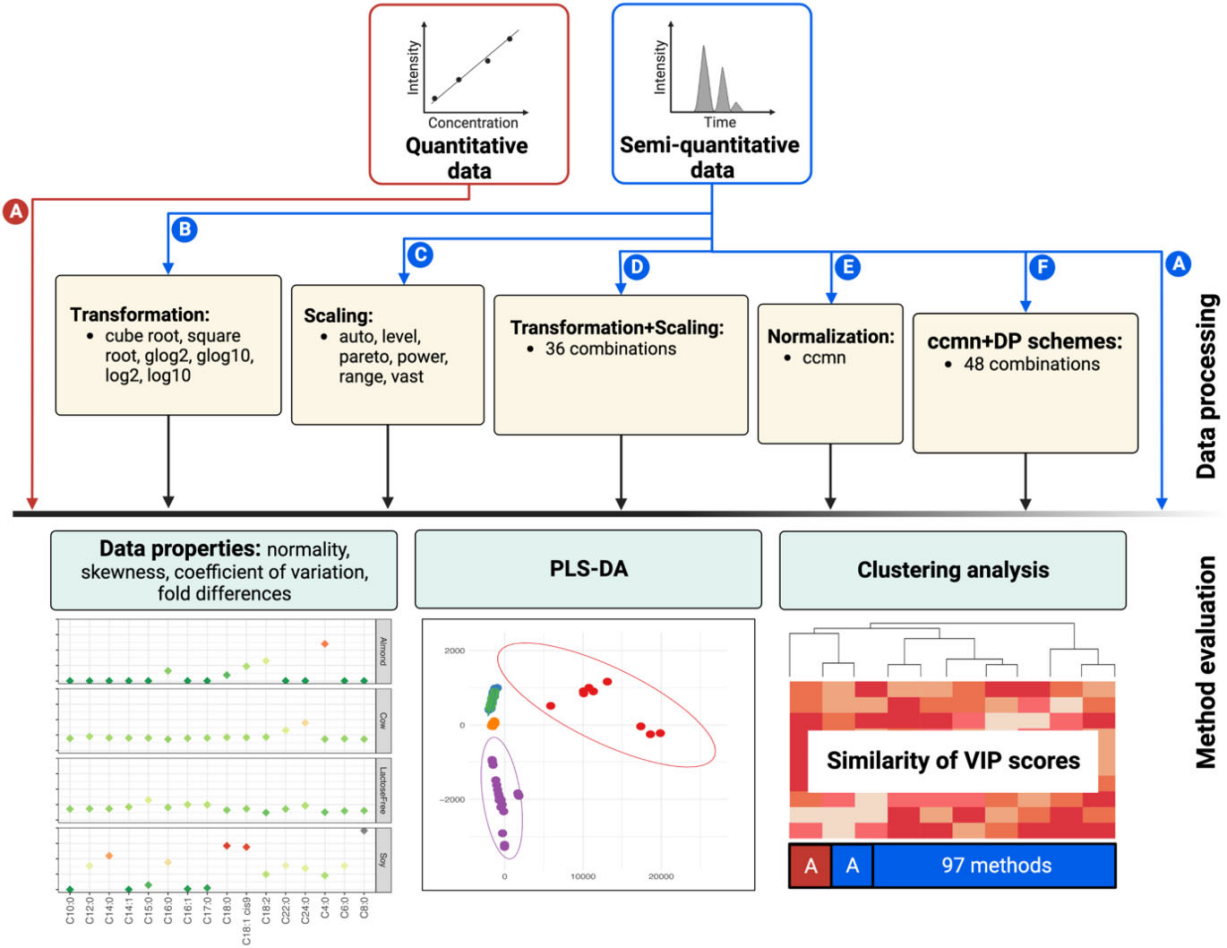
Analysis workflow. Different DP schemes were performed in this study, including (A) no processing, (B) transformation, (C) scaling, (D) transformation followed by scaling, (E) normalization by ccmn, and (F) consisting of ccmn + transform, ccmn + scale, and ccmn + transform + scale. The methods of each DP scheme are listed, and the number shown represents all combinations of these methods. The DP schemes were applied to semiquantitative or peak area data (blue), and the method evaluations were then performed. This included effects on data properties and PLS-DA. For each dataset, the resulting VIPs from PLS-DA were compared to those of the quantitative data (red).

Different DP schemes were investigated using study I [18] and study II [17] datasets that comprised both quantitative (the absolute levels) and semiquantitative (the peak areas of metabolites) results. Key DP schemes were performed to evaluate their effect on the peak area data, which included (A) no processing (raw area), (B) transformation, (C) scaling, (D) transformation followed by scaling, (E) IS-based normalization by the ccmn method, and (F) ccmn normalization combined with transformation, scaling, or a combination of both (Fig. 1). The ccmn normalization was performed using the *normalize_input_data_byqc* function from the R package Metabox 2.0 (RRID: SCR_024,443). The function was implemented from the CRMN R package for normalization of metabolomics data [22]. Heptanoic methyl ester and anthranilic acid C_13_ were used as an IS in study I and study II, respectively. Known amounts of ISs were added to samples before sample preparation, so that metabolite peak areas were normalized with respect to the responses of the ISs.

In total, 97 processed datasets were analyzed, and unprocessed data were considered. We evaluated the influence of the DP methods and their combinations on different aspects, including normality, skewness, coefficient of variation (CV), the trend of fold differences, sample heterogeneity, and multivariate analysis outputs.

The normality test and measures of skewness were computed by the Shapiro–Wilk normality test [29] and the skewness function of the e1071 R package [30], respectively. A *P* value >0.05 indicates a normal distribution, and the symmetric skewness ranges from −0.5 to 0.5. The CV for a metabolite is the ratio of the standard deviation to the mean within a group. The fold and directional differences of a metabolite from a reference were calculated. Since most DP methods strongly affect highly abundant metabolites, the metabolite with the highest level was used as a reference point. An across-group relative log abundance (RLA) plot was applied to explore the grouping structure, outliers, and variation within each group. Each metabolite was standardized by subtracting the median from across all groups [31]. A principal component analysis (PCA) was performed to visualize the major variations in the data regarding the biology of interest.

Moreover, the effects of various DP methods on the partial least squares–discriminant analysis (PLS-DA) in comparison to the absolute concentration (CONC) data were examined. The variable importance in projection (VIP) of a metabolite indicates its degree of contribution to the variance in the PLS model [32]. The similarity between the resulting VIPs from the CONC data, raw area data, and processed data was computed. The similarity between the 2 approaches, *x* and *y*, was calculated using Euclidean distance according to the following equation (1):


(1) \begin{eqnarray*}
\textit{Similarity}{\mathrm{\ }}\left( {x,y} \right) = {\mathrm{\ }}1 - \sqrt {\mathop \sum \limits_i^n {{\left( {{x}_i - {y}_i} \right)}}^2}
\end{eqnarray*}

For method *x*, we denoted the VIP score of the ${i}^{th}$ metabolite as ${x}_i$, where $i = 1,\ 2,\ \ldots ,\ the\ \textit{number}\ of\ \textit{metabolites}\ ( n )$. The same definition was applied to method *y*. Hierarchical clustering of the VIP scores was performed to infer the grouping of the DP schemes. The ComplexHeatmap R package [33] was used for clustering analysis. All DP tasks, PCA, PLS-DA, and plot generation were performed using the R package Metabox 2.0 DP and analysis pipeline.

### Implementation of Metabox 2.0

Metabox 2.0 is a standard R package developed from R version 4.2.0, providing a substantial update to the first Metabox version [16]. An extensive collection of R packages for metabolomic analysis is included (Supplementary Table S1). We enclosed the sequences of DP and analysis tasks in R functions. A graphical user interface (GUI) is implemented with the R package Shiny [34]. An overview of the analysis pipelines is illustrated in Supplementary Fig. S1.

### Data processing and analysis pipeline

This analysis pipeline supports DP and consecutive data analyses, including essential statistical analyses and biomarker discovery (Supplementary Fig. S1A). The DP module includes all major metabolomic DP tasks, starting with missing value imputation, normalization, transformation, and data scaling. A collection of commonly used methods is integrated into Metabox 2.0 (Supplementary Table S1). Three types of imputation methods are provided, including single value, local similarity, and global structure approaches [21]. The normalization module covers IS-, QC sample– and data-based approaches, which aim to eliminate unwanted errors while maintaining crucial biological variation [8, 31]. IS-based and QC sample–based normalization rely on spike-in ISs and the intensity of QC samples, respectively [8]. Meanwhile, the data-driven normalization summarizes a sample-specific factor for the adjustment [31]. The transformation methods for decreasing right skewness and scaling methods based on either data dispersion or mean value are included. When performing both data transformations and scaling, the differences in magnitude between large and small metabolite values are adjusted, so that those metabolites are comparable. In total, there are 10 imputations, 3 IS-based normalizations, 2 QC sample–based normalizations, 12 data-driven normalizations, 6 transformations, and 6 scaling methods.

The statistical analysis module comes with a collection of univariate analysis methods. These are statistical hypothesis testing methods and post hoc tests covering parametric and nonparametric tests, a pairwise correlation analysis, and linear mixed modeling from the lmm2met package [7] (Supplementary Table S2). For multivariate analysis, both unsupervised and supervised multivariate analyses are included, incorporating PCA, PLS-DA, and orthogonal PLS-DA (OPLS-DA) implemented from the ropls package [35].

The biomarker analysis module supports regression and classification analyses using the PLS or RF approach. We incorporate recursive variable elimination within a repeated double cross-validation (repCV) approach from the MUVR package [36] to identify informative metabolites. The algorithm addresses prediction accuracy, model overfitting, and optimally relevant metabolites.

### Data integration pipeline

Metabox 2.0 supports the joint analysis of multiple data types, such as omics and other phenotypic data (Supplementary Fig. S1B). The multiblock PLS-DA (MBPLSDA) pipeline from the mbpls package [37] is assimilated into the integrative analysis module, focusing on the multivariate modeling of concatenated data blocks by considering the specific data structure of each block. This method allows the estimation of both variable and block importance.

### Data interpretation pipeline

This pipeline includes well-established methods for functional interpretation in the context of metabolic pathways and chemical classes (Supplementary Fig. S1C). The set enrichment analysis and overrepresentation analysis can be performed with a comprehensive collection of methods from the piano package [38], as implemented in Metabox 1.0 [16]. Moreover, integrated pathway overrepresentation analysis uses Fisher’s method to combine *P* value outputs from the hypergeometric test. The KEGG database [39] is used for pathway information, whereas chemical classes of metabolites are based on the HMDB chemical taxonomy [40].

## Results

### The effects of data processing on the semiquantified fatty acids in milk samples

All 16 FAs were quantified in whole and lactose-free bovine milk (Supplementary Fig. S2). However, some FAs, including C10:0 and C14:1, were not detected in the plant-based milk products (soy milk and almond milk). C6:0, C14:0, C22:0, and C24:0 were not present in almond milk. C8:0, C12:0, C15:0, C16:1, and C17:0 were detectable in soy milk but absent in almond milk. These metabolites were imputed by their minimum value prior to DP. As reported by Jariyasopit et al. [18], C16:0 (palmitic acid) and 2 unsaturated FAs (UFAs) (C18:1 *cis*-9 [oleic acid] and C18:2*n–*6 or C18:2 [linoleic acid]) were at their highest concentrations (mg/L) in bovine milk, almond milk, and soy milk, respectively. The amount of C18:1 *cis*-9 in almond milk was markedly high (14,230.98 ± 4057.15 µmol).

### Effects on data properties

**Figure 2:**
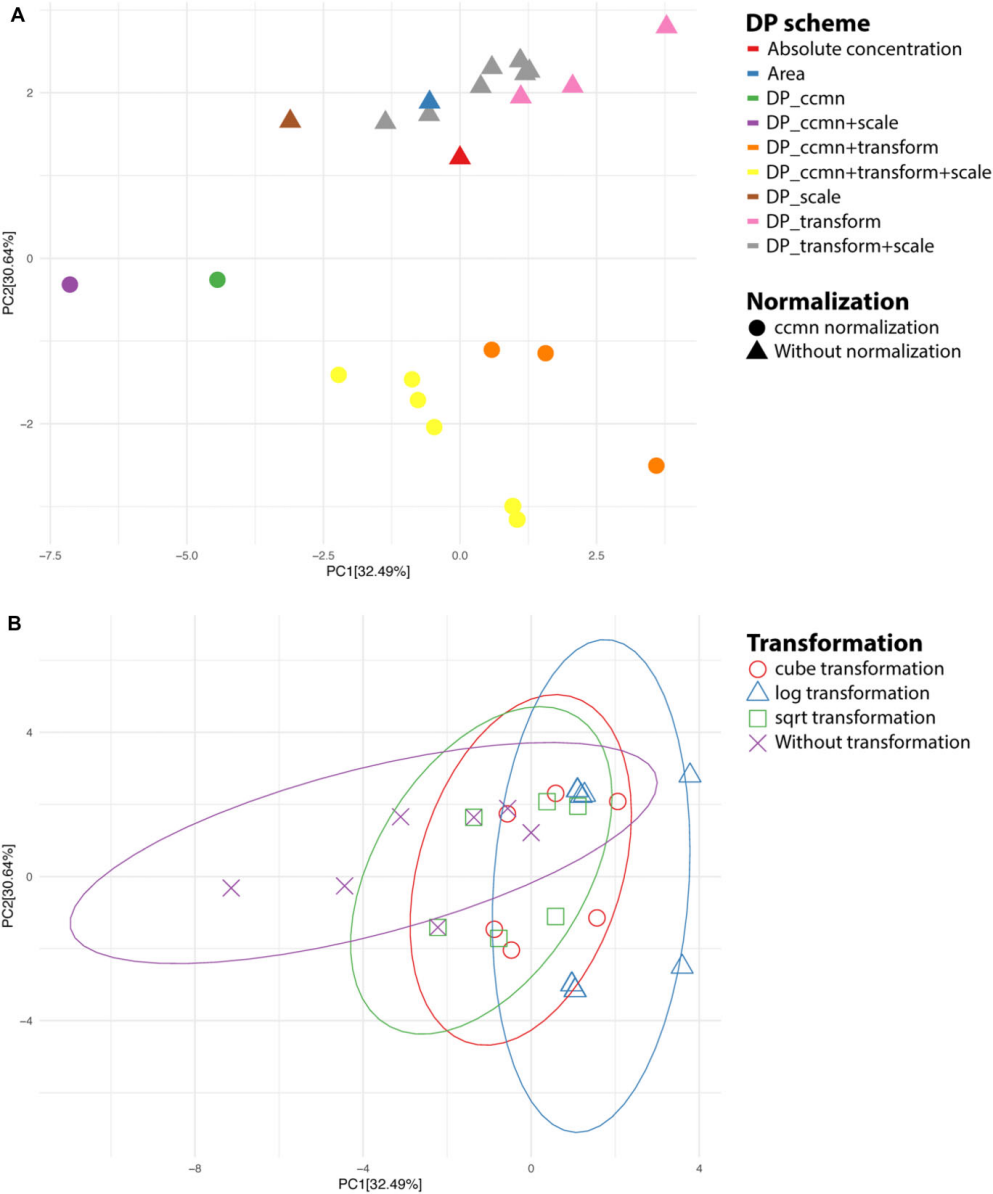
PCA score plots based on the data properties of absolute concentration, unprocessed (raw area), and processed milk data. (A) Major separation based on DP schemes and (B) major separation based on transformation methods. The data properties included normality, skewness, and coefficient of variation.

Initially, we explored the effects of each DP scheme on the basic properties of the milk dataset. For each DP scheme, we considered the number of normally distributed, positively skewed, and negatively skewed metabolites and the CV in each sample group (Supplementary Table S3). All FAs in the QC samples were normally and symmetrically distributed for the CONC data. Most FAs in bovine milk and soy milk were right-skewed (i.e., a few FAs were highly abundant), while the FAs in bovine lactose-free milk were mostly normally distributed. The largest and smallest CVs were present in soy milk and almond milk, respectively. Similar aspects were observed in the peak area data. The PCA plot showed that the ccmn method was the main factor contributing to separation among the DP schemes, with and without this normalization (Fig. 2A). The number of normally distributed metabolites in the almond milk dramatically increased after normalization, and most FAs became slightly negatively skewed. The basic properties of the quantified and raw area data were closer to those of the unnormalized datasets. Each DP scheme resulted in an apparent cluster. The transformations increased the number of normally distributed metabolites in bovine milk. The area processed by the scaling scheme was separated from those altered by the transform + scale and the transformation, with and without normalization. All scaling methods returned similar data properties, with power scaling being slightly different. After power scaling, the area data had the same data properties as those after sqrt + auto, level, pareto, range, or vast scaling (Fig. 2B). All glog- and log-based transformations produced similar data properties. However, the cube-, sqrt-, and log-based transformations were attributed to slightly different data properties, particularly the distribution of metabolites in bovine milk. As such, we noticed a separation among the cube, sqrt, and both log-based transformation schemes.

### Effects on multivariate analysis

**Figure 3:**
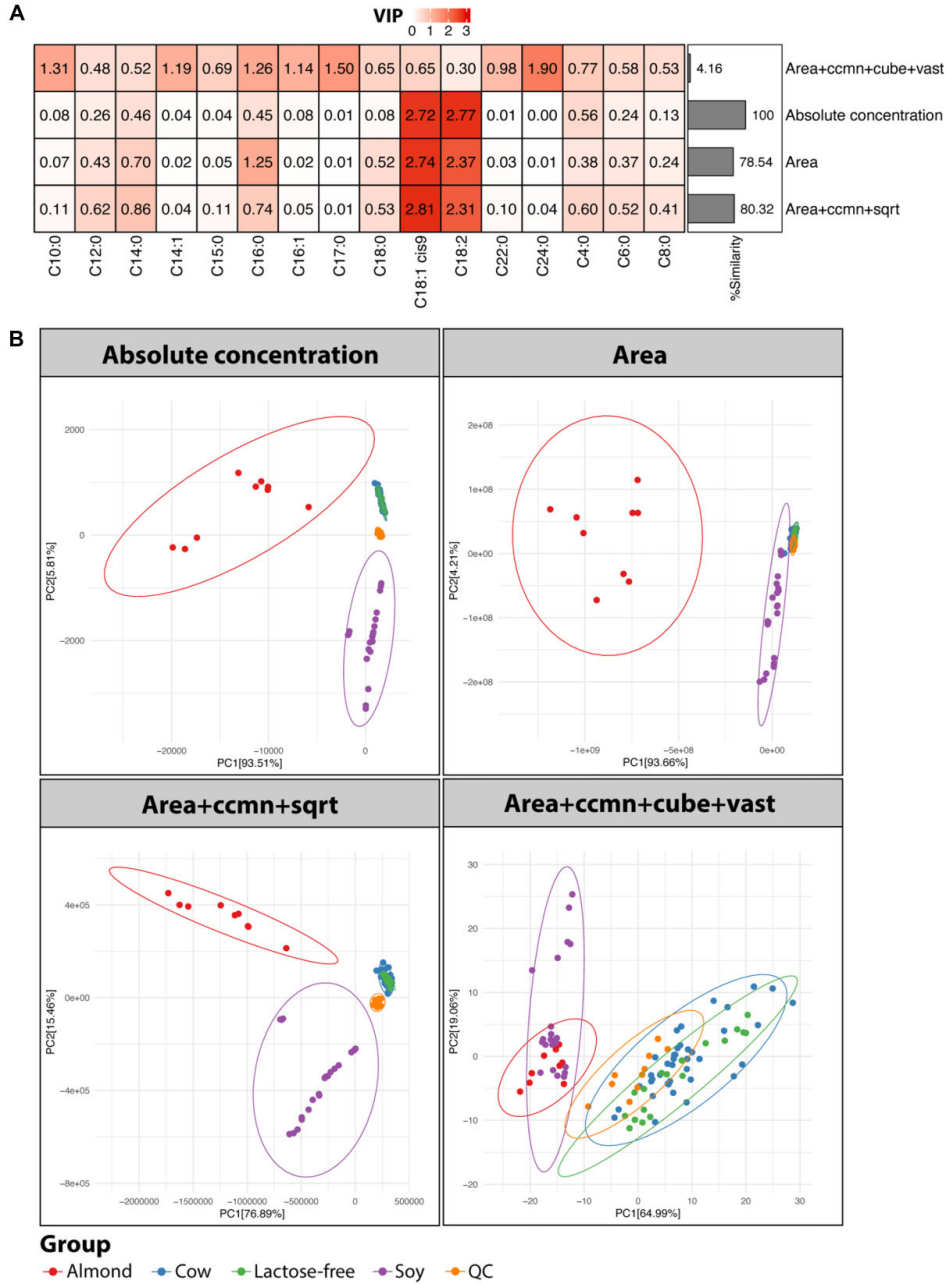
Comparisons of selected DP schemes to absolute concentration and the raw milk area data represented by (A) clustering of VIPs and (B) PCA plots.

PLS-DA was performed on 97 processed datasets, the absolute FA concentration and peak area datasets. Cluster analysis of the resulting VIPs revealed grouping of the CONC data, raw area data, ccmn, ccmn + pareto, ccmn + power, and ccmn + sqrt processed data (Supplementary Fig. S3). These DP schemes formed a separate branch from the DP, involving log-based transformations, auto, range, or vast scaling. The VIP scores of the data that had undergone ccmn + power and ccmn + sqrt processing were identical. Moreover, they were similar to the VIPs from the quantified data (Fig. 3A and Supplementary Table S4, similarity = 80.32%). The VIPs from the unprocessed area data were approximately 20% different from the original CONC data. In contrast, when using the glog-, log-, auto-, range-, and vast-based DP methods, the VIP similarity was reduced to below 40%. Any DP counting in the vast method led to a low similarity of approximately 10% or less. The ccmn + cube + vast was the least alike (similarity = 4.16%).

C18:1 *cis*-9 and C18:2*n–*6, the important plant UFAs [41], were the discriminant metabolites (VIP ≥1.5) obtained from the PLS-DA on the CONC and raw area data (Supplementary Fig. S3). Moreover, both UFAs were identified from the data processed by sqrt or cube transformation, level, pareto, or power scaling. VIP scores increased slightly when ccmn normalization was applied together with sqrt, cube, pareto, or power. Combining these transformation and scaling methods led to lower VIPs, particularly the VIP of C18:2. C16:0 was an additional discriminant for the plant UFAs in the ccmn-normalized data. The DP schemes involving vast scaling failed to identify C18:1 *cis*-9 and C18:2*n–*6, as their VIPs were less than 1.0. The number of FAs with VIP >1.0 was increased by glog- or log-based transformation, yet it diminished the importance of the plant UFAs in the PLS model.

The PCA plots showed a clear partitioning of the almond milk, soy milk, and bovine milk, except for the ccmn + cube + vast processed data (Fig. 3B). This DP task mitigated the variance between soy milk and almond milk. Since C18:1 *cis*-9 and C18:2*n–*6 were the important plant UFAs, a clustering of plant-based milk products was observed. Meanwhile, the whole and lactose-free bovine milk were clustered together because their FA profiles were similar (Supplementary Fig. S2). The key distinction between these bovine milk types was the absence of lactose in lactose-free bovine milk [18]. The variability explained by the first and second principal components (PCs) was 99.32%, 97.87%, 92.35%, and 84.05% for the CONC, raw area, ccmn + sqrt, and ccmn + cube + vast processed data, respectively. Though the observed variation of ccmn + sqrt was less than that of the raw area data, the original structure of variation was more preserved with ccmn + sqrt processing. The milk dataset possessed intragroup variability, which was still visible after processing with ccmn + sqrt (Supplementary Fig. S4A). However, this within-group variation was inflated, and the plant-based samples displayed a right-skewed distribution after applying ccmn + cube + vast.

For the CONC and peak area data, the mean level of the CV (mCV) in each milk type was as follows: soymilk > bovine milk > bovine lactose-free milk > QC sample > almond milk (Supplementary Fig. S4B). The ccmn + sqrt method could lessen metabolite dispersion, in contrast to the ccmn + cube + vast. In sequential order, it enlarged the mCV of the QC, bovine lactose-free milk, bovine milk, soy milk, and almond milk samples. In addition, the trend of the fold differences between C18:1 *cis*-9 and the other FAs in all milk types was substantially altered by ccmn + cube + vast (Supplementary Fig. S4C). Specifically, the C18:1 *cis*-9 abundance became less than the FAs C8:0, C10:0, C14:1, C15:0, C16:1, and C17:0 in both bovine milk types, as opposed to the original CONC data. The amount of C18:1 *cis*-9 was higher than that of C16:0 in the bovine milk samples and lower in the almond milk. In the case of the ccmn + sqrt method, overall fold differences were maintained and comparable to the original CONC data.

### Effects on multivariate analysis in the absence of highly abundant metabolites

The absolute amounts of C18:1 *cis*-9 and C18:2 were relatively high compared to those of the other FAs (Supplementary Fig. S2). They were the main discriminants between almond milk, soy milk, and plant-based and bovine milk in the PLS-DA (Fig. 3). The previous section showed a case study involving variables with strong relative responses of a biological factor (milk types). We continued our evaluation of the milk dataset, excluding the major metabolites C18:1 *cis*-9 and C18:2. This was to represent a case study without extreme relative responses.

From VIP clustering, the CONC, raw area, ccmn-normalized, and pareto-scaled data were grouped and formed the closest linkage to a cluster containing either the cube, sqrt, or power processing alone or in combination with ccmn normalization (Supplementary Fig. S5). Elements in this group included ccmn + pareto, with/without cube or sqrt transformation, and ccmn + sqrt + power. These DP schemes formed a distant branch from the DP tasks embracing auto, level, range, vast scaling, or log-based transformations. In particular, the percentage of VIP similarity was less than 40%. The VIPs from glog2 + vast processed data were the most dissimilar (Supplementary Table S5, similarity = 31.54%), and only C24:0 was the discriminative metabolite from this method (Supplementary Fig. S6A). The most similar VIP scores were the VIP scores of the peak area (similarity = 83.28%), whereas the VIPs from the data processed by the ccmn + power or ccmn + sqrt processing were slightly less identical (similarity = 80.35%). The discriminant metabolites C16:0 and C4:0 were commonly observed from the CONC, area, and ccmn + sqrt processed data. C14:0 was the additional discriminant for the CONC and ccmn + sqrt processed data, while C18:0 was identified for the peak area data.

The apparent separation between plant-based and bovine milk was observed from the PCA plots, except for the area and glog2 + vast processed data (Supplementary Fig. S6B). Even without the plant UFAs, the clustering of plant-based and bovine milk types persisted. This was because of the differences in FA compositions as reported by Jariyasopit et al. [18]. Both bovine milk types were more enriched with saturated FA compared to plant-based milk products (Supplementary Fig. S2). The data structure of the CONC data was more preserved in the ccmn + sqrt processed dataset. Meanwhile, the PCA plots of the area data with (Fig. 3B) and without the plant UFAs (Supplementary Fig. S6B) were comparable. The cumulative variance explained by PC1 and PC2 for the CONC, area, ccmn + sqrt, and glog2 + vast processed data was 97.43%, 99.47%, 97.40%, and 83.56%, respectively. Although we noticed a clustering of plant and bovine milk types by the glog2 + vast method, high variance was introduced within the QC and bovine milk samples. Accordingly, we detected a substantial impact on the data distribution, outliers (Supplementary Fig. S6C) and metabolite deviation from its mean (Supplementary Fig. S6D). In contrast to ccmn + sqrt, fold difference tendencies between C16:0 and the other FAs, in all milk types, were markedly influenced after applying glog2 + vast (Supplementary Fig. S6E).

### Data processing effects on the semiquantified metabolites in urine samples

Similar aspects were performed to evaluate and compare the effects of the DP methods on 8 KP metabolites in urine samples (study II). For CONC and raw area data, almost all metabolites were positively skewed, and the mCV of the LN samples was slightly higher than that of the normal samples (Supplementary Table S6). The basic properties of the quantified and ccmn-normalized data were closer than those of the other datasets. However, the combined ccmn with transformation, or transform + scale, was not the major factor influencing the separation, as observed in study I (Supplementary Fig. S7). When applying the transformation, scaling, or transform + scale schemes, the DP effect on the urine data properties appeared consistent with the milk sample data.

We observed clustering of VIPs from the sqrt, pareto, power, ccmn + cube, ccmn + sqrt, ccmn + pareto, ccmn + power, and area data (Supplementary Fig. S8). They formed a distinct branch from the DP schemes, including glog, log, auto, level, range, and vast. Meanwhile, the VIPs from the CONC and the ccmn-normalized data were distinguished from those of the other methods. The VIP scores from the unprocessed peak areas were 55.53%, identical to the CONC data (Supplementary Table S7). The VIP scores of the ccmn-normalized data were the closest to those of the quantified data (Supplementary Fig. S9A, similarity = 62.36%). In contrast, the ccmn + level DP led to the least similar VIPs (similarity = 41.62%). Tryptophan was the discriminant metabolite (VIP ≥1.2) observed in the CONC data, raw areas, and ccmn-processed data. Kynurenic acid was identified as an important metabolite in both the CONC and the ccmn-normalized data. Picolinic acid (VIP >1.6) was the discriminative metabolite observed in the datasets that applied the DP glog, log, auto, level, range, or vast methods (Supplementary Fig. S8). From the CONC data, this metabolite possessed a low VIP weight (VIP = 0.03). In contrast, 3-hydroxykynurenine was only reported from the CONC data (VIP = 1.52) and was absent in the other datasets.

The urine samples from healthy subjects and LN patients mainly overlapped (Supplementary Fig. S9B) and showed high within-group variation (Supplementary Fig. S9C). However, the clustering of different subject groups was largely due to tryptophan (Supplementary Fig. S9A). This metabolite was reported as a potential biomarker for chronic kidney diseases [17]. The ccmn method improved the explained variance in the first PC (PC1 = 81.02%) compared to the raw area data (PC1 = 74.78%). In contrast, when using the ccmn + level method, we observed large influences on the sample distribution, metabolite variation, and fold differences between tryptophan and the other metabolites (Supplementary Fig. S9C–E). When using this method, the PC1 and cumulative variance were 41.77% and 61.56%, respectively.

### IS-based normalization performance is dependent on the type of biological factors

**Figure 4:**
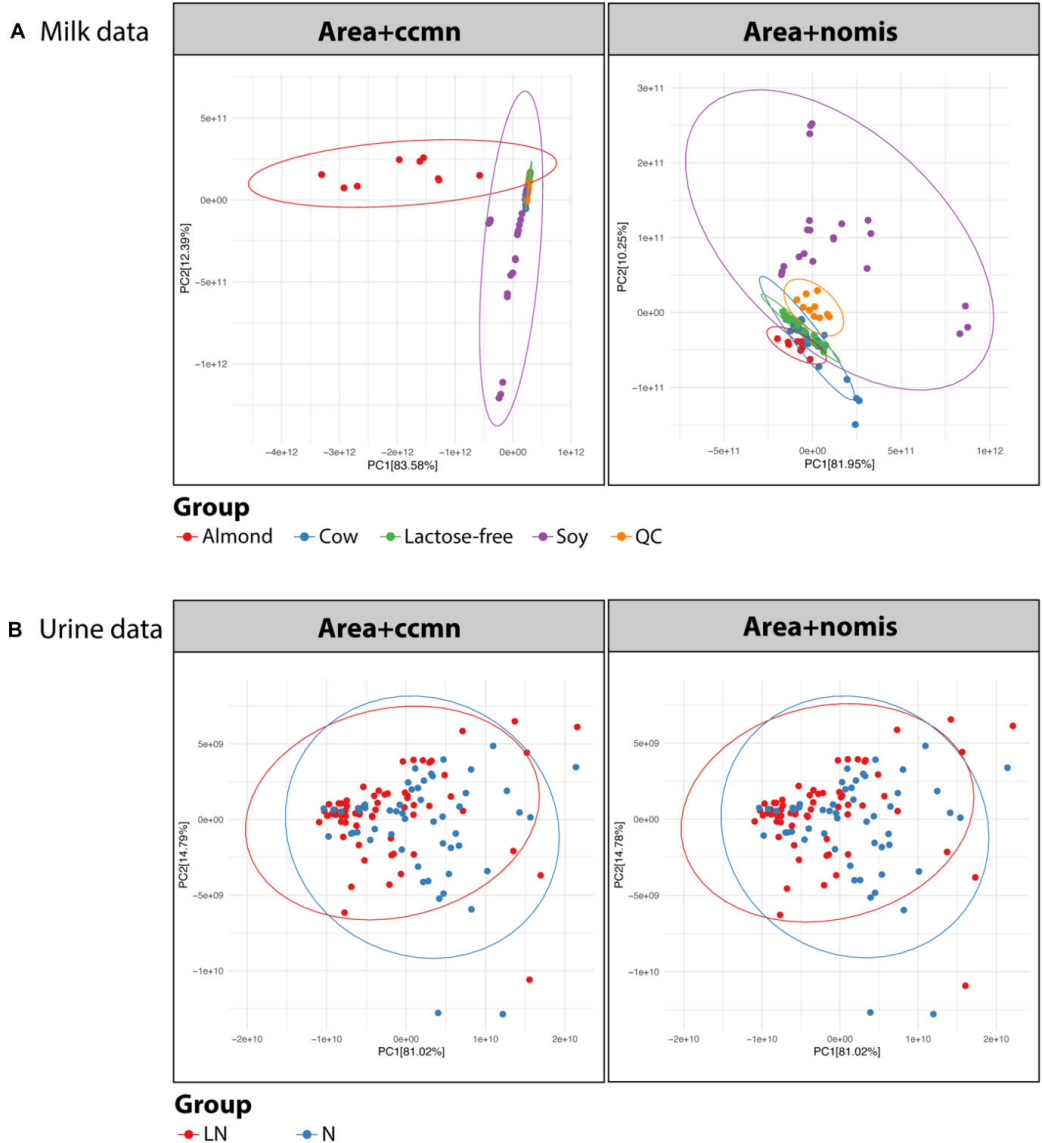
Effects of the ccmn and nomis normalization methods on the (A) milk data and (B) urine data. Color coding indicates sample groups, including the types of milk, the urine samples from healthy subjects (N), and patients with lupus nephritis (LN).

Two commonly used IS-based normalization methods, ccmn and nomis, were further evaluated using milk and urine datasets. The milk dataset has a definitive biological effect, whereas the urine samples comprise many unknown individual variations. In this assent, the informative variation of milk samples was retained by the ccmn method, unlike the nomis method (Fig. 4A). The variances explained by the first and second PCs were 95.97% and 92.20% for the ccmn- and nomis-normalized data, respectively. The first PC presented the differences between almond milk and soy milk for the ccmn-processed data. This aspect was invisible in the nomis-normalized data. In the case of the urine dataset, we observed that the ccmn method performed similarly to the nomis method (Fig. 4B). The groups of healthy and LN samples were slightly separated. Within the LN group, variation was slightly reduced after normalization. Subject-specific variations and the presence of outliers are common in clinical metabolomics. Moreover, the ccmn method only assumes linear relationships between measured metabolites and experimental factors, which is not always the case in metabolomics [22]. Therefore, the metabolite and IS interferences in the urine matrix may not be thoroughly corrected by the ccmn method.

### Metabox 2.0: enhancing metabolomic data analysis, integration, and interpretation

**Figure 5:**
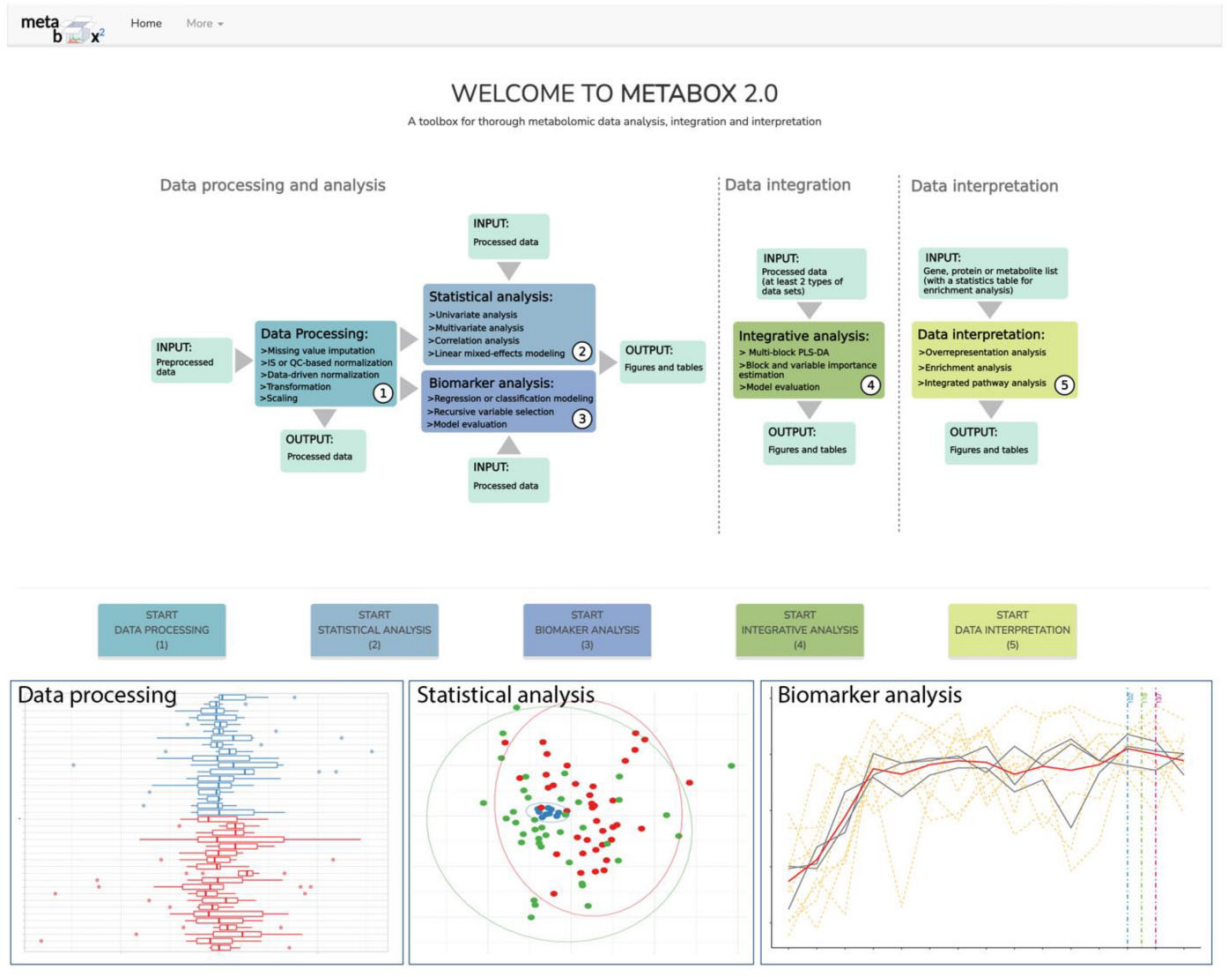
Metabox 2.0 GUI and example outputs from the data processing, statistical analysis, and biomarker analysis modules.

Metabox 2.0 is implemented as a standard R package. The current version has undergone significant redesign and updates since Metabox 1.0 [16], highlighting the analysis of metabolomic data from DP steps to biomarker identification and allowing the joint analysis of multiple data types, such as LC- and GC-MS metabolomes, metabolomic and transcriptomic datasets, or metabolomic and clinical data. Three analysis pipelines are organized as separate modules (Fig. 5 and Supplementary Fig. S1). A series of scripts for a particular task is encoded in a ready-to-use R function, allowing the implementation of customized workflows. The key features of this version include (i) a collection of state-of-the-art methods for end-to-end metabolomic data analysis, (ii) normalization methods for cohort- and laboratory-scale metabolomic studies, (iii) univariate analysis for 1 or multiple factors, (iv) multivariate modeling for both classification and regression, (v) machine learning (ML)–based biomarker analysis with minimizing model overfitting and false-positive rates, (vi) cross-domain data integration, (vii) data interpretation in the context of metabolic pathways and chemical classes, (viii) various kinds of plots for data exploration, and (ix) an intuitive GUI for bench biologists (Fig. 5). This GUI version supports typical analysis and allows broader usability as a hosted web application on the server. The integrative exploration of multiomic levels in biological networks is excluded in this version because it requires the preinstallation of a specific graph database system.

For metabolomic analysis, this tool serves as an alternative to closely related software such as MetaboAnalyst [42], iMAP [43], NOREVA [28], XCMS Online [44], MZmine 3 [45], and OUKS [46]. Comparison of the main features with other tools is summarized in Supplementary Table S8. Metabox 2.0 covers more DP methods and is equipped with a tool for integrative analysis of omic and nonomic data. It is an open-source R package freely accessible from our GitHub [47] under the GPL-3 license. Furthermore, an online web version is publicly available from our website [48].

## Discussion

In the field of metabolomics, quantitative analysis is important for understanding cellular metabolism because the abundances of metabolites affect both free energy and metabolic reactions [49]. Nonetheless, a number of obstacles, including the absence of standardized methods and the accessibility of reference standards, lead the majority of metabolomics research to be conducted using a semiquantitative analysis. Although semiquantitative analysis does not provide the true value of a metabolite’s concentration, it is still useful for the discovery of important metabolites in many studies [3]. Often, the potential bias or technical errors introduced by this approach can be removed or minimized through techniques in analytical chemistry and bioinformatics [2, 7]. The major limitation of semiquantitative compared to quantitative analysis is that it is challenging to compare results across different studies, leading to difficulty translating the potential metabolites into practice, especially in clinical research [50].

Quantitative analysis is ideal in metabolomics research; however, the absolute quantification of all metabolites can be challenging. A good DP scheme for raw data processing is essential to improve semiquantitative data such that it resembles quantitative data. In this study, we characterized and compared the semiquantified metabolites after different DP treatments to their quantified counterparts. We covered 3 common scenarios in metabolomics, including a dataset with apparent markers, a dataset with a known biological effect, and a dataset with obscure variations.

The results consistently indicated that normalization and transformation had an impact on the data distribution, skewness, and CV, while scaling only influenced the CV of the data. Apart from 1 exception, power scaling behaved like the sqrt + scale scheme. This is because the method relies on the square root of metabolite intensity along with a mean subtraction [28]. However, these data properties could not directly reflect the final results of statistical analyses (e.g., how many significant metabolites are identified or how much class discrimination is improved by a specific DP method). An understanding of data distribution could guide the choice of subsequent statistical analysis. The change in the CV is an indicator of DP performance in reducing group variation [51].

The use of milk and urine sample datasets represented 2 sides of the story. As a food product, the milk samples were produced in well-controlled environments, while the urine study data were not, even though there were strict inclusion and exclusion criteria in the study cohort. Interindividual variations (e.g., dietary, genetic, and demographic background) were the key unwanted variations in clinical metabolomics [7]. Accordingly, we observed that the PLS-DA result from the milk area data resembled its CONC data more than that of the urine study data. The raw peak areas, excluding the 2 plant UFAs, resulted in the most similar result to the CONC data without any processing task. Due to the numerous unknown sample matrices, the ccmn method performed similarly to nomis normalization in the urine dataset. Overall, ccmn normalization improved the quality of semiquantitative data in every case in our study and is therefore recommended. Furthermore, the ccmn method can segregate IS interference because of the correlation with the factors under study [22, 31]. As such, it can avoid the risk of losing informative variations in the nomis.

The normalization process aims to remove systematic errors and unrelated biological variations (if applicable). However, it cannot scale for magnitude differences among metabolites. When considering the milk sample dataset, which included plant UFAs, the ccmn + sqrt method was suggested. This was because the dataset had a distinct biological factor, and the sqrt transformation had the least effect on the variance structure compared to the other transformation methods. In descending order, the effect size ranged from the log family, cube transformation, and sqrt method. The performance of the scaling methods, power, and pareto was relatively comparable and had a smaller effect on the data variance than the other methods. The vast-scaled milk and level-scaled urine produced the most divergence among VIP results from the CONC data. This is because vast scaling is more suitable for datasets with small induced fluctuations [11, 24], which is not the case in this study. The FAs with a large variation were considered less important, while a low-deviated metabolite became more significant after vast scaling. In contrast to level scaling, this approach is suggested for a study that involves large relative responses of a biological factor [11, 24]. This method failed when using the urine sample data because the signal-to-noise ratio was low. Transformation by log family is a commonly used approach in omic data analysis. Its transformation is stronger than cube and sqrt methods (i.e., transformed data are more divergent from the original). The log transformation performs well for data with constant relative standard deviation [24]. However, it is not always the case in metabolomics, where variance gets larger with an increasing intensity level. The log transformation tends to reduce the large variance for large values, but it rather inflates the variance of metabolites close to zero [52]. In this study, its performance was modest for both milk and urine sample datasets (VIP similarity = 35–45%). By using the log transformation, one needs to balance the trade-off between obtaining more discriminant metabolites and gaining more false positives.

Class separation in multivariate models such as PCA and PLS-DA is attributed to metabolites with high loadings, which are usually proportional to the concentration or magnitude of fold change [24, 52, 53]. These dominant sources of variation could be informative markers or obscure dominators. We showed that, with the presence of very high concentrations of plant UFAs (C18:1 *cis*-9 and C18:2), they were always the main discriminants between almond milk, soy milk, and plant-based and bovine milk. Without both plant UFAs, the second most abundant FAs (C16:0, C4:0, and C14:0) became the key source of variation between plant-based and bovine milk types. However, vast, range, and auto greatly minimized the importance of plant UFAs and resulted in a higher loading for metabolites with low measured levels. Different transformation and scaling methods adjust scale-size effects to a certain degree, allowing low-concentration metabolites to be more important [24]. If a low-abundant metabolite could reflect the variation of interest, it is worth considering the separation of very high-abundant metabolites to enable exploration of low-concentration regions.

In summary, our study showed that the ccmn or the ccmn + sqrt method yielded the most similar PLS-DA results between the quantified and semiquantified equivalents. Given that ccmn outperformed the other methods in this study, various research projects are under way to improve the normalization strategy, that is, the use of quality control metabolites [31], quality control samples [8], subject-specific characteristics [7], and nonlinear modeling [8, 9, 22]. The transform + scale and normalize + transform + scale schemes could not enhance the quality of the semiquantitative data in many cases. This may be because of an excessive alteration in the data variance by both transformation and scaling.

Many studies have compared different strategies for metabolomics data processing [11–15]. However, this is the first study to use quantitative data as a reference point for DP evaluation. Our findings are based on the closest representation of genuine metabolite patterns, which could be a valuable guide for the DP procedure. In practice, the CONC data are not always available for comparison. Therefore, method evaluations, such as those performed in this and the other studies, are highly advised prior to any statistical analysis (e.g., the changes in CV, the fold difference trends, the unsupervised patterns of the PCA score plot, and the clustering of multivariate analysis outputs). Most bioinformatics tools, such as Metabox 2.0, MetaboAnalyst [42], NOREVA [28, 51], and OUKS [46], provide various features to facilitate DP evaluations and comparisons.

## Conclusion

This study reported the effects of DP schemes on data properties and variance structure. Normalization and transformations altered the data normality, skewness, and CV, whereas data scaling only changed the CV. The PCA and the results of PLS-DA were compared between the absolute abundances and the processed peak areas to observe the outcome of different DP schemes on the data variance. The ccmn + sqrt outperformed for the datasets with apparent markers and a known biological effect. Furthermore, the raw area may be used if the samples are from a well-defined experiment and have a known matrix effect. Although the resulting VIPs from the raw peak of urine metabolites were slightly over 50% identical to the absolute levels, IS-based normalization, such as ccmn, was the best option to improve this clinical metabolomics data. Choosing a strong DP method (e.g., log transformation, auto, range, vast, and level scaling) needs careful consideration. These methods have less tolerance for outliers and tend to amplify noise. Our study used another aspect of the DP criteria. The best DP choice allowed the semiquantitative data to mimic the quantitative data. Additionally, we discussed the bioinformatics toolbox, Metabox 2.0, which contains significant updates on the DP tasks, biomarker identification, and integrative analysis, and served as a tool for all analyses in this study.

## Availability of Source Code

Project name: Metabox 2.0

Project homepage: https://metsysbio.com/metabox/index.html

Operating system(s): Platform independent

Programming language: R

Other requirements: None

License: GNU General Public License (v3)

RRID: SCR_024,443

## Supplementary Material

giae005_GIGA-D-23-00257_Original_Submission

giae005_GIGA-D-23-00257_Revision_1

giae005_Response_to_Reviewer_Comments_Original_Submission

giae005_Reviewer_1_Report_Original_Submission Cheng Zhao -- 11/18/2023 Reviewed

giae005_Reviewer_2_Report_Original_Submission Cheng Zhao -- 11/18/2023 Reviewed

giae005_Reviewer_2_Report_Revision_1 Cheng Zhao -- 1/10/2024 Reviewed

giae005_Supplemental_Files

## Data Availability

R code used for this article and intermediary files are available from the *GigaScience* database, GigaDB [54]. The metabox2 package and its full source code are also available from GitHub [47]. The datasets used in this article have been submitted to the Metabolomics Workbench repository [55], study I (ST002902), and study II (ST002874).

## Additional Files

**Supplementary Fig. S1**. Metabox 2.0 analysis pipelines. Metabox 2.0 provides 3 analysis pipelines: (A) data processing and analysis, (B) data integration, and (C) data interpretation. For each pipeline, the analysis module and its major tasks are presented in blue boxes. The data processing and analysis pipeline includes separated modules for data processing, statistical analysis, and biomarker analysis. A comma-separated values (CSV) file is the required input format. The output contains a report file, related figures, and tables.

**Supplementary Fig. S2**. The absolute concentration of 16 FAs in the milk samples.

**Supplementary Fig. S3**. Clustering of VIPs from absolute concentration, unprocessed milk, and processed milk data by different DP schemes.

**Supplementary Fig. S4**. Comparisons of the selected DP schemes to the absolute concentration and unprocessed milk data represented by (A) across-group RLA plots, (B) CV plots, and (C) fold differences of oleic acid to the other fatty acids. The CV of each metabolite is indicated by color coding from green to red. The large CV (CV >1.0) is represented as a dark gray dot. The average CV (mCV) of each group is shown.

**Supplementary Fig. S5**. Clustering of VIPs from absolute concentration, unprocessed milk, and processed milk data without the plant UFAs by different DP schemes.

**Supplementary Fig. S6**. Comparisons of the selected DP schemes to the absolute concentration and unprocessed milk data without the plant UFAs represented by (A) clustering of VIPs, (B) PCA plot, (C) across-group RLA plots, (D) CV plots, and (E) fold differences of palmitic acid to the other fatty acids. The CV of each metabolite is indicated by color coding from green to red. The large CV (CV >1.0) is represented as a dark gray dot. The average CV (mCV) of each group is shown.

**Supplementary Fig. S7**. PCA score plots based on the data properties of absolute concentration, raw area, and processed urine datasets. (A) Major separation based on DP schemes and (B) major separation based on transformation methods. The data properties included normality, skewness, and coefficient of variation.

**Supplementary Fig. S8**. Clustering of VIPs from absolute concentration, unprocessed urine, and processed urine data by different DP schemes.

**Supplementary Fig. S9**. Comparisons of the selected DP schemes to the absolute concentration and unprocessed urine data represented by (A) clustering of VIPs, (B) PCA plot, (C) across-group RLA plots, (D) CV plots, and (E) fold differences of tryptophan to the other KP metabolites. The CV of each metabolite is indicated by color coding from green to red. The large CV (CV >1.0) is represented as a dark gray dot. The average CV (mCV) of each group is shown. Healthy group (N) and lupus nephritis group (LN).

**Supplementary Table S1**. List of DP methods in Metabox 2.0.

**Supplementary Table S2**. List of univariate analysis in Metabox 2.0.

**Supplementary Table S3**. Data properties of the absolute concentration, area and processed milk data.

**Supplementary Table S4**. Similarity of VIPs from the area, and processed milk data compared to the absolute concentration data.

**Supplementary Table S5**. Similarity of VIPs from the milk data set excluded the plant UFAs.

**Supplementary Table S6**. Data properties of the absolute concentration, area and processed urine data.

**Supplementary Table S7**. Similarity of VIPs from the area, and processed urine data compared to the absolute concentration data.

**Supplementary Table S8**. Comparison of Metabox 2.0 with other open source tools.

## Abbreviations

ccmn: cross-contribution compensating multiple standard normalization; CONC: absolute concentration; CUBE: cube root; CV: coefficient of variation; DP: data processing; FA: fatty acid; GC-TOFMS: gas chromatography coupled to a time-of-flight mass spectrometer; GLOG: generalized logarithm; GUI: graphical user interface; IS: internal standard; KEGG: Kyoto Encyclopedia of Genes and Genomes; KP: kynurenine pathway; LC-MS/MS: liquid chromatography platform coupled to tandem mass spectrometer; LN: lupus nephritis; LOG: logarithm; MBPLSDA: multiblock partial least squares discriminant analysis; ML: machine learning; MS: mass spectrometry; nomis: normalization using an optimal selection of multiple internal standards; OPLS-DA: orthogonal partial least squares–discriminant analysis; PC: principal component; PCA: principal component analysis; PLS-DA: partial least squares–discriminant analysis; QC: quality control; repCV: repeated double cross-validation; RF: random forest; RLA: relative log abundance; sqrt: square root; UFA: unsaturated fatty acid; VIP: variable importance in projection.
