# Supplementary material for: Data processing solutions to render metabolomics more quantitative: case studies in food and clinical metabolomics using Metabox 2.0
Source: Gigascience. 2024 Mar 15;13:giae005. doi: 10.1093/gigascience/giae005 (PMC10941642; doi:10.1093/gigascience/giae005)
Supplement: giae005_GIGA-D-23-00257_Original_Submission [file giae005_giga-d-23-00257_original_submission.pdf]

GigaScience

Metabox 2.0: The data processing solution that renders metabolomics more quantitative

--Manuscript Draft--

**Manuscript Number:**	GIGA-D-23-00257
**Full Title:**	Metabox 2.0: The data processing solution that renders metabolomics more quantitative
**Article Type:**	Technical Note
**Funding Information:**	the Program Management Unit for Human Resources & Institutional Development, Research and Innovation (No.630000050069) — Dr Kwanjeera Wanichthanarak<br>the Program Management Unit for Human Resources & Institutional Development, Research and Innovation (630000050069) — Dr Arporn Wangwiwatsin<br>Mahidol University (R016420001) — Dr Sakda Khoomrung<br>the Program Management Unit for Human Resources & Institutional Development, Research and Innovation (B36G660007) — Dr Sakda Khoomrung
**Abstract:**	In classic semi-quantitative metabolomics, metabolite intensities are affected by biological factors and other unwanted variations. A systematic evaluation of the data processing methods is crucial to distinguish adequate processing procedures for a given experimental setup. Current comparative studies are mostly focused on peak area data, but not on absolute concentrations. In this study, we evaluated data processing methods to produce outputs that were most similar to the corresponding absolute quantified data. We examined the data distribution characteristics, fold difference patterns between two metabolites, and sample variance. We used two metabolomic data sets from a retail milk study and a lupus nephritis cohort as test cases. When studying the impact of data normalization, transformation, scaling, and combinations of these methods, we found that the cross-contribution compensating multiple standard normalization (ccmn) method, followed by square root data transformation, was most appropriate for a well-controlled study such as the milk study data set. Regarding the lupus nephritis cohort study, only ccmn normalization could slightly improve the data quality of the noisy cohort. Since the assessment accounted for the resemblance between processed data and the corresponding absolute quantified data, our results denote a helpful guideline for processing metabolomic data sets within a similar context (food and clinical metabolomics). Finally, Metabox 2.0 was introduced, which allows the thorough analysis of metabolomic data, including data processing, biomarker analysis, integrative analysis, and data interpretation. It was successfully used to process and analyze the data in this study. An online web version is available at http://metsysbio.com/metabox.
**Corresponding Author:**	Sakda Khoomrung<br>Mahidol University Faculty of Medicine Siriraj Hospital<br>Bangkok, - None - THAILAND
**Corresponding Author Secondary Information:**	
**Corresponding Author's Institution:**	Mahidol University Faculty of Medicine Siriraj Hospital
**Corresponding Author's Secondary Institution:**	
**First Author:**	Sakda Khoomrung
**First Author Secondary Information:**	
**Order of Authors:**	Sakda Khoomrung

Powered by Editorial Manager® and ProduXion Manager® from Aries Systems Corporation

	Kwanjeera Wanichthanarak
	Ammarin In-on
	Sili Fan
	Oliver Fiehn
	Arporn Wangwiwatsin
**Order of Authors Secondary Information:**	
**Additional Information:**	
**Question**	**Response**
Are you submitting this manuscript to a special series or article collection?	No
**Experimental design and statistics**<br><br>Full details of the experimental design and statistical methods used should be given in the Methods section, as detailed in our Minimum Standards Reporting Checklist. Information essential to interpreting the data presented should be made available in the figure legends.<br><br>Have you included all the information requested in your manuscript?	Yes
**Resources**<br><br>A description of all resources used, including antibodies, cell lines, animals and software tools, with enough information to allow them to be uniquely identified, should be included in the Methods section. Authors are strongly encouraged to cite Research Resource Identifiers (RRIDs) for antibodies, model organisms and tools, where possible.<br><br>Have you included the information requested as detailed in our Minimum Standards Reporting Checklist?	Yes
**Availability of data and materials**<br><br>All datasets and code on which the conclusions of the paper rely must be	Yes

Powered by Editorial Manager® and ProduXion Manager® from Aries Systems Corporation

either included in your submission or deposited in publicly available repositories (where available and ethically appropriate), referencing such data using a unique identifier in the references and in the "Availability of Data and Materials" section of your manuscript.<br><br>Have you have met the above requirement as detailed in our Minimum Standards Reporting Checklist?	

Manuscript

Click here to access/download;Manuscript;GigaScience_Metabox2.docx

Metabox 2.0: The data processing solution that renders metabolomics more quantitative

Kwanjeera Wanichthanarak[1,2], Ammarin In-on[1,2], Sili Fan[3], Oliver Fiehn[4], Arporn Wangwiwatsin[5*], Sakda Khoomrung[1,2,6,7,*]

[1]Siriraj Center of Research Excellence in Metabolomics and Systems Biology (SiCORE-MSB), Faculty of Medicine Siriraj Hospital, Mahidol University, Bangkok 10700, Thailand

[2]Siriraj Metabolomics and Phenomics Center, Faculty of Medicine Siriraj Hospital, Mahidol University, Bangkok 10700, Thailand

[3]University of California Davis Clinical and Translational Science Center, Davis California, USA

[4]West Coast Metabolomics Center, University of California Davis Genome Center, Davis California, USA

[5]Department of Systems Biosciences and Computational Medicine, Faculty of Medicine, Khon Kaen University, Khon Kaen, Thailand

[6]Department of Biochemistry Faculty of Medicine Siriraj Hospital, Mahidol University

[7]Center of Excellence for Innovation in Chemistry (PERCH-CIC), Faculty of Science, Mahidol University, Bangkok, Thailand

*Corresponding author: arpowa@kku.ac.th

: sakda.kho@mahidol.edu

Sakda Khoomrung: https://orcid.org/0000-0001-9461-8597

Arporn Wangwiwatsin: https://orcid.org/0000-0003-4536-4492

Abstract

In classic semi-quantitative metabolomics, metabolite intensities are affected by biological factors and other unwanted variations. A systematic evaluation of the data processing methods is crucial to distinguish adequate processing procedures for a given experimental setup. Current comparative studies are mostly focused on peak area data, but not on absolute concentrations. In this study, we evaluated data processing methods to produce outputs that were most similar to the corresponding absolute quantified data. We examined the data distribution characteristics, fold difference patterns between two metabolites, and sample variance. We used two metabolomic data sets from a retail milk study and a lupus nephritis cohort as test cases. When studying the impact of data normalization, transformation, scaling, and combinations of these methods, we found that the cross-contribution compensating multiple standard normalization (ccmn) method, followed by square root data transformation, was most appropriate for a well-controlled study such as the milk study data set. Regarding the lupus nephritis cohort study, only ccmn normalization could slightly improve the data quality of the noisy cohort. Since the assessment accounted for the resemblance between processed data and the corresponding absolute quantified data, our results denote a helpful guideline for processing metabolomic data sets within a similar context (food and clinical metabolomics). Finally, Metabox 2.0 was introduced, which allows the thorough analysis of metabolomic data, including data processing, biomarker analysis, integrative analysis, and data interpretation. It was successfully used to process and analyze the data in this study. An online web version is available at http://metsysbio.com/metabox.

Keywords: Metabolomics, Quantitative analysis, Semi-quantitative analysis, Data processing, Normalization, Transformation, Scaling, R package

Introduction

Metabolomic analysis is widely accepted as a reliable technology for investigating biochemical activities within a cell or tissue of a living organism, and it has been used to address various questions in biology, drug metabolism, food and nutrition, natural products, and biomedicine [1-3]. Typically, the metabolite level in a sample can be determined quantitatively or semi-quantitatively. Metabolomic quantitative analysis (absolute quantification) aims to ensure the absolute comparability of metabolite concentrations of at least two measurements in a sample obtained at different times or places. On the other hand, semi-quantitative analysis (relative quantification) determines the ratio of metabolite intensity from different samples [4, 5]. Therefore, the absolute concentrations of metabolites represent a benchmark data set that allows an unbiased comparison across different studies. Due to the limited availability of reference standards, most metabolomic studies are conducted in a semi-quantitative manner. However, the inability to compare or correlate the results from different studies remains one of the major limitations of semi-quantitative analysis [6]. This is the primary roadblock to the development of metabolomics research. Therefore, it is essential to encourage the metabolomics community to focus more on using quantitative analyses.

Data processing (DP) plays an important role in semi-quantitative and quantitative analyses; the procedures include imputation, normalization, transformation, scaling, and combinations thereof [7]. To date, numerous DP methods have been proposed in metabolomic studies [7-10]. These methods are mostly based on specific principles and usually have distinct advantages and pitfalls. Therefore, thorough method evaluations are crucial to pinpointing the best-performing process for a given metabolomic study. Many

studies have evaluated and compared DP strategies based on different perspectives. These include the normality structure of the data, changes in global variations, reduction of intra-group distance, results of univariate or multivariate analysis, consistent ranks of putative markers, and classification accuracy [11-15]. Clearly, DP methods are context-dependent and not determined by a sole criterion. However, using information from the classification performance to decide on DP methods is problematic because each method is estimated on the altered data set by itself. Therefore, classifications are prone to overfitting.

Since quantitative analysis is not always applicable in every metabolomic study, choosing a proper choice of DP schemes for polishing the peak areas is crucial to best reflecting the true metabolite levels. This study aimed to employ another strategy to assess the performance of well-known DP methods. The most desirable DP scheme is the one that results in identical statistical results between the processed data and its quantitative companion. The results obtained could be a useful and unbiased reference for DP recommendations. The impact on data distribution, fold difference patterns between two metabolites, and sample variance need to be studied. The DP schemes investigated in this study covered internal standard (IS)-based normalization, transformation, scaling, transformation followed by scaling, and combinations. We used two metabolomic data sets representing different types of data matrices: data relating to a food product with definitive markers and clinical metabolomic data with indistinct variations.

Lastly, we introduced an updated version of the R package Metabox [16] to consolidate a state-of-the-art set of methods for metabolomic analysis from several R packages. Metabox 2.0 enables the in-depth analysis of metabolomic data covering the DP steps, biomarker identification, integrative analysis of multiple data types, and functional interpretation. The software is assembled with ready-to-use R functions that are highly flexible for programming

tasks and have broad application potential. This tool was used for all processing steps and analyses in this study.

Materials and methods

Metabolomic data sets

Quantitative and semi-quantitative metabolomic data were obtained from our recent studies [17, 18]. The first data set (study I) included the nutrient metabolite composition of various retail milk samples purchased in Thailand [18]. In this study, the analysis was focused on 16 fatty acids (FAs) from four milk types: whole bovine milk (n = 13), bovine lactose-free milk (n = 6), soymilk (n = 7), and almond milk (n = 3). Each sample was analyzed in triplicate. The data set contained ten quality control (QC) samples pooled from a mixture of all the milk samples. The FAs were acquired using gas chromatography coupled to a time-of-flight mass spectrometer (GC-TOFMS, Pegasus BT, Leco Corp., St.Joseph, MI).

The second data set (study II) contained information on urine samples collected from Ramathibodi Hospital, Thailand [17]. This was done with approval from the Faculty of Medicine Ethics Committee, Ramathibodi Hospital, Mahidol University, Bangkok, Thailand. The urine samples were acquired from 53 healthy subjects (N) and 63 (LN) patients with lupus nephritis. The metabolites of the kynurenine pathway (KP) were measured using an ultra-performance liquid chromatography platform coupled to a Xevo TQ-S tandem mass spectrometer (LC-MS/MS) and interfaced by an electrospray ionization source (Waters, MA).

The mass spectrometry (MS) data from both studies were pre-processed and quantified as described in previous publications [17, 18]. The concentration of each FA was normalized by its molecular weight (µMol) allowing quantitative comparison across studies. The

concentration of KP metabolites was normalized by the concentration of urinary creatinine. This follows the standard practice of adjusting the concentration of a metabolite to creatinine filtration in nephrotic syndromes [19, 20]. Additionally, missing value imputation was performed on the milk data set before data analysis. A minimum value of each metabolite was imputed to a metabolite with missing values higher than 30 % group-wise. This step was needed because of the true-negative absence of metabolites under specific conditions, as defined by concentrations that were below the detection limit [21]. If applicable, the non-detected metabolites at random (the percentage of non-detected metabolites <30 %) were then imputed by the random forest (RF) method.

Data processing schemes

This study evaluated the DP schemes commonly applied in a general metabolomic workflow [7]. This included normalization, transformation, scaling, and their combinations.

Cross-contribution compensating multiple standard normalization

Cross-contribution compensating multiple standard normalization (ccmn) is an IS-based normalization in which metabolite abundances are estimated proportionately to a known IS quantity [22]. Additionally, it considers systematic error and study factors as independent sources of variation on ISs; important information is unaffected by normalization [22]. In contrast to a closely related method, such as normalization using an optimal selection of multiple internal standards (nomis), this method removes unwanted systematic variation based on the variability of single or multiple ISs [23].

Data transformation

Transformation is a process to reduce data skewness, fix heteroscedasticity, and turn multiplicative metabolite relationships into additive relationships [24]. Six transformation methods were assessed in this study, including cube root (cube), logarithm (log2 and log10), generalized log (glog2 and glog10), and square root (sqrt) transformations (Table S1). Transformations can reduce the differences between large and small values, whereby large values are scaled down much more than small values [24]. These transformations lead to a depletion in right skewness, which is an observed characteristic of omics data such as metabolomic and transcriptomic data [25]. The cube and glog transformations accept zero and negative values, whereas the sqrt transformation can only manage zero values. In contrast, log transformations can only handle non-zero and non-negative values. The glog transforms the data to which a specific parameter of each data set applies [26]. Additionally, it focuses on stabilizing data variance, i.e., keeping the variance constant and independent from the mean [26, 27].

Data scaling

Scaling reduces the fold difference between metabolite concentrations based on scaling factors [24]. This is unlike the pseudo-scaling effect of transformations. Here, a scaling factor is determined explicitly for a particular metabolite. This study compared six scaling methods: auto, level, pareto, power, range, and vast scaling (Table S1). The auto, pareto, range, and vast scaling estimates are scaling factors that are based on data dispersion. In contrast, level scaling is based on the mean value [24]. Power scaling performs an average subtraction in combination with the sqrt transformation [28].

Analysis workflow

Different DP schemes were investigated using study I [18] and study II [17] data sets that comprised both quantitative (the absolute levels) and semi-quantitative (the peak areas of metabolites) results. Key DP schemes were performed to evaluate their effect on the peak area data, which included (A) no processing (raw area), (B) transformation, (C) scaling, (D) transformation followed by scaling, (E) IS-based normalization by the ccmn method, and (F) ccmn normalization combined with transformation, -combined with scaling, or a combination of both (Figure 1). In total, 97 processed data sets were analyzed, and unprocessed data was considered. We evaluated the influence of the DP methods and their combinations on different aspects, including normality, skewness, coefficient of variation (CV), the trend of fold differences, sample heterogeneity, and multivariate analysis outputs.

The normality test and measures of skewness were computed by the Shapiro-Wilk normality test [29] and the skewness function of the e1071 package [30], respectively. A p-value > 0.05 indicated a normal distribution, and the symmetric skewness was from −0.5 to 0.5. The CV for a metabolite was the ratio of the standard deviation to the mean within a group. The fold and directional differences of a metabolite from a reference were calculated. Since most DP methods strongly affect highly abundant metabolites, the metabolite with the highest level was used as a reference point. An across-group relative log abundance (RLA) plot was applied to explore the grouping structure, outliers, and variation within each group. Each metabolite was standardized by subtracting the median from across all groups [31]. A principal component analysis (PCA) was performed to visualize the overall variations of the data regarding the biology of interest.

Moreover, the effects of various DP methods on the partial least squares-discriminant analysis (PLS-DA) in comparison to the absolute concentration (CONC) data were examined. The variable importance in projection (VIP) of a metabolite indicates its degree of contribution

to explain variance in the PLS model [32]. The similarity between the resulting VIPs from the CONC data, raw area data, and processed data was computed. The similarity between the two approaches, x and y, was calculated using Euclidean distance as the following equation (1):

$$Similarity\ (x, y) = 1 - \sqrt{\sum_{i}^{n} (x_i - y_i)^2} \quad (1)$$

For the method x, we denoted the VIP score of the ith metabolite as x_i; where $i = 1, 2, \ldots, the\ number\ of\ metabolites\ (n)$. The same definition was applied to the method y. The hierarchical clustering of the VIP scores was performed to infer the grouping of the DP schemes. The ComplexHeatmap R package [33] was used for clustering analysis. All DP tasks, PCA, PLS-DA, and plot generation, were performed using the R package Metabox 2.0 DP and analysis pipeline.

Implementation of Metabox 2.0

Metabox 2.0 is a standard R package developed from R version 4.2.0, providing a substantial update to the first Metabox version [16]. A collection of R packages for metabolomic analysis is extensively applied (Table S1). We enclosed the sequences of DP and analysis tasks in R functions. The graphical user interface (GUI) is implemented with the R package Shiny [34]. An overview of the analysis pipelines is illustrated in Figure S1.

Data processing and analysis pipeline

This analysis pipeline supports the DP step and consecutive data analyses, including essential statistical analyses and biomarker discovery (Figure S1A). The DP module includes

all major metabolomic DP tasks, starting with missing value imputation, normalization, transformation, and data scaling. A collection of commonly used methods is integrated into the Metabox 2.0 (Table S1). All three main types of imputation methods are provided, including single value, local similarity, and global structure approaches [21]. The normalization module covers IS-, QC sample- and data-based approaches, which consistently aim to accommodate unwanted errors and crucial biological variation [8, 31]. IS-based and QC sample-based normalization relies on spike-in ISs and the intensity of QC samples, respectively [8]. Meanwhile, the data-driven normalization summarizes a sample-specific factor for the adjustment [31]. The transformation methods for decreasing right skewness and scaling methods based on either data dispersion or mean value are included. When performing both data transformations and scaling, the differences in magnitude between large and small metabolite values are adjusted, so that those metabolites are fairly comparable in statistics. In total, there are ten imputations, three IS-based normalizations, two QC sample-based normalizations, 12 data-driven normalizations, six transformations, and six scaling methods.

The statistical analysis module comes with a collection of prominent univariate analysis methods. These are well-known statistical hypothesis testing methods and post hoc tests covering parametric and non-parametric tests, a pairwise correlation analysis, and the linear mixed modeling from the lmm2met package [7] (Table S2). For multivariate analysis, both unsupervised and supervised multivariate analyses are included, incorporating the PCA, PLS-DA, and orthogonal PLS-DA (OPLS-DA) implemented from the ropls package [35].

The biomarker analysis module supports regression and classification analyses using the PLS or random forest (RF) approach. We incorporate recursive variable elimination within a repeated double cross-validation (repCV) approach from the MUVR package [36] to identify

informative metabolites. The algorithm addresses prediction accuracy, model overfitting, and optimally relevant metabolites.

Data integration pipeline

Metabox 2.0 supports the joint analysis of multiple data types, such as omics and other phenotypic data (Figure S1B). The multi-block PLS-DA (MBPLSDA) pipeline from the mbpls package [37] is assimilated into the integrative analysis module, focusing on the multivariate modeling of concatenated data blocks by considering the specific data structure of each block. This method allows the estimation of both variable and block importance.

Data interpretation pipeline

This pipeline includes well-established methods for functional interpretation in the context of metabolic pathways and chemical classes (Figure S1C). The set enrichment analysis and overrepresentation analysis can be performed with a comprehensive collection of methods from the piano package [38], as implemented in Metabox 1.0 [16]. Moreover, integrated pathway overrepresentation analysis uses Fisher's method to combine p-value outputs from the hypergeometric test. The KEGG database [39] is utilized for pathway information, whereas chemical classes of metabolites are based on the HMDB chemical taxonomy [40].

Results

The effects of data processing on the semi-quantified fatty acids in milk samples

All 16 FAs were quantified in whole and lactose-free bovine milk (Figure S2). However, the FAs, including C10:0 and C14:1, were not detected in the plant-based milk products (soymilk

and almond milk). C6:0, C14:0, C22:0, and C24:0 were not present in almond milk. C8:0, C12:0, C15:0, C16:1, and C17:0 were detectable in soymilk, but absent in almond milk. These metabolites were imputed by their minimum value prior to the DP step. As reported by Jariyasopit *et al.*, C16:0 (palmitic acid) and two unsaturated FAs (UFAs) [C18:1 cis-9 (oleic acid) and C18:2n-6 or C18:2 (linoleic acid)] were at their highest concentrations (mg/L) in bovine milk, almond milk, and soymilk, respectively [18]. The amount of C18:1 cis9 in almond milk was remarkedly high (14230.98 ± 4057.15 µMol).

Effects on data properties

Initially, we explored the effects of each DP scheme on the basic properties of the milk data set. For each metabolite, we considered the number of normally distributed, positively skewed, and negatively skewed metabolites and the CV in each sample group (Table S3). All FAs in the QC samples were normally and symmetrically distributed for the CONC data. Most FAs in bovine milk and soymilk were of the right skewness (i.e., a few FAs were highly abundant), while the FAs in bovine lactose-free milk were mostly normally distributed. The largest and smallest CVs were noticed in soymilk and almond milk, respectively. Similar aspects were observed in the peak area data. The PCA plot showed that the ccmn method was the main factor contributing to separation among the DP schemes, with and without this normalization (Figure 2A). The number of normally distributed metabolites in the almond milk dramatically increased after normalization, and most FAs became slightly negatively skewed. The basic properties of the quantified and raw area data were closer to those of the unnormalized data sets. Each DP scheme resulted in an apparent cluster. The transformations increased the number of normally distributed metabolites in bovine milk. The area data processed by the scaling scheme was separated from those altered by the transform+scale

and the transformation, with and without normalization. All scaling methods returned similar data properties, with power scaling being slightly different. After power scaling, the area data had the same data properties as those after sqrt+auto, level, pareto, range, or vast scaling (Figure 2B). All glog- and log-based transformations produced similar data properties. However, the cube, sqrt, and log-based transformations were attributed to slightly different data properties, particularly the distribution of metabolites in bovine milk. As such, we noticed a separation among the cube, sqrt, and both log-based transformation schemes.

Effects on multivariate analysis

The PLS-DA was performed on 97 processed data sets, the absolute FA concentration and peak area data sets. Cluster analysis of the resulting VIPs revealed grouping of the CONC data, raw area data, ccmn, ccmn+pareto, ccmn+power, and ccmn+sqrt processed data (Figure S3). These DP schemes formed a separate branch from the DP, involving log-based transformations, auto, range, or vast scaling. The VIP scores of the data that had undergone ccmn+power and ccmn+sqrt processing were identical. Moreover, they were similar to the VIPs from the quantified data (Figure 3A and Table S4, similarity = 80.32%). The VIPs from the unprocessed area data were approximately 20% different from the original CONC data. In contrast, when using the glog-, log-, auto-, range-, and vast-based DP methods, the VIP similarity was reduced to below 40%. Any DP counting in the vast method led to a low similarity of approximately 10% or less. The ccmn+cube+vast was the least alike (similarity = 4.16%).

C18:1 cis9 and C18:2n-6, the important plant UFAs [41], were the discriminant metabolites (VIP $\geq$ 1.5) obtained from the PLS-DA on the CONC and raw area data (Figure S3). Moreover, both UFAs were identified from the data processed by sqrt or cube transformation, level,

pareto, or power scaling. VIP scores increased slightly when ccmn normalization was applied together with sqrt, cube, pareto, or power. Combining these transformation and scaling methods led to lower VIPs, particularly the VIP of C18:2. C16:0 was an additional discriminant for the plant UFAs in the ccmn-normalized data. The DP schemes involving vast scaling failed to identify C18:1 cis9 and C18:2n-6, as their VIPs were less than 1.0. The number of FAs with VIP >1.0 was increased by glog- or log-based transformation, yet it diminished the importance of the plant UFAs in the PLS model.

The PCA plots showed a clear partitioning of the almond milk, soymilk, and bovine milk, except for the ccmn+cube+vast processed data (Figure 3B). This DP task mitigated the variance between soymilk and almond milk. The variability explained by the 1st and 2nd principal components (PCs) was 99.32%, 97.87%, 92.35%, and 84.05% for the CONC, raw area, ccmn+sqrt, and ccmn+cube+vast processed data, respectively. Though the observed variation of ccmn+sqrt was less than that of the raw area data, the original structure of variation was more preserved with ccmn+sqrt processing. The milk data set possessed intragroup variability, which was still visible after processing with ccmn+sqrt (Figure S4A). However, this within-group variation was inflated, and the plant-based samples displayed a right-skewed distribution after applying ccmn+cube+vast.

For the CONC and peak area data, the mean level of the CV (mCV) in each milk type was as follows: soymilk > bovine milk > bovine lactose-free milk > QC sample > almond milk (Figure S4B). The ccmn+sqrt method could lessen metabolite dispersion, in contrast to the ccmn+cube+vast. In sequential order, it enlarged the mCV of the QC, bovine lactose-free milk, bovine milk, soymilk, and almond milk samples. In addition, the trend of the fold differences between C18:1 cis-9 and the other FAs in all milk types was substantially altered by ccmn+cube+vast (Figure S4C). Specifically, the C18:1 cis-9 abundance became less than the

FAs C8:0, C10:0, C14:1, C15:0, C16:1, and C17:0 in both bovine milk types, as opposed to the original CONC data. The amount of C18:1 cis-9 was higher than that of C16:0 in the bovine milk samples and lower in the almond milk. In the case of the ccmn+sqrt method, overall fold differences were maintained and comparable to the original CONC data.

Effects on multivariate analysis in the absence of highly abundant metabolites

The absolute amounts of C18:1 cis-9 and C18:2 were relatively high compared to those of the other FAs (Figure S2). C18:1 cis-9 and C18:2 were the main discriminants between almond milk, soymilk, and plant-based and bovine milk in the PLS-DA (Figure 3). We continued our evaluation of the milk data set, excluding the major metabolites C18:1 cis-9 and C18:2.

From VIP clustering, the CONC, raw area, ccmn-normalized, and pareto-scaled data were grouped and formed the closest linkage to a cluster containing either the cube, sqrt, or power processing alone or in combination with ccmn normalization (Figure S5). Elements in this group included ccmn+pareto, with/without cube or sqrt transformation, and ccmn+sqrt+power. These DP schemes formed a distant branch from the DP tasks embracing auto, level, range, vast scaling, or log-based transformations. In particular, the percentage of VIP similarity was less than 40%. The VIPs from glog2+vast processed data were the most dissimilar (Table S5, similarity = 31.54%), and only C24:0 was the discriminative metabolite from this method (Figure S6A). The most similar VIP scores were the VIP scores of the peak area (similarity = 83.28%). Whereas, the VIPs from the data processed by the ccmn+power or ccmn+sqrt processing were slightly less identical (similarity = 80.35%). The discriminant metabolites C16:0 and C4:0 were commonly observed from the CONC, area, and ccmn+sqrt processed data. C14:0 was the additional discriminant for the CONC and ccmn+sqrt processed data, while C18:0 was identified for the peak area data.

The apparent separation between plant-based and bovine milk was observed from the PCA plots, except for the area and glog2+vast processed data (Figure S6B). The data structure of the CONC data was more preserved in the ccmn+sqrt processed data set. Meanwhile, the PCA plots of the area data with (Figure 3B) and without the plant UFAs (Figure S6B) were comparable. The cumulative variance explained by PC1 and PC2 for the CONC, area, ccmn+sqrt, and glog2+vast processed data was 97.43%, 99.47%, 97.40%, and 83.56%, respectively. Though, we noticed the fine cluster of plant and bovine milk types by the glog2+vast method, the high variance was introduced within the QC and bovine milk samples. Accordingly, we detected a substantial impact on the data distribution, outliers (Figure S6C) and metabolite deviation from its mean (Figure S6D). In contrast to ccmn+sqrt, fold difference tendencies between C16:0 and the other FAs, in all milk types were markedly influenced after applying glog2+vast (Figure S6E).

Data processing effects on the semi-quantified metabolites in urine samples

Similar aspects were performed to evaluate and compare the effects of the DP methods on eight KP metabolites in urine samples (study II). For CONC and raw area data, almost all metabolites were positively skewed, and the mCV of the LN samples was slightly higher than that of the normal samples (Table S6). The basic properties of the quantified and ccmn-normalized data were closer than those of the other data sets. However, the combined ccmn with transformation, or transform+scale, was not the major factor influencing the separation, as observed in the study I (Figure S7). When applying the transformation, scaling, or transform+scale schemes, the DP effect on the urine data properties appeared consistent with the milk sample data.

We observed the clustering of VIPs from the sqrt, pareto, power, ccmn+cube, ccmn+sqrt, ccmn+pareto, ccmn+power, and area data (Figure S8). They formed a distinct branch from the DP schemes, including glog, log, auto, level, range, and vast. Meanwhile, the VIPs from the CONC and the ccmn-normalized data were distinguished from those of the other methods. The VIP scores from the unprocessed peak areas were 55.53%, identical to the CONC data (Table S7). The VIP scores of the ccmn-normalized data were the closest to those of the quantified data (Figure S9A, similarity = 62.36%). In contrast, the ccmn+level DP led to the least similar VIPs (similarity = 41.62%). Tryptophan was the discriminant metabolite (VIP ≥ 1.2) observed in the CONC data, raw areas, and ccmn processed data. Kynurenic acid was identified as an important metabolite in both the CONC and the ccmn-normalized data. Picolinic acid (VIP >1.6) was the discriminative metabolite observed in the data sets that applied the DP glog, log, auto, level, range, or vast methods (Figure S8). From the CONC data, this metabolite possessed a low VIP weight (VIP = 0.03). In contrast, 3−hydroxykynurenine was only reported from the CONC data (VIP = 1.52) and was absent in the other data sets.

The urine samples from healthy subjects and LN patients mainly overlapped (Figure S9B) and showed high within-group variation (Figure S9C). However, the ccmn method improved the explained variance in the 1st PC (PC1 = 81.02%) compared to the raw area data (PC1 = 74.78%). In contrast, when using the ccmn+level method, we observed large influences on the sample distribution, metabolite variation, and fold differences between tryptophan and the other metabolites (Figure S9C–E). When using this method, the PC1 and cumulative variance were 41.77% and 61.56%, respectively.

IS-based normalization performance is dependent on the type of biological factors

Two commonly used IS-based normalization methods, ccmn and nomis, were further evaluated using milk and urine data sets. The milk data set has a definitive biological effect, whereas the urine samples comprise many unknown individual variations. In this assent, the informative variation of milk samples was retained by the ccmn method, unlike the nomis method (Figure 4A). The variances explained by the 1st and 2nd PCs were 95.97% and 92.20% for the ccmn- and nomis-normalized data, respectively. The 1st PC presented the differences between almond milk and soymilk for the ccmn-processed data. This aspect was invisible in the nomis-normalized data. In the case of the urine data set, we observed that the ccmn method performed similarly to the nomis method (Figure 4B). The groups of healthy and LN samples were slightly separated. Within the LN group, variation was slightly reduced after normalization. Subject-specific variations and the presence of outliers are common in clinical metabolomics. Moreover, the ccmn method only assumes linear relationships between measured metabolites and experimental factors, which is not always the case in metabolomics [22]. Therefore, the metabolite and IS interferences in the urine matrix may not be thoroughly corrected by the ccmn method.

Metabox 2.0: enhancing metabolomic data analysis, integration, and interpretation

Metabox 2.0 is executed as a standard R package. The current version has undergone significant redesign and updates since Metabox 1.0 [16], highlighting the analysis of metabolomic data from DP steps to biomarker identification and allowing the joint analysis of multiple data types, such as LC- and GC-MS metabolomes, metabolomic and transcriptomic data sets, or metabolomic and clinical data. Three analysis pipelines are organized as separate modules (Figure 5 and Figure S1). A series of scripts for a particular task is encoded in a ready-to-use R function, allowing the implementation of customized workflows. The key features of

this version include: 1) a collection of state-of-the-art methods for end-to-end metabolomic data analysis; 2) normalization methods for cohort- and laboratory-scale metabolomic studies; 3) univariate analysis for one or multiple factors; 4) multivariate modeling for both classification and regression; 5) machine learning (ML)-based biomarker analysis with minimizing model overfitting and false-positive rates; 6) cross-domain data integration; 7) data interpretation in the context of metabolic pathways and chemical classes; 8) various kinds of plots for data exploration; and 9) an intuitive GUI for bench biologists (Figure 5). This GUI version supports typical analysis and allows broader usability as a hosted web application on the server. The integrative exploration of multi-omic levels in biological networks is excluded in this version because it requires the pre-installation of a specific graph database system.

For metabolomic analysis, this tool serves as an alternative to closely related software such as MetaboAnalyst [42], NOREVA [28], and OUKS [43]. Metabox 2.0 covers more DP methods, and the tool for integrative analysis of omic and non-omic data is equipped. Metabox 2.0 is an open-source R package freely accessible at https://github.com/kwanjeeraw/metabox2 under the GPL-3 license. Furthermore, an online web version is publicly available at http://metsysbio.com/metabox.

Discussion

Quantitative analysis is ideal in metabolomics research; however, the absolute quantification of all metabolites can be challenging. A good DP scheme for raw data processing is essential to resemble semi-quantitative to quantitative data. In this study, we characterized and compared the semi-quantified metabolites after different DP treatments

to their quantified counterparts. We covered three common scenarios in metabolomics, including a data set with apparent markers, a data set with a known biological effect, and a data set with obscure variations.

The results consistently indicated that normalization and transformation had an impact on the data distribution, skewness, and CV, while scaling only influenced the CV of the data. Apart from one exception, power scaling behaved like the sqrt+scale scheme. This is because the method relies on the square root of metabolite intensity along with a mean subtraction [28]. However, these data properties could not directly reflect the final results of statistical analyses, e.g., how many significant metabolites are identified or how much class discrimination is improved by a specific DP method. An understanding of data distribution could guide the choice of subsequent statistical analysis. The change in the CV is an indicator of DP performance in reducing group variation [44].

The use of milk and urine sample data sets represented two sides of the story. As a food product, the milk samples were produced in well-controlled environments, while the urine study data was not. Even though there were strict inclusion and exclusion criteria in the study cohort. Inter-individual variations, e.g., dietary, genetic, and demographic background, were the key unwanted variations in clinical metabolomics [7]. Accordingly, we observed that the PLS-DA result from the milk area data resembled its CONC data more than that of the urine study data. The raw peak areas, excluding the two plant UFAs, resulted in the most similar result to the CONC data without any processing task. Due to the numerous unknown sample matrices, the ccmn method performed similarly to nomis normalization in the urine data set. Overall, ccmn normalization improved the quality of semi-quantitative data in every case in our study and is our recommendation. Furthermore, the ccmn method can segregate IS

interference because of the correlation with the factors under study [22, 31]. As such, it can avoid the risk of losing informative variations in the nomis.

The normalization process aims to remove systematic errors and unrelated biological variations (if applicable). However, it cannot scale for magnitude differences among metabolites. When considering the milk sample data set, which included plant UFAs, the ccmn+sqrt method was suggested. This was because the data set had a distinct biological factor, and the sqrt transformation had the least effect on the variance structure compared to the other transformation methods. In descending order, the effect size ranged from the log family, cube transformation, and sqrt method. The performance of the scaling methods, power, and pareto, was relatively comparable and had a smaller effect on the data variance than the other methods. The vast-scaled milk and level-scaled urine produced the most divergence among VIP results from the CONC data. This is due to the fact that vast scaling is suitable for data sets with small induced fluctuations [11, 24], which is not the case in this study. The FAs with a large variation were considered less important, while a low-deviated metabolite became more significant after vast scaling. In contrast to level scaling, this approach is suggested for a study that involves large relative responses of a biological factor [11, 24]. This method failed when using the urine sample data because the signal-to-noise ratio was low.

In summary, our study showed that the ccmn or the ccmn+sqrt method could attribute the most similar PLS-DA results between the quantified and the semi-quantified equivalents. Given that the ccmn outperformed the other methods in this study, various research projects are underway to improve the normalization strategy, i.e., the use of quality control metabolites [31], quality control samples [8], subject-specific characteristics [7], and nonlinear modeling [8, 9, 22]. The transform+scale and normalize+transform+scale schemes

could not enhance the quality of the semi-quantitative data in many cases. This may be because of an excessive alteration in the data variance by both transformation and scaling.

Many studies have compared different strategies for metabolomics data processing [11-15]. However, this is the first study to use quantitative data as a reference point for DP evaluation. Our findings are based on the closest representation of genuine metabolite patterns, which could be a valuable guide for the DP procedure. In practice, the CONC data is not always available for comparison. Therefore, method evaluations, such as those performed in this and the other studies, are highly advised prior to any statistical analysis, e.g., the changes in CV, the fold difference trends, the unsupervised patterns of the PCA score plot, and the clustering of multivariate analysis outputs. Most bioinformatics tools, such as Metabox 2.0, MetaboAnalyst [42], NOREVA [28], and OUKS [43], provide various features to facilitate DP evaluations and comparisons.

Conclusion

This study reported the effects of DP schemes on data properties and variance structure. Normalization and transformations altered the data normality, skewness, and CV, whereas data scaling only changed the CV. The PCA and the final results of PLS-DA were compared between the absolute abundances and the processed peak areas. This was to observe the outcome of different DP schemes on the data variance. The ccmn+sqrt outperformed for the data sets with apparent markers and a known biological effect. Furthermore, the raw area may be used if the samples are from a well-defined experiment and have a known matrix effect. Though, the resulting VIPs from the raw peak of urine metabolites were slightly over 50% identical to the absolute levels, the IS-based normalization, such as ccmn, was the best

option to improve this clinical metabolomics data. Choosing a strong DP method, e.g., log transformation, auto, range, vast, and level scaling, needs careful consideration. These methods have less tolerance for outliers and tend to pick up noise. Our study used another aspect of the DP criteria. The best DP choice allowed the semi-quantitative data to mimic the quantitative data. Additionally, we discussed the bioinformatics toolbox, Metabox 2.0, which contains significant updates on the DP tasks, biomarker identification, and integrative analysis. This was an underlying tool for all analyses in this study.

Availability of Source Code

Project name: Metabox 2.0

Project home page: https://metsysbio.com/metabox/index.html

Operating system(s): Platform independent

Programming language: R

Other requirements: None

License: GNU General Public License (v3)

Data Availability

R code used for this article and intermediary files are available from GigaDB (ref TBA). The metabox2 package and its full source code are available from GitHub, https://github.com/kwanjeeraw/metabox2. The data sets used in this article are available in the Mendeley Data repository, study I: https://doi.org/10.17632/6v5gkjg9c6.1, and study II: https://doi.org/10.17632/srcz7scz67.2.

Competing interests

The authors declare that there is no conflict of interest.

Funding

KW, AW, and SK acknowledge support from the Program Management Unit for Human Resources & Institutional Development, Research and Innovation, through Khon Kaen University Cholangiocarcinoma Research Institute (No. 630000050069) for the development of Metabox 2.0. The project was supported by Mahidol University, Grant No. (IO) R016420001 (to SK). The running server for the online version is supported by the Program Management Unit for Human Resources & Institutional Development, Research and Innovation, Grant No. B36G660007 (to SK). This project is partially supported by the Research Excellence Development (RED) program, Faculty of Medicine Siriraj Hospital, Mahidol University.

Acknowledgments

KW thanks Dr. Narumol Jariyasopit for technical advice regarding the milk metabolomic data sets.

Author's contributions

KW, AI, and SK conceived and designed the study. KW and AI developed the software. KW, AI, and SK performed data analysis. OF and SK supervised the analyses, gave scientific discussion, and assisted in manuscript preparation. AW and SK provided the resources for the study. KW

and SK wrote the original draft. KW, AI, SF, OF, AW, and SK edited the manuscript. All authors reviewed the manuscript and approved the submitted version.

Reference

1. Kim, S., et al., *Food metabolomics: from farm to human.* Curr Opin Biotechnol, 2016. **37**: p. 16-23.
2. Khoomrung, S., et al., *Metabolomics and Integrative Omics for the Development of Thai Traditional Medicine.* Front Pharmacol, 2017. **8**: p. 474.
3. Wishart, D.S., *Metabolomics for Investigating Physiological and Pathophysiological Processes.* Physiol Rev, 2019. **99**(4): p. 1819-1875.
4. Tebani, A., C. Afonso, and S. Bekri, *Advances in metabolome information retrieval: turning chemistry into biology. Part I: analytical chemistry of the metabolome.* Journal of Inherited Metabolic Disease, 2018. **41**(3): p. 379-391.
5. Noack, S. and W. Wiechert, *Quantitative metabolomics: a phantom?* Trends in Biotechnology, 2014. **32**(5): p. 238-244.
6. Yang, Q., et al., *Metabolomics biotechnology, applications, and future trends: a systematic review.* RSC Adv, 2019. **9**(64): p. 37245-37257.
7. Wanichthanarak, K., et al., *Accounting for biological variation with linear mixed-effects modelling improves the quality of clinical metabolomics data.* Comput Struct Biotechnol J, 2019. **17**: p. 611-618.
8. Fan, S., et al., *Systematic Error Removal Using Random Forest for Normalizing Large-Scale Untargeted Lipidomics Data.* Anal Chem, 2019. **91**(5): p. 3590-3596.
9. Rong, Z., et al., *NormAE: Deep Adversarial Learning Model to Remove Batch Effects in Liquid Chromatography Mass Spectrometry-Based Metabolomics Data.* Anal Chem, 2020. **92**(7): p. 5082-5090.
10. Yu, H., P. Sang, and T. Huan, *Adaptive Box-Cox Transformation: A Highly Flexible Feature-Specific Data Transformation to Improve Metabolomic Data Normality for Better Statistical Analysis.* Anal Chem, 2022. **94**(23): p. 8267-8276.
11. Gromski, P.S., et al., *The influence of scaling metabolomics data on model classification accuracy.* Metabolomics, 2015. **11**(3): p. 684-695.
12. Di Guida, R., et al., *Non-targeted UHPLC-MS metabolomic data processing methods: a comparative investigation of normalisation, missing value imputation, transformation and scaling.* Metabolomics, 2016. **12**: p. 93.
13. Cuevas-Delgado, P., et al., *Data-dependent normalization strategies for untargeted metabolomics-a case study.* Anal Bioanal Chem, 2020. **412**(24): p. 6391-6405.
14. Wu, Y. and L. Li, *Sample normalization methods in quantitative metabolomics.* J Chromatogr A, 2016. **1430**: p. 80-95.
15. Chen, J., et al., *Influences of Normalization Method on Biomarker Discovery in Gas Chromatography-Mass Spectrometry-Based Untargeted Metabolomics: What Should Be Considered?* Anal Chem, 2017. **89**(10): p. 5342-5348.
16. Wanichthanarak, K., et al., *Metabox: A Toolbox for Metabolomic Data Analysis, Interpretation and Integrative Exploration.* PLoS One, 2017. **12**(1): p. e0171046.
17. Anekthanakul, K., et al., *Predicting lupus membranous nephritis using reduced picolinic acid to tryptophan ratio as a urinary biomarker.* iScience, 2021. **24**(11): p. 103355.

18. Jariyasopit, N., et al., *Quantitative analysis of nutrient metabolite compositions of retail cow's milk and milk alternatives in Thailand using GC-MS.* Journal of Food Composition and Analysis, 2021. **97**: p. 103785.
19. Goldstein, S.L., *Urinary kidney injury biomarkers and urine creatinine normalization: a false premise or not?* Kidney Int, 2010. **78**(5): p. 433-5.
20. Waikar, S.S., V.S. Sabbisetti, and J.V. Bonventre, *Normalization of urinary biomarkers to creatinine during changes in glomerular filtration rate.* Kidney Int, 2010. **78**(5): p. 486-94.
21. Kokla, M., et al., *Random forest-based imputation outperforms other methods for imputing LC-MS metabolomics data: a comparative study.* BMC Bioinformatics, 2019. **20**(1): p. 492.
22. Redestig, H., et al., *Compensation for systematic cross-contribution improves normalization of mass spectrometry based metabolomics data.* Anal Chem, 2009. **81**(19): p. 7974-80.
23. Sysi-Aho, M., et al., *Normalization method for metabolomics data using optimal selection of multiple internal standards.* BMC Bioinformatics, 2007. **8**(1): p. 93.
24. van den Berg, R.A., et al., *Centering, scaling, and transformations: improving the biological information content of metabolomics data.* BMC Genomics, 2006. **7**: p. 142.
25. Li, B., et al., *Performance Evaluation and Online Realization of Data-driven Normalization Methods Used in LC/MS based Untargeted Metabolomics Analysis.* Sci Rep, 2016. **6**: p. 38881.
26. Parsons, H.M., et al., *Improved classification accuracy in 1- and 2-dimensional NMR metabolomics data using the variance stabilising generalised logarithm transformation.* BMC Bioinformatics, 2007. **8**(1): p. 234.
27. Durbin, B.P., et al., *A variance-stabilizing transformation for gene-expression microarray data.* Bioinformatics, 2002. **18 Suppl 1**: p. S105-10.
28. Yang, Q., et al., *NOREVA: enhanced normalization and evaluation of time-course and multi-class metabolomic data.* Nucleic Acids Res, 2020. **48**(W1): p. W436-W448.
29. Royston, J.P., *Algorithm AS 181: The W Test for Normality.* Journal of the Royal Statistical Society. Series C (Applied Statistics), 1982. **31**(2): p. 176-180.
30. Meyer, D., et al., *Misc Functions of the Department of Statistics, ProbabilityTheory Group (Formerly: E1071), TU Wien.* 2015.
31. De Livera, A.M., et al., *Statistical methods for handling unwanted variation in metabolomics data.* Anal Chem, 2015. **87**(7): p. 3606-15.
32. Farrés, M., et al., *Comparison of the variable importance in projection (VIP) and of the selectivity ratio (SR) methods for variable selection and interpretation.* Journal of Chemometrics, 2015. **29**(10): p. 528-536.
33. Gu, Z., *Complex heatmap visualization.* iMeta, 2022. **1**(3): p. e43.
34. Chang, W., et al., *shiny: Web Application Framework for R*. 2023.
35. Thévenot, E.A., et al., *Analysis of the Human Adult Urinary Metabolome Variations with Age, Body Mass Index, and Gender by Implementing a Comprehensive Workflow for Univariate and OPLS Statistical Analyses.* Journal of Proteome Research, 2015. **14**(8): p. 3322-3335.
36. Shi, L., et al., *Variable selection and validation in multivariate modelling.* Bioinformatics, 2019. **35**(6): p. 972-980.

37. Brandolini-Bunlon, M., et al., *Multi-block PLS discriminant analysis for the joint analysis of metabolomic and epidemiological data.* Metabolomics, 2019. **15**(10): p. 134.
38. Varemo, L., J. Nielsen, and I. Nookaew, *Enriching the gene set analysis of genome-wide data by incorporating directionality of gene expression and combining statistical hypotheses and methods.* Nucleic Acids Res, 2013. **41**(8): p. 4378-91.
39. Kanehisa, M., et al., *KEGG for integration and interpretation of large-scale molecular data sets.* Nucleic Acids Res, 2012. **40**(Database issue): p. D109-14.
40. Wishart, D.S., et al., *HMDB 5.0: the Human Metabolome Database for 2022.* Nucleic Acids Res, 2022. **50**(D1): p. D622-D631.
41. He, M., et al., *Plant Unsaturated Fatty Acids: Biosynthesis and Regulation.* Front Plant Sci, 2020. **11**: p. 390.
42. Pang, Z., et al., *MetaboAnalyst 5.0: narrowing the gap between raw spectra and functional insights.* Nucleic Acids Res, 2021. **49**(W1): p. W388-W396.
43. Plyushchenko, I.V., et al., *Omics Untargeted Key Script: R-Based Software Toolbox for Untargeted Metabolomics with Bladder Cancer Biomarkers Discovery Case Study.* J Proteome Res, 2022. **21**(3): p. 833-847.
44. Li, B., et al., *NOREVA: normalization and evaluation of MS-based metabolomics data.* Nucleic Acids Res, 2017. **45**(W1): p. W162-W170.

Click here to access/download;Figure;List_Of_Figures_20230626.docx

Figures

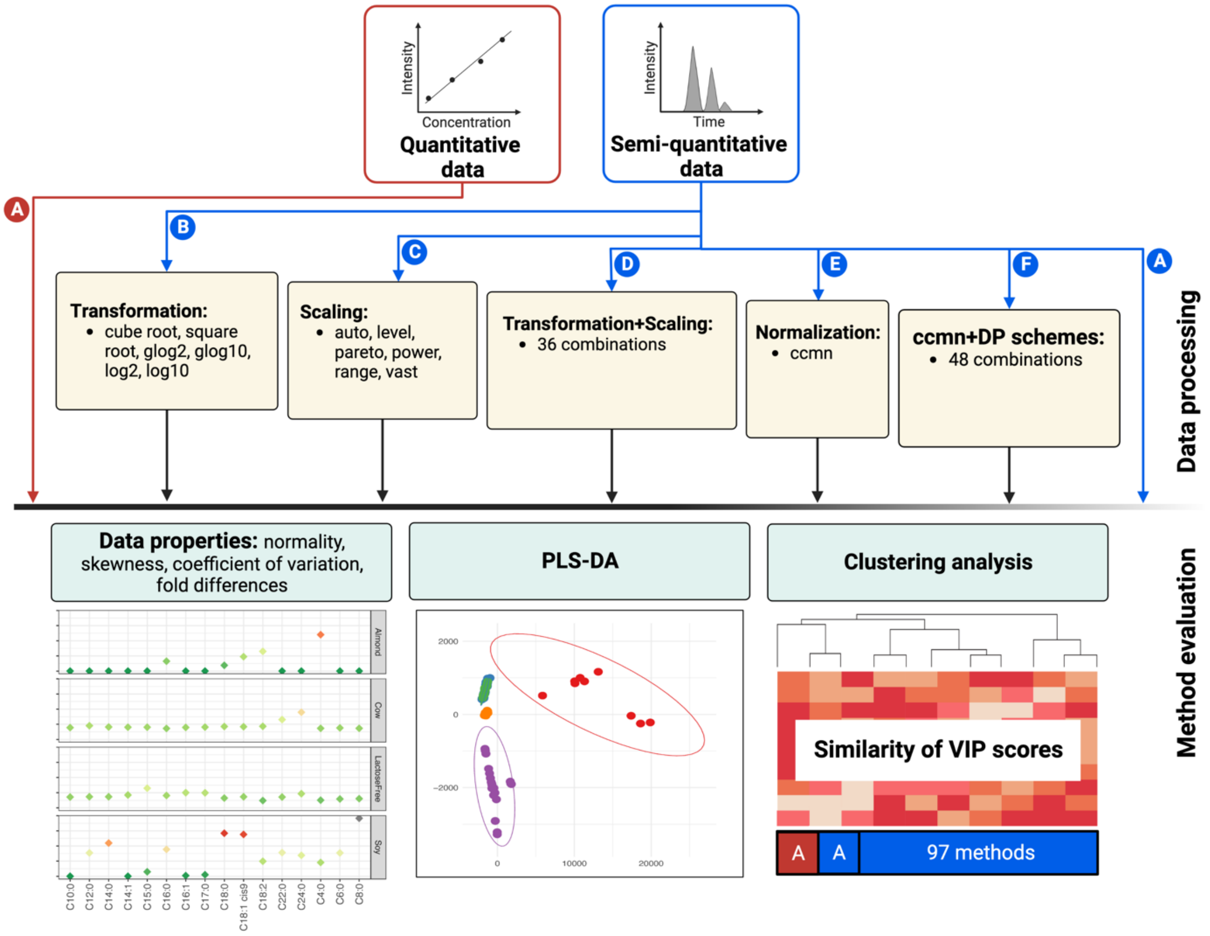

Figure 1. Analysis workflow. Different DP schemes were performed in this study, including (A) no processing, (B) transformation, (C) scaling, (D) transformation followed by scaling, (E) normalization by ccmn, and (F) consisting of ccmn+transform, ccmn+scale, and ccmn+transform+scale. The methods of each DP scheme were listed, and the number shown represents all combinations of these methods. The DP schemes were applied to semi-quantitative or peak area data (blue), and the method evaluations were then performed. This included effects on data properties and PLS-DA. For each data set, the resulting VIPs from PLS-DA were compared to those of the quantitative data (red).

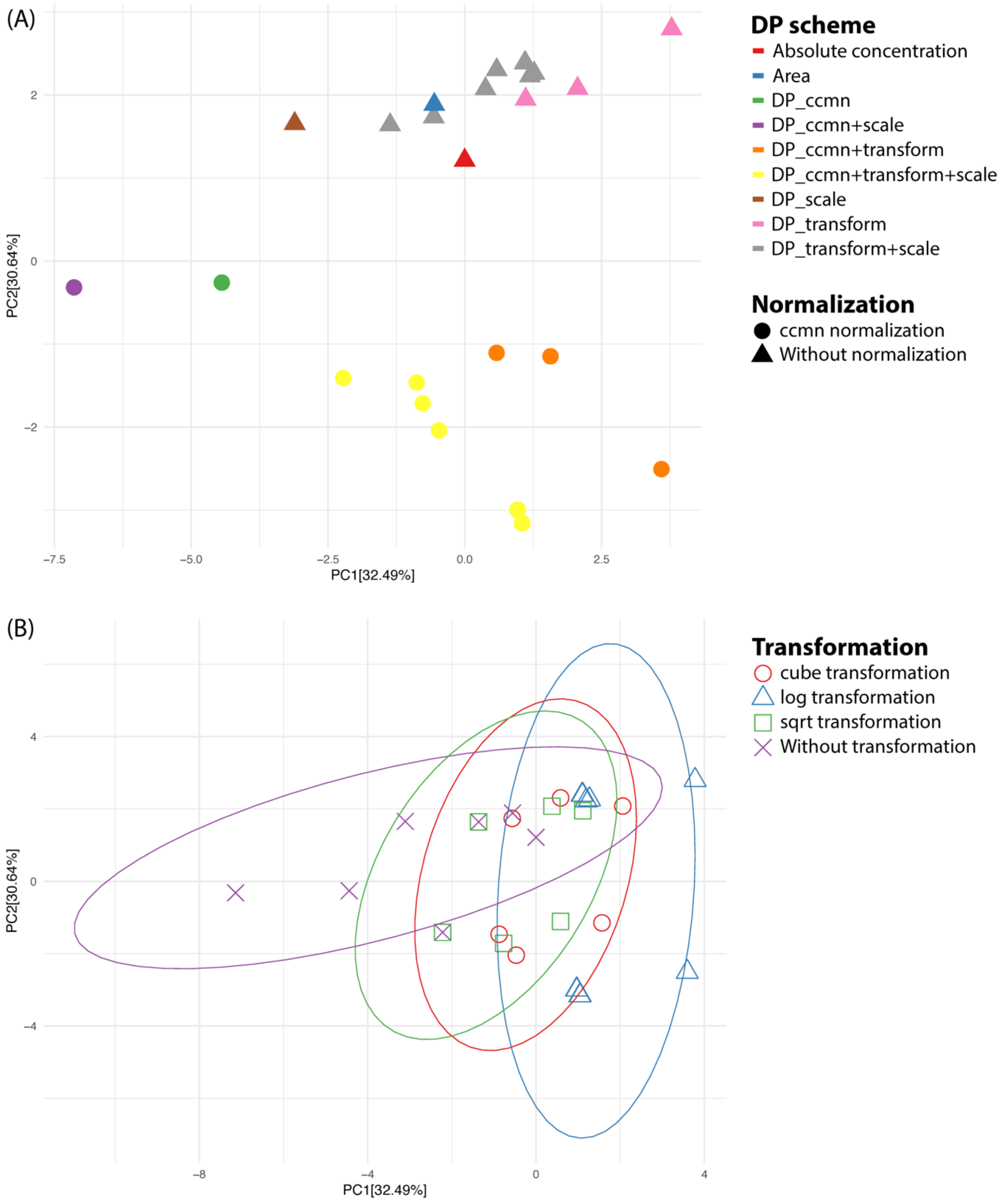

Figure 2. PCA score plots based on the data properties of absolute concentration, unprocessed (raw area), and processed milk data. (A) Major separation based on DP schemes; and (B) major separation based on transformation methods. The data properties included normality, skewness, and coefficient of variation.

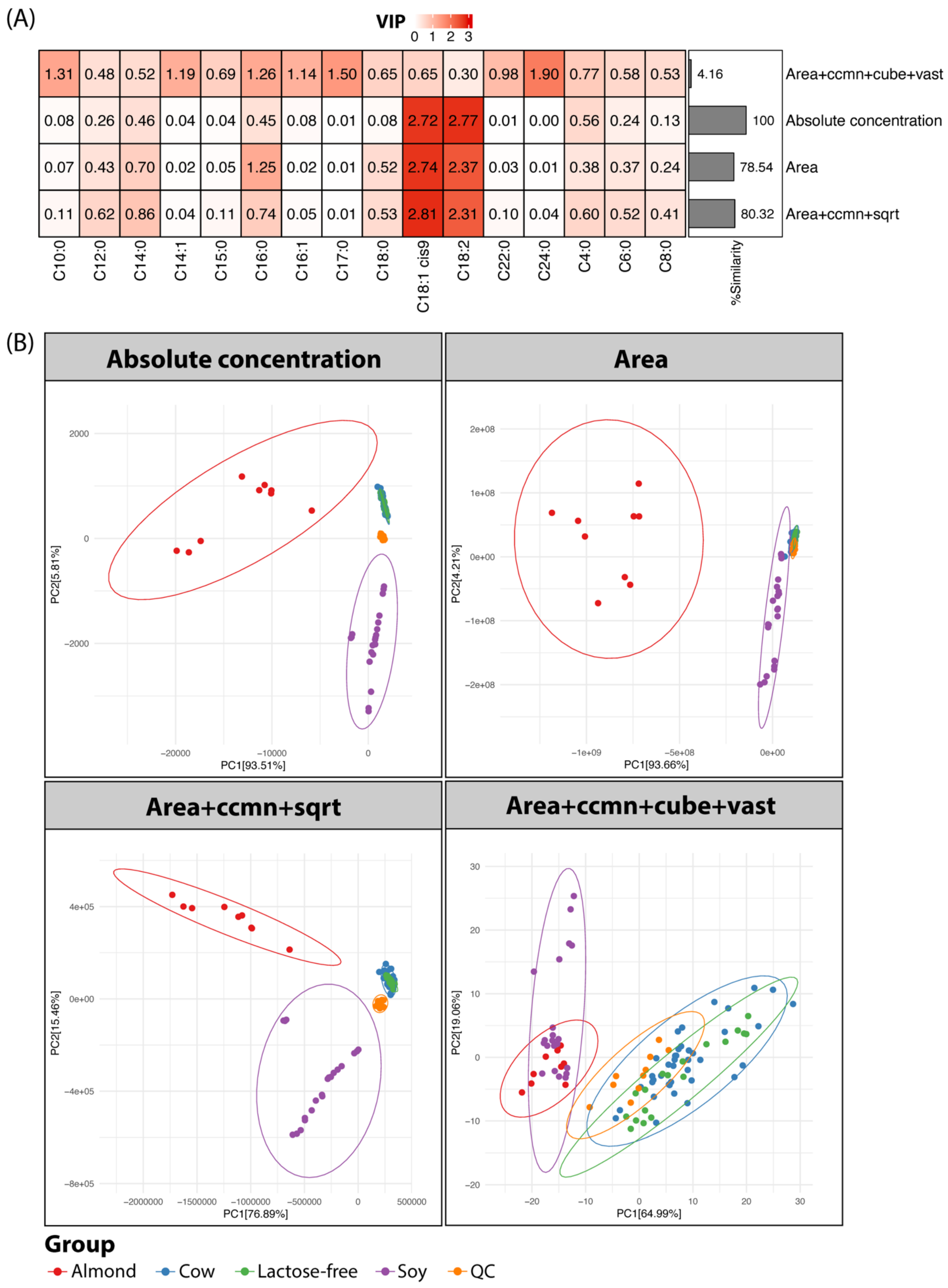

	C10:0	C12:0	C14:0	C14:1	C15:0	C16:0	C16:1	C17:0	C18:0	C18:1 cis9	C18:2	C22:0	C24:0	C4:0	C6:0	C8:0	%Similarity
Area+ccmn+cube+vast	1.31	0.48	0.52	1.19	0.69	1.26	1.14	1.50	0.65	0.65	0.30	0.98	1.90	0.77	0.58	0.53	4.16
Absolute concentration	0.08	0.26	0.46	0.04	0.04	0.45	0.08	0.01	0.08	2.72	2.77	0.01	0.00	0.56	0.24	0.13	100
Area	0.07	0.43	0.70	0.02	0.05	1.25	0.02	0.01	0.52	2.74	2.37	0.03	0.01	0.38	0.37	0.24	78.54
Area+ccmn+sqrt	0.11	0.62	0.86	0.04	0.11	0.74	0.05	0.01	0.53	2.81	2.31	0.10	0.04	0.60	0.52	0.41	80.32

Figure 3. Comparisons of selected DP schemes to absolute concentration and the raw milk area data represented by (A) clustering of VIPs and (B) PCA plots.

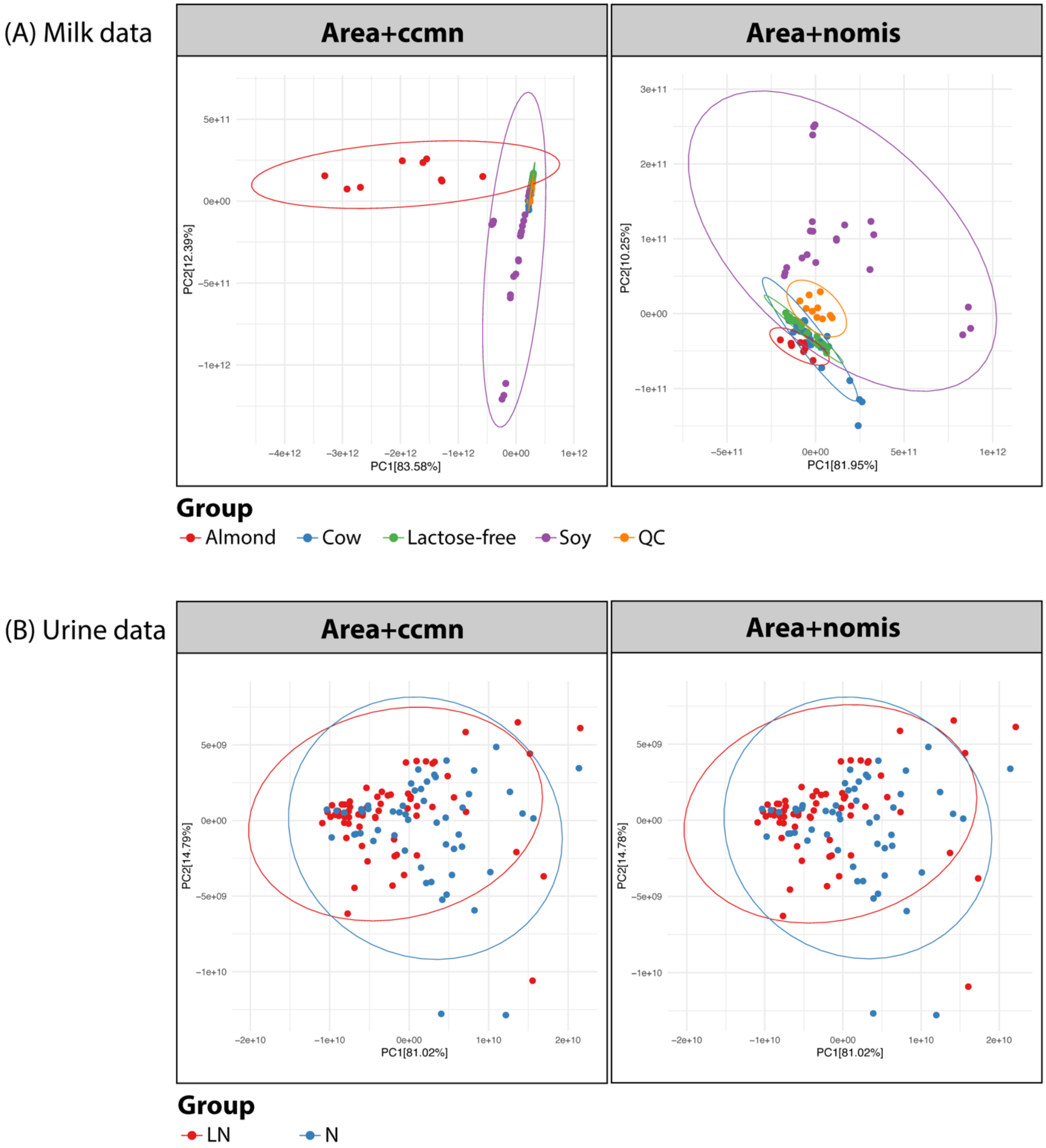

Figure 4. Effects of the ccmn and nomis normalization methods on the (A) milk data and (B) urine data. Color coding indicates sample groups, including the types of milk, the urine samples from healthy subjects (N), and patients with lupus nephritis (LN).

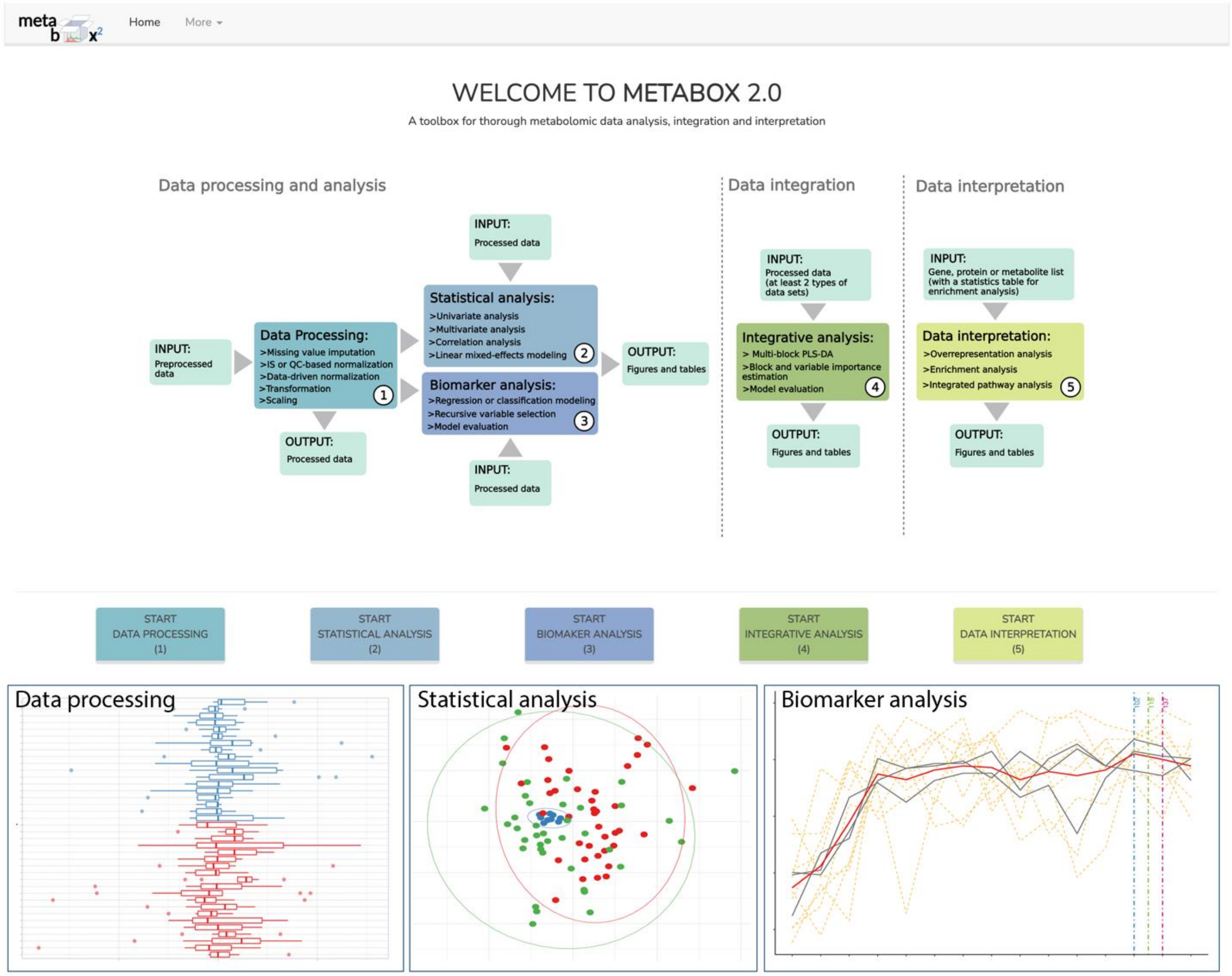

Figure 5. Metabox 2.0 GUI and example outputs from the data processing, statistical analysis, and biomarker analysis modules

Supplementary figures

Figure S1. Metabox 2.0 analysis pipelines. Metabox 2.0 provides three analysis pipelines: (A) data processing and analysis, (B) data integration, and (C) data interpretation. For each pipeline, the analysis module and its major tasks are presented in blue boxes. The data processing and analysis pipeline includes separated modules for data processing, statistical analysis and biomarker analysis. A comma-separated values (CSV) file is the required input format. The output contains a report file, related figures and tables.

Figure S2. The absolute concentration of 16 FAs in the milk samples.

Figure S3. Clustering of VIPs from absolute concentration, unprocessed and processed milk data by different DP schemes.

Figure S4. Comparisons of the selected DP schemes to the absolute concentration and unprocessed milk data represented by (A) across group RLA plots, (B) CV plots, and (C) fold differences of oleic acid to the other fatty acids. The CV of each metabolite is indicated by color coding from green to red. The large CV (CV > 1.0) is represented as a dark grey dot. The average CV (mCV) of each group is shown.

Figure S5. Clustering of VIPs from absolute concentration, unprocessed and processed milk data without the plant UFAs by different DP schemes.

Figure S6. Comparisons of the selected DP schemes to the absolute concentration and unprocessed milk data without the plant UFAs represented by (A) clustering of VIPs, (B) PCA plot, (C) across group RLA plots, (D) CV plots, and (E) fold differences of palmitic acid to the other fatty acids. The CV of each metabolite is indicated by color coding from green to red. The large CV (CV > 1.0) is represented as a dark grey dot. The average CV (mCV) of each group is shown.

Figure S7. PCA score plots based on the data properties of absolute concentration, raw area and processed urine data sets. (A) Major separation based on DP schemes, and (B) major separation based on transformation methods. The data properties included normality, skewness and coefficient of variation.

Figure S8. Clustering of VIPs from absolute concentration, unprocessed and processed urine data by different DP schemes.

Figure S9. Comparisons of the selected DP schemes to the absolute concentration and unprocessed urine data represented by (A) clustering of VIPs, (B) PCA plot, (C) across group RLA plots, (D) CV plots, and (E) fold differences of tryptophan to the other KP metabolites. The CV of each metabolite is indicated by color coding from green to red. The large CV (CV > 1.0) is represented as a dark grey dot. The average CV (mCV) of each group is shown. Healthy group (N) and lupus nephritis group (LN).

Click here to access/download
Supplementary Material
FigureS1.pdf

Supplementary Material_FigureS2

Click here to access/download
Supplementary Material
FigureS2.pdf

Supplementary Material_FigureS3

Click here to access/download
Supplementary Material
FigureS3.pdf

Click here to access/download
Supplementary Material
FigureS4.pdf

Supplementary Material_FigureS5

Click here to access/download
Supplementary Material
FigureS5.pdf

Click here to access/download
Supplementary Material
FigureS6.pdf

Click here to access/download
Supplementary Material
FigureS7.pdf

Supplementary Material_FigureS8

Click here to access/download
Supplementary Material
FigureS8.pdf

Supplementary Material_FigureS9

Click here to access/download
Supplementary Material
FigureS9.pdf

Click here to access/download
Supplementary Material
TableS1-2.docx

Click here to access/download
Supplementary Material
TableS3-7.xlsx

Cover letter Click here to access/download;Personal Cover;Coverletter-29-08-2023.docx

Mahidol University
Faculty of Medicine
Siriraj Hospital

19-08-2023

Dear Editor,

On behalf of the authors, I herewith submit our manuscript entitled "Metabox 2.0: The data processing solution that renders metabolomics more quantitative" to be considered for publication in GigaScience journal.

This study is the first to use the quantitative data as a reference point for data processing evaluation. In addition to the effects of data processing schemes on data properties and variance structure, we evaluated several well-known data processing methods to produce outputs that were most similar to the corresponding absolute quantified data. The most desirable data processing scheme is the one that results in highly identical statistical results between the processed data and its quantitative companion. We used two metabolomic data sets representing different types of data matrices: data relating to a food product with definitive markers and clinical metabolomic data with indistinct variations. The internal standard-based normalization, such as the cross-contribution compensating multiple standard normalization (ccmn) method, was the best option to improve this clinical metabolomics data. The ccmn followed by square root transformation outperformed for the data sets with apparent markers and a known biological effect. Lastly, Metabox 2.0 was introduced, which contains significant updates to the first version on the data processing tasks, biomarker identification, and integrative analysis. It was an underlying tool for all analyses in this study.

Considering the above, we are confident that our manuscript is of interest to researchers in metabolomics and other omics field. The results obtained could be a useful and unbiased reference for data processing recommendations. This manuscript contains unpublished material. All the figures contained in the article are genuine and have not been published anywhere in any form. All authors have approved the submission of the manuscript.

Sincerely yours,

Sakda Khoomrung, PhD
Corresponding Author
Siriraj Center of Research Excellent in Metabolomics and Systems Biology (SiCORE-MSB), Faculty of Medicine Siriraj Hospital, Mahidol University, Bangkok 10700, Thailand
http://metsysbio.com
