# Supplementary material for: Data processing solutions to render metabolomics more quantitative: case studies in food and clinical metabolomics using Metabox 2.0
Source: Gigascience. 2024 Mar 15;13:giae005. doi: 10.1093/gigascience/giae005 (PMC10941642; doi:10.1093/gigascience/giae005)
Supplement: giae005_Supplemental_Files [file giae005_supplemental_files.zip › R1_FigureS4.pdf]

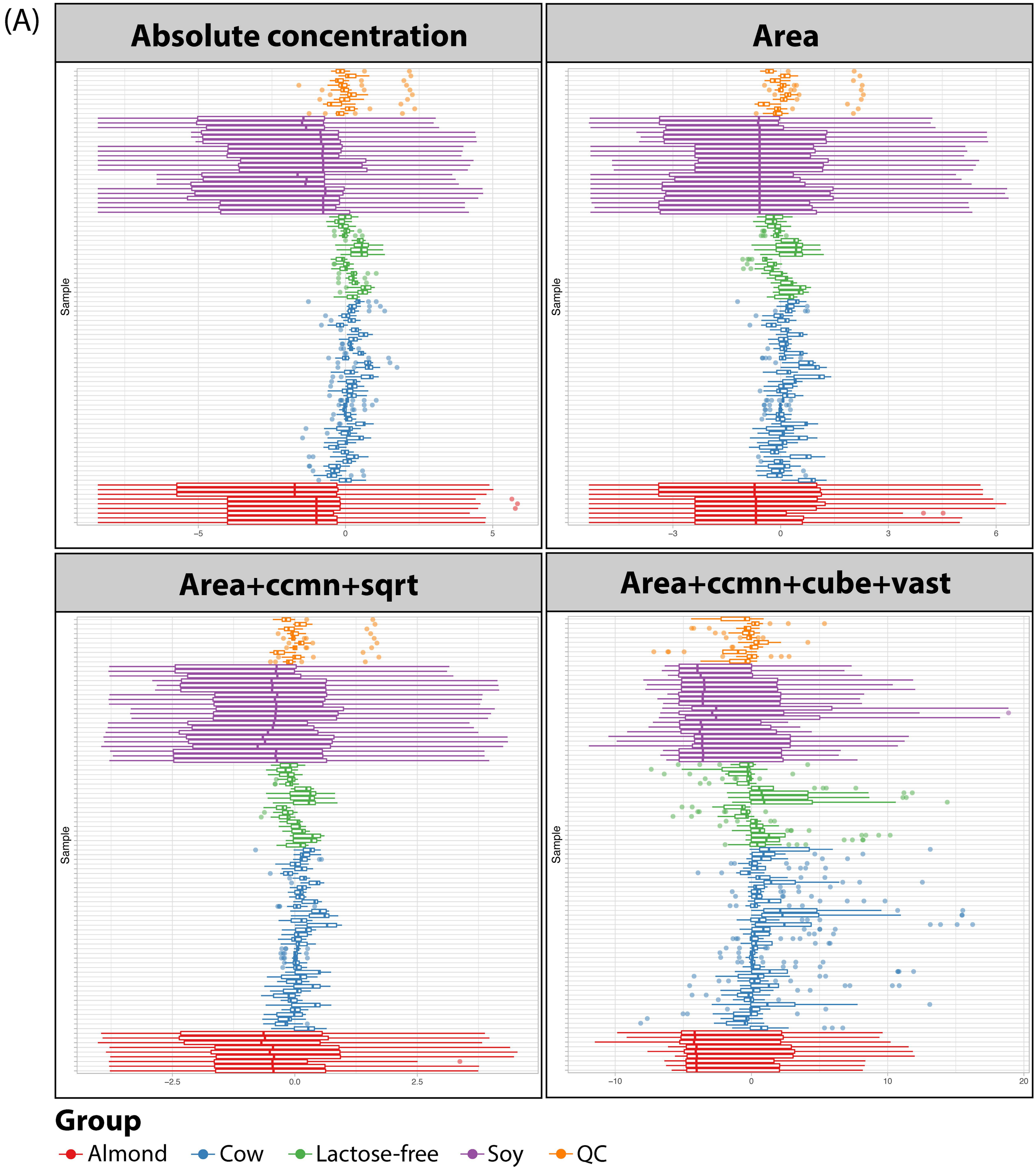
(A)
Absolute concentration
Area
Area+ccmn+sqrt
Area+ccmn+cube+vast
Sample
−5
0
5
−3
0
3
6
−2.5
0.0
2.5
−10
0
10
20
Group
Almond
Cow
Lactose-free
Soy
QC

(B)

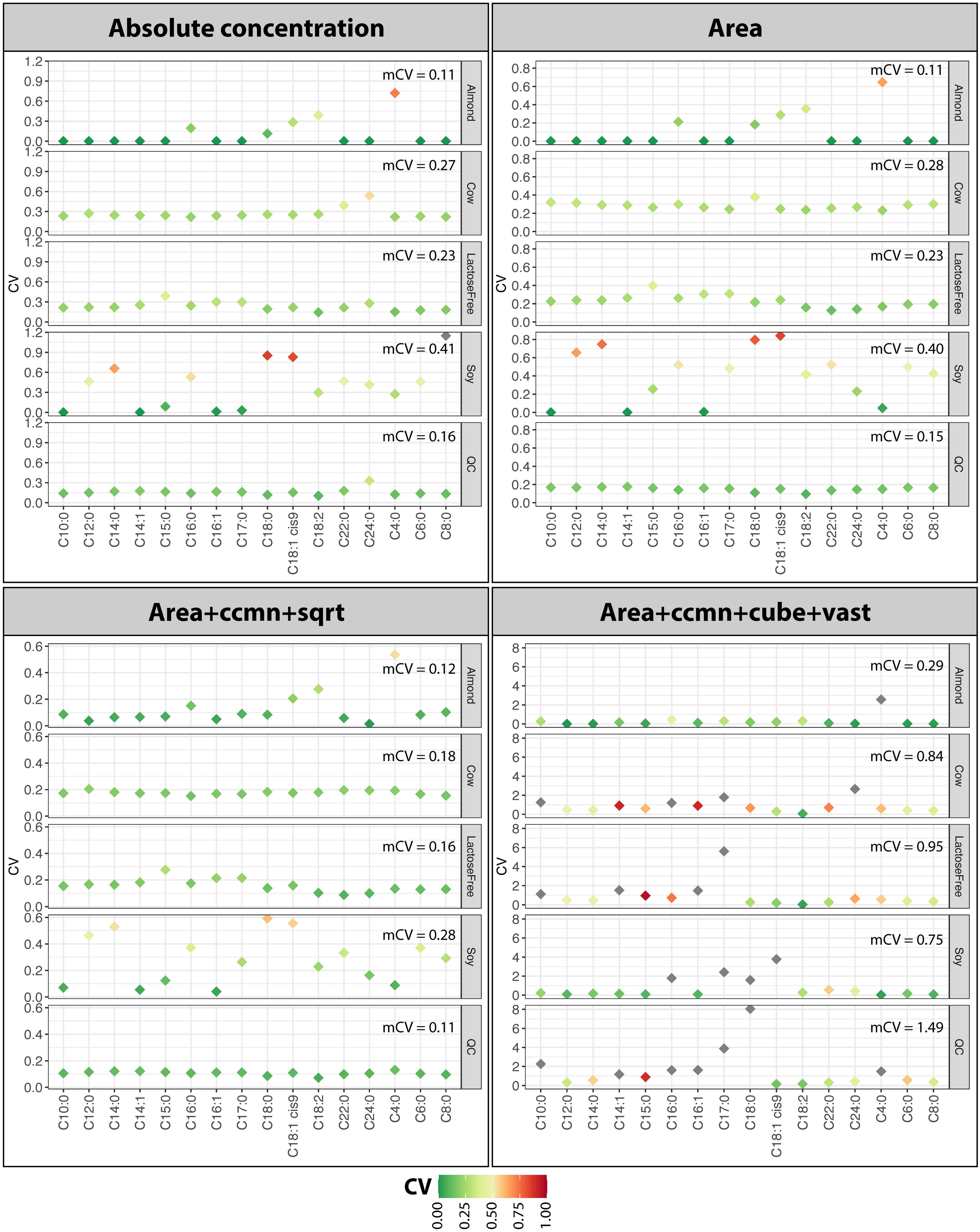
Absolute concentration
Area
Area+ccmn+sqrt
Area+ccmn+cube+vast
Almond
Cow
LactoseFree
Soy
QC
CV
mCV = 0.11
mCV = 0.27
mCV = 0.23
mCV = 0.41
mCV = 0.16
mCV = 0.11
mCV = 0.28
mCV = 0.23
mCV = 0.40
mCV = 0.15
mCV = 0.12
mCV = 0.18
mCV = 0.16
mCV = 0.28
mCV = 0.11
mCV = 0.29
mCV = 0.84
mCV = 0.95
mCV = 0.75
mCV = 1.49
C10:0
C12:0
C14:0
C14:1
C15:0
C16:0
C16:1
C17:0
C18:0
C18:1 cis9
C18:2
C22:0
C24:0
C4:0
C6:0
C8:0
0.00
0.25
0.50
0.75
1.00

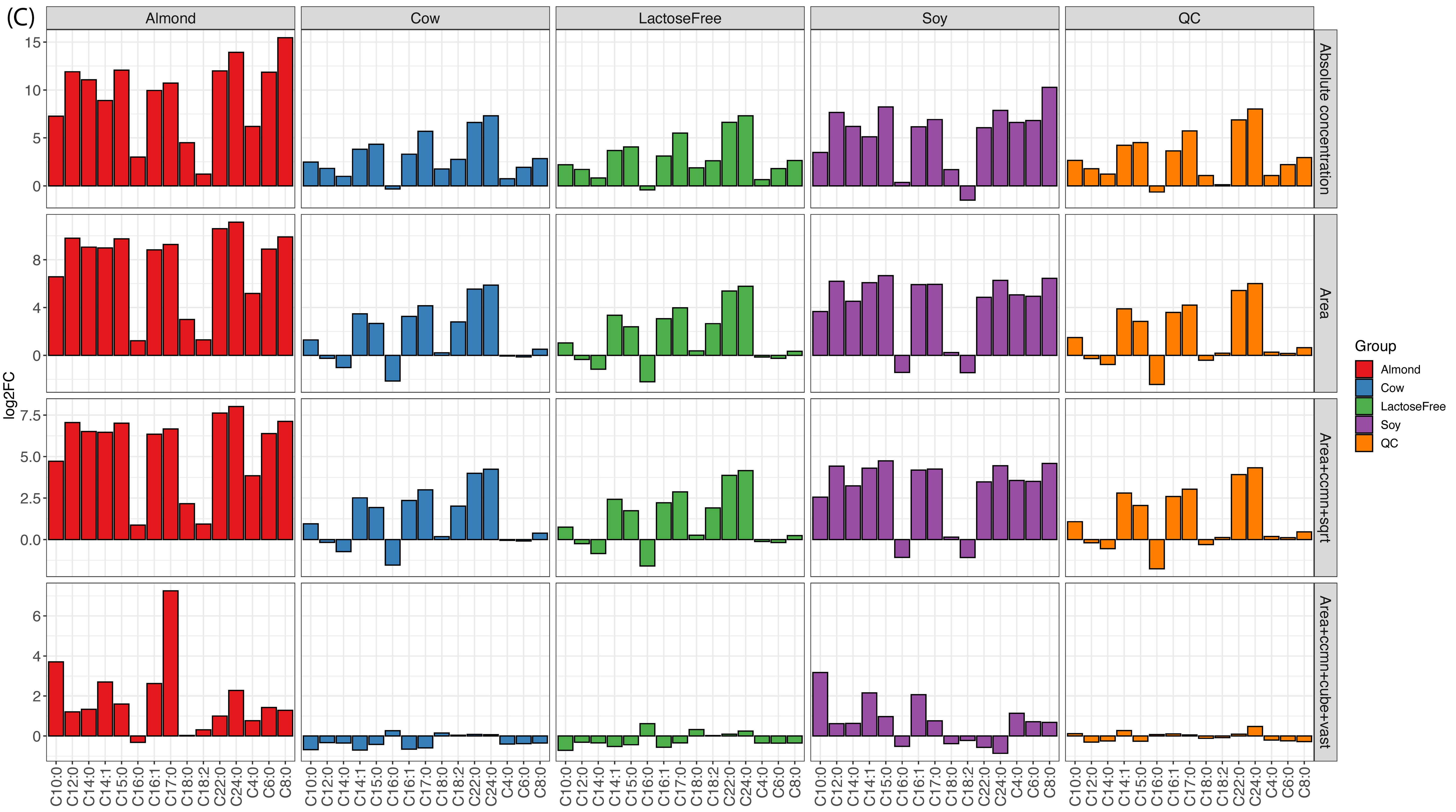
(C)
Almond
Cow
LactoseFree
Soy
QC
Absolute concentration
Area
Area+ccmn+sqrt
Area+ccmn+cube+vast
log2FC
Group
Almond
Cow
LactoseFree
Soy
QC
C10:0 C12:0 C14:0 C14:1 C15:0 C16:0 C16:1 C17:0 C18:0 C18:2 C22:0 C24:0 C4:0 C6:0 C8:0
