# Supplementary material for: Data processing solutions to render metabolomics more quantitative: case studies in food and clinical metabolomics using Metabox 2.0
Source: Gigascience. 2024 Mar 15;13:giae005. doi: 10.1093/gigascience/giae005 (PMC10941642; doi:10.1093/gigascience/giae005)
Supplement: giae005_Supplemental_Files [file giae005_supplemental_files.zip › R1_FigureS6.pdf]

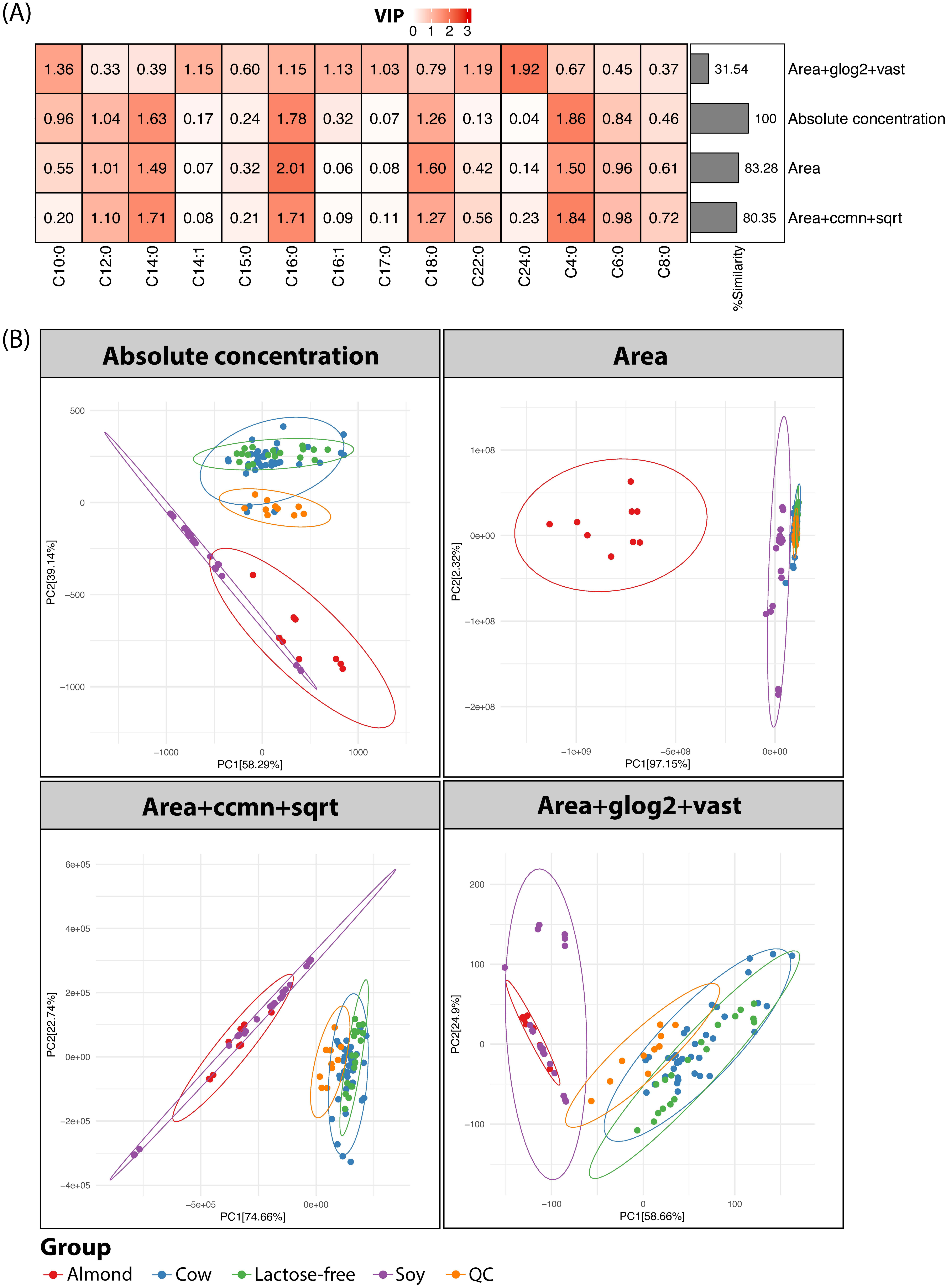
(A)
VIP
0 1 2 3
1.36 0.33 0.39 1.15 0.60 1.15 1.13 1.03 0.79 1.19 1.92 0.67 0.45 0.37
0.96 1.04 1.63 0.17 0.24 1.78 0.32 0.07 1.26 0.13 0.04 1.86 0.84 0.46
0.55 1.01 1.49 0.07 0.32 2.01 0.06 0.08 1.60 0.42 0.14 1.50 0.96 0.61
0.20 1.10 1.71 0.08 0.21 1.71 0.09 0.11 1.27 0.56 0.23 1.84 0.98 0.72
31.54 Area+glog2+vast
100 Absolute concentration
83.28 Area
80.35 Area+ccmn+sqrt
C10:0 C12:0 C14:0 C14:1 C15:0 C16:0 C16:1 C17:0 C18:0 C22:0 C24:0 C4:0 C6:0 C8:0
%Similarity
(B)
Absolute concentration
PC1[58.29%]
PC2[39.14%]
Area
PC1[97.15%]
PC2[2.32%]
Area+ccmn+sqrt
PC1[74.66%]
PC2[22.74%]
Area+glog2+vast
PC1[58.66%]
PC2[24.9%]
Group
Almond
Cow
Lactose-free
Soy
QC

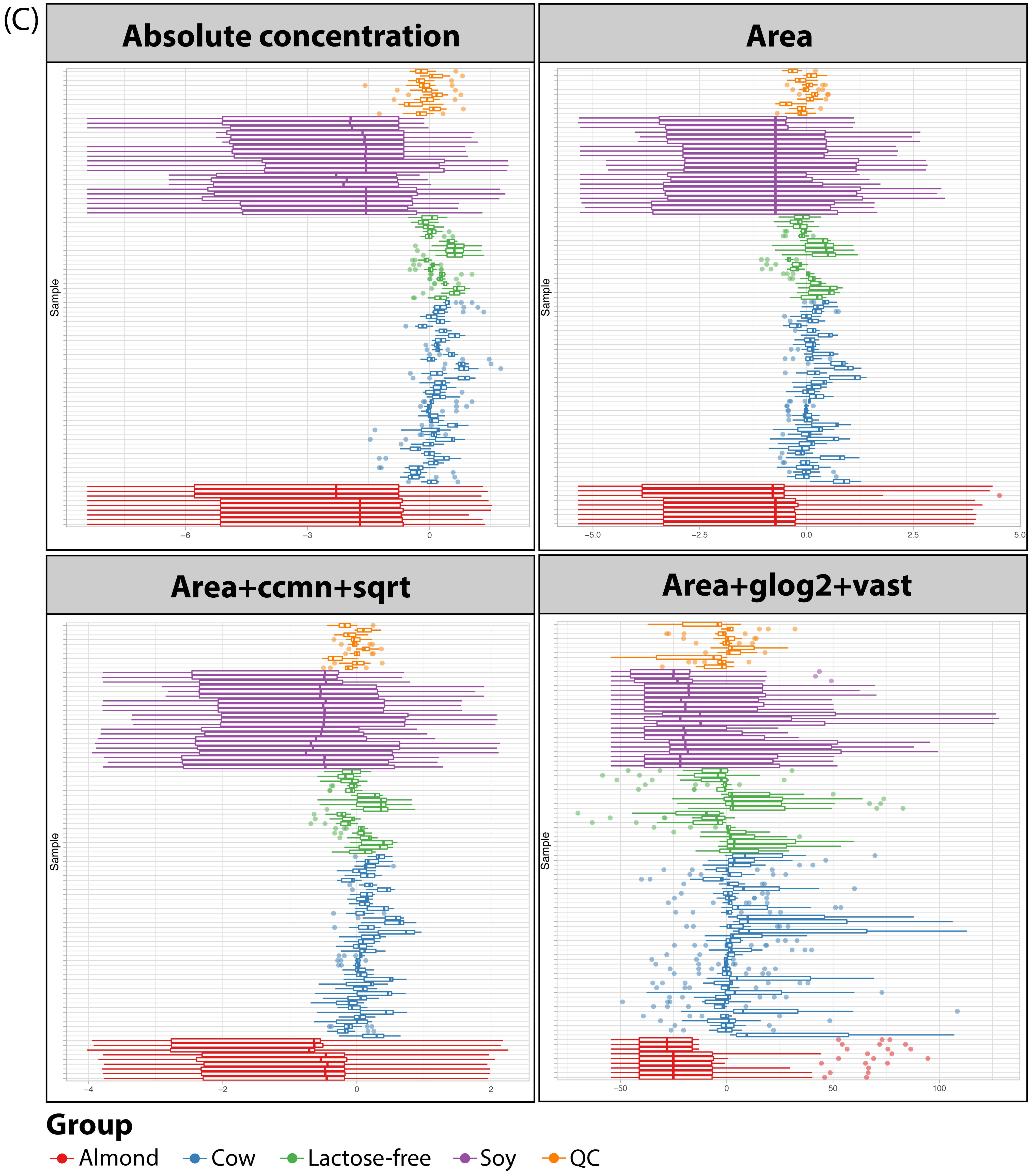
(C)
Absolute concentration
Area
Area+ccmn+sqrt
Area+glog2+vast
Sample
−6
−3
0
−5.0
−2.5
0.0
2.5
5.0
−4
−2
0
2
−50
0
50
100
Group
Almond
Cow
Lactose-free
Soy
QC

(D)

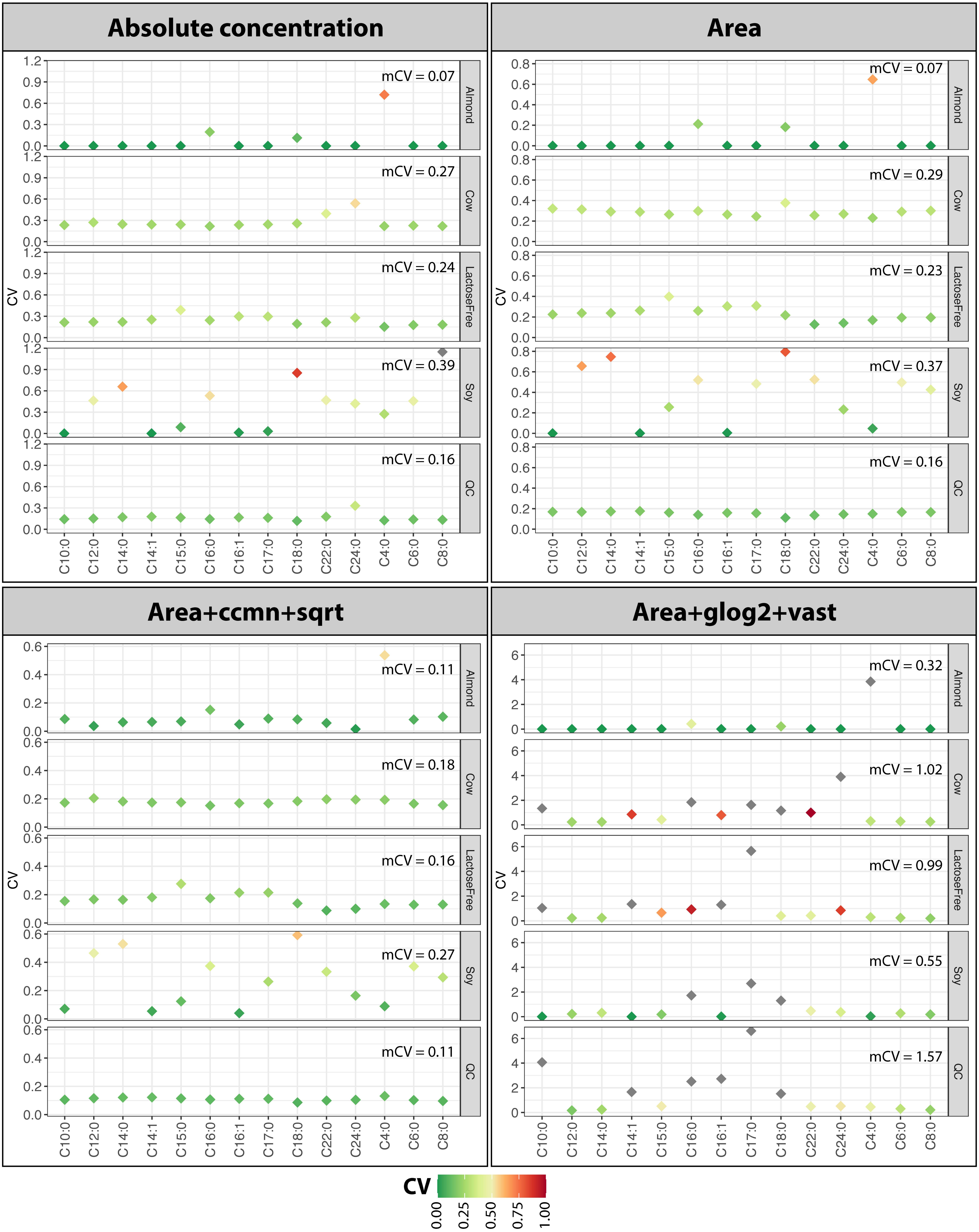
Absolute concentration
Area
Area+ccmn+sqrt
Area+glog2+vast
Almond
Cow
LactoseFree
Soy
QC
CV
mCV = 0.07
mCV = 0.27
mCV = 0.24
mCV = 0.39
mCV = 0.16
mCV = 0.07
mCV = 0.29
mCV = 0.23
mCV = 0.37
mCV = 0.16
mCV = 0.11
mCV = 0.18
mCV = 0.16
mCV = 0.27
mCV = 0.11
mCV = 0.32
mCV = 1.02
mCV = 0.99
mCV = 0.55
mCV = 1.57
C10:0
C12:0
C14:0
C14:1
C15:0
C16:0
C16:1
C17:0
C18:0
C22:0
C24:0
C4:0
C6:0
C8:0
0.00
0.25
0.50
0.75
1.00

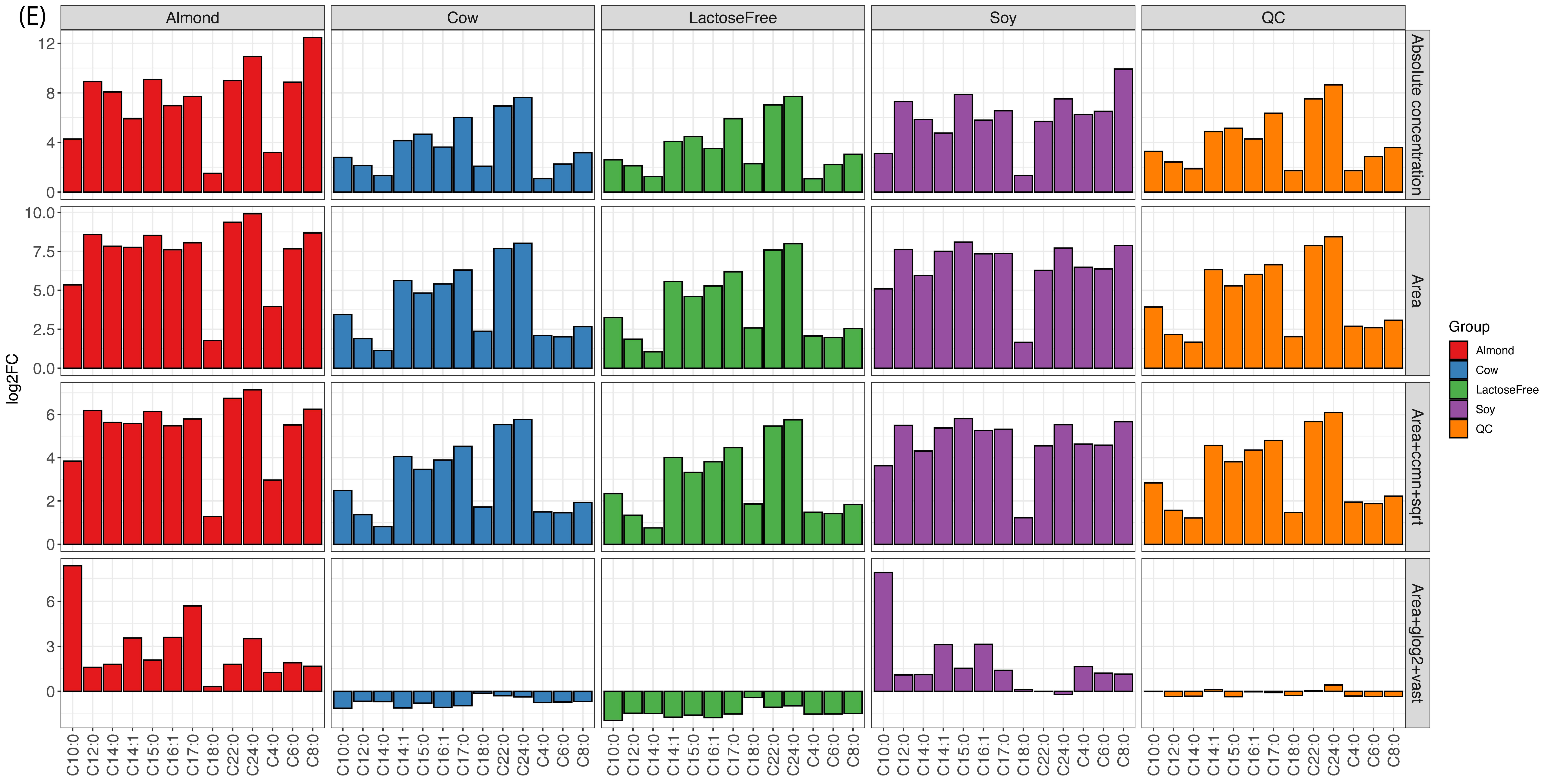

(E)
Almond
Cow
LactoseFree
Soy
QC
Absolute concentration
Area
Area+ccmn+sqrt
Area+glog2+vast
log2FC
Group
Almond
Cow
LactoseFree
Soy
QC
C10:0
C12:0
C14:0
C14:1
C15:0
C16:1
C17:0
C18:0
C22:0
C24:0
C4:0
C6:0
C8:0
