# Supplementary material for: Data processing solutions to render metabolomics more quantitative: case studies in food and clinical metabolomics using Metabox 2.0
Source: Gigascience. 2024 Mar 15;13:giae005. doi: 10.1093/gigascience/giae005 (PMC10941642; doi:10.1093/gigascience/giae005)
Supplement: giae005_Supplemental_Files [file giae005_supplemental_files.zip › R1_FigureS8.pdf]

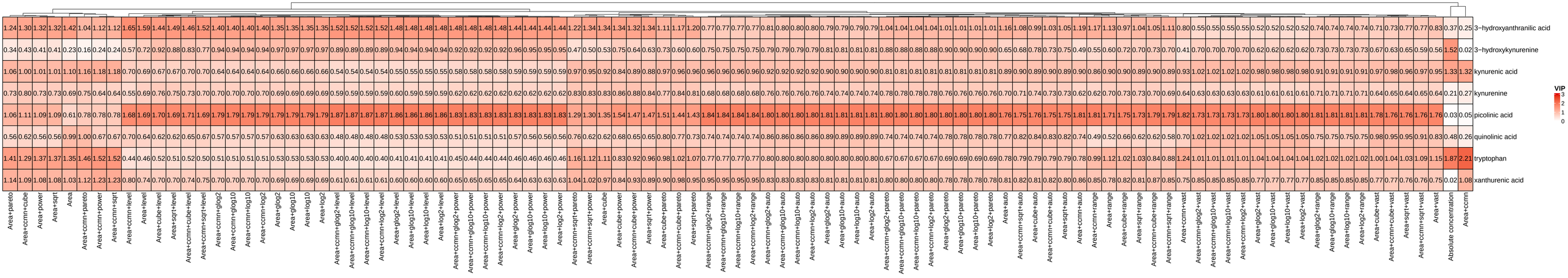
1.24 1.30 1.32 1.32 1.42 1.04 1.12 1.12 1.65 1.59 1.44 1.49 1.46 1.52 1.40 1.40 1.40 1.40 1.35 1.35 1.35 1.35 1.52 1.52 1.52 1.52 1.48 1.48 1.48 1.48 1.48 1.48 1.48 1.48 1.48 1.44 1.44 1.44 1.44 1.22 1.34 1.34 1.34 1.32 1.34 1.11 1.17 1.20 0.77 0.77 0.77 0.77 0.81 0.80 0.80 0.80 0.79 0.79 0.79 0.79 1.04 1.04 1.04 1.04 1.01 1.01 1.01 1.01 1.16 1.08 0.99 1.03 1.05 1.19 1.17 1.13 0.97 1.04 1.05 1.11 0.80 0.55 0.55 0.55 0.55 0.52 0.52 0.52 0.52 0.74 0.74 0.74 0.74 0.71 0.73 0.77 0.77 0.83 0.37 0.25 3−hydroxyanthranilic acid
0.34 0.43 0.41 0.41 0.23 0.16 0.24 0.24 0.57 0.72 0.92 0.88 0.83 0.77 0.94 0.94 0.94 0.94 0.97 0.97 0.97 0.97 0.89 0.89 0.89 0.89 0.94 0.94 0.94 0.94 0.92 0.92 0.92 0.92 0.96 0.95 0.95 0.95 0.47 0.50 0.53 0.75 0.64 0.63 0.73 0.60 0.60 0.75 0.75 0.75 0.79 0.79 0.79 0.79 0.81 0.81 0.81 0.81 0.88 0.88 0.88 0.88 0.90 0.90 0.90 0.90 0.65 0.68 0.78 0.73 0.75 0.49 0.55 0.60 0.72 0.70 0.73 0.70 0.41 0.70 0.70 0.70 0.70 0.62 0.62 0.62 0.62 0.73 0.73 0.73 0.73 0.67 0.63 0.65 0.59 0.56 1.52 0.02 3−hydroxykynurenine
1.06 1.00 1.01 1.01 1.10 1.16 1.18 1.18 0.70 0.69 0.67 0.67 0.70 0.70 0.64 0.64 0.64 0.64 0.66 0.66 0.66 0.66 0.54 0.54 0.54 0.54 0.55 0.55 0.55 0.55 0.58 0.58 0.58 0.58 0.59 0.59 0.59 0.59 0.97 0.95 0.92 0.84 0.89 0.88 0.97 0.96 0.96 0.96 0.96 0.96 0.96 0.96 0.92 0.92 0.92 0.92 0.90 0.90 0.90 0.90 0.81 0.81 0.81 0.81 0.81 0.81 0.81 0.81 0.81 0.89 0.90 0.89 0.90 0.89 0.90 0.86 0.90 0.90 0.89 0.90 0.89 0.93 1.02 1.02 1.02 1.02 0.98 0.98 0.98 0.91 0.91 0.91 0.91 0.97 0.98 0.96 0.97 0.95 1.33 1.32 kynurenic acid
0.73 0.80 0.73 0.73 0.69 0.75 0.64 0.64 0.55 0.69 0.76 0.75 0.73 0.70 0.70 0.70 0.70 0.70 0.69 0.69 0.69 0.69 0.59 0.59 0.59 0.59 0.60 0.59 0.59 0.59 0.62 0.62 0.62 0.62 0.62 0.62 0.62 0.62 0.83 0.83 0.83 0.86 0.88 0.84 0.77 0.84 0.81 0.68 0.68 0.68 0.68 0.76 0.76 0.76 0.76 0.74 0.74 0.74 0.74 0.78 0.78 0.78 0.78 0.76 0.76 0.76 0.76 0.70 0.71 0.74 0.73 0.73 0.62 0.62 0.70 0.73 0.73 0.70 0.69 0.64 0.63 0.63 0.63 0.63 0.61 0.61 0.61 0.61 0.71 0.71 0.71 0.71 0.64 0.65 0.64 0.65 0.64 0.21 0.27 kynurenine
1.06 1.11 1.09 1.09 0.61 0.78 0.78 0.78 1.68 1.69 1.70 1.69 1.71 1.69 1.79 1.79 1.79 1.79 1.79 1.79 1.79 1.79 1.87 1.87 1.87 1.87 1.86 1.86 1.86 1.86 1.83 1.83 1.83 1.83 1.83 1.83 1.83 1.83 1.29 1.30 1.35 1.54 1.47 1.47 1.51 1.44 1.43 1.84 1.84 1.84 1.84 1.80 1.80 1.80 1.80 1.81 1.81 1.81 1.81 1.80 1.80 1.80 1.80 1.80 1.80 1.80 1.80 1.80 1.80 1.80 1.81 1.81 1.71 1.75 1.73 1.79 1.79 1.82 1.73 1.73 1.73 1.73 1.80 1.80 1.80 1.80 1.81 1.81 1.81 1.81 1.78 1.76 1.76 1.76 1.76 0.03 0.05 picolinic acid
0.56 0.62 0.56 0.56 0.99 1.00 0.67 0.67 0.70 0.64 0.62 0.62 0.65 0.67 0.57 0.57 0.57 0.57 0.63 0.63 0.63 0.63 0.48 0.48 0.48 0.48 0.53 0.53 0.53 0.53 0.51 0.51 0.51 0.51 0.57 0.56 0.56 0.56 0.76 0.62 0.62 0.68 0.65 0.65 0.80 0.77 0.73 0.74 0.74 0.74 0.74 0.86 0.86 0.86 0.86 0.89 0.89 0.89 0.89 0.74 0.74 0.74 0.74 0.78 0.78 0.78 0.78 0.77 0.82 0.84 0.83 0.82 0.74 0.49 0.52 0.66 0.62 0.62 0.58 0.70 1.02 1.02 1.02 1.02 1.05 1.05 1.05 1.05 0.75 0.75 0.75 0.75 0.98 0.95 0.95 0.91 0.83 0.48 0.26 quinolinic acid
1.41 1.29 1.37 1.37 1.35 1.46 1.52 1.52 0.44 0.46 0.52 0.51 0.52 0.50 0.51 0.51 0.51 0.51 0.53 0.53 0.53 0.53 0.40 0.40 0.40 0.40 0.41 0.41 0.41 0.41 0.45 0.44 0.44 0.44 0.46 0.46 0.46 0.46 1.16 1.12 1.11 0.83 0.92 0.96 0.98 1.02 1.07 0.77 0.77 0.77 0.77 0.80 0.80 0.80 0.80 0.80 0.80 0.80 0.80 0.67 0.67 0.67 0.67 0.69 0.69 0.69 0.69 0.78 0.79 0.79 0.79 0.78 0.99 1.12 1.02 1.03 0.84 0.88 1.24 1.01 1.01 1.01 1.01 1.04 1.04 1.04 1.04 1.02 1.02 1.02 1.02 1.00 1.04 1.03 1.09 1.15 1.87 2.21 tryptophan
1.14 1.09 1.08 1.08 1.03 1.12 1.23 1.23 0.80 0.74 0.70 0.70 0.74 0.75 0.70 0.70 0.70 0.70 0.69 0.69 0.69 0.69 0.61 0.61 0.61 0.61 0.60 0.60 0.60 0.60 0.65 0.65 0.65 0.65 0.64 0.63 0.63 0.63 1.04 1.02 0.97 0.84 0.93 0.89 0.90 0.98 0.95 0.95 0.95 0.95 0.83 0.83 0.83 0.83 0.81 0.81 0.81 0.81 0.80 0.80 0.80 0.80 0.80 0.80 0.80 0.80 0.78 0.78 0.78 0.78 0.81 0.82 0.81 0.82 0.80 0.86 0.85 0.78 0.82 0.81 0.87 0.85 0.75 0.85 0.85 0.85 0.85 0.77 0.77 0.76 0.76 0.75 0.02 1.08 xanthurenic acid
Area+pareto
Area+ccmn+cube
Area+power
Area+sqrt
Area
Area+ccmn+pareto
Area+ccmn+power
Area+ccmn+sqrt
Area+ccmn+level
Area+level
Area+cube+level
Area+sqrt+level
Area+ccmn+cube+level
Area+ccmn+sqrt+level
Area+ccmn+glog2
Area+ccmn+glog10
Area+ccmn+log10
Area+ccmn+log2
Area+glog2
Area+glog10
Area+log10
Area+log2
Area+ccmn+glog2+level
Area+ccmn+glog10+level
Area+ccmn+log10+level
Area+ccmn+log2+level
Area+glog2+level
Area+glog10+level
Area+log10+level
Area+log2+level
Area+ccmn+glog2+power
Area+ccmn+glog10+power
Area+ccmn+log10+power
Area+ccmn+log2+power
Area+glog2+power
Area+glog10+power
Area+log10+power
Area+log2+power
Area+ccmn+sqrt+pareto
Area+ccmn+sqrt+power
Area+cube
Area+cube+power
Area+ccmn+cube+power
Area+sqrt+power
Area+cube+pareto
Area+ccmn+cube+pareto
Area+sqrt+pareto
Area+ccmn+glog2+range
Area+ccmn+glog10+range
Area+ccmn+log2+range
Area+ccmn+glog2+auto
Area+ccmn+glog10+auto
Area+ccmn+log10+auto
Area+ccmn+log2+auto
Area+glog2+auto
Area+glog10+auto
Area+log10+auto
Area+log2+auto
Area+ccmn+glog2+pareto
Area+ccmn+glog10+pareto
Area+ccmn+log10+pareto
Area+ccmn+log2+pareto
Area+glog2+pareto
Area+glog10+pareto
Area+log10+pareto
Area+log2+pareto
Area+auto
Area+ccmn+sqrt+auto
Area+cube+auto
Area+ccmn+cube+auto
Area+sqrt+auto
Area+ccmn+auto
Area+ccmn+range
Area+range
Area+cube+range
Area+sqrt+range
Area+ccmn+cube+range
Area+ccmn+sqrt+range
Area+ccmn+vast
Area+ccmn+glog2+vast
Area+ccmn+glog10+vast
Area+ccmn+log10+vast
Area+ccmn+log2+vast
Area+glog2+vast
Area+glog10+vast
Area+log10+vast
Area+log2+vast
Area+glog2+range
Area+glog10+range
Area+log10+range
Area+log2+range
Area+cube+vast
Area+ccmn+cube+vast
Area+sqrt+vast
Area+ccmn+sqrt+vast
Area+vast
Absolute concentration
Area+ccmn
VIP
3
2
1
0
