# Supplementary material for: Data processing solutions to render metabolomics more quantitative: case studies in food and clinical metabolomics using Metabox 2.0
Source: Gigascience. 2024 Mar 15;13:giae005. doi: 10.1093/gigascience/giae005 (PMC10941642; doi:10.1093/gigascience/giae005)
Supplement: giae005_Supplemental_Files [file giae005_supplemental_files.zip › R1_TableS1-2.docx]

**Table S1.** List of DP methods in Metabox 2.0

| **Method** | **R-function** | **Description** | **Detail** | **Module** | **Reference** |
| --- | --- | --- | --- | --- | --- |
| zero, halfmin, min, mean, median | impute_missing_data | Impute missing values | Single value replacement | Missing value imputation | (1) |
| random forest (RF), k-nearest neighbors (KNN) | impute_missing_data | Impute missing values | Local similarity approach | Missing value imputation | (2,3) |
| singular value decomposition (SVD), probabilistic principal component analysis (PPCA), bayesian principal component analysis (BPCA) | impute_missing_data | Impute missing values | Global-structure approach | Missing value imputation | (4) |
| cross-contribution compensating multiple standard normalization (CCMN) | normalize_input_data_byqc | Perform IS-based normalization | Removing unwanted systematic variation and compensating cross-contribution based on internal standard(s) and the experimental design | IS- or QC-based normalization | (5) |
| normalization using optimal selection of multiple internal standards (NOMIS) | normalize_input_data_byqc | Perform IS-based normalization | Removing unwanted systematic variation based on variabilities of internal standard(s) | IS- or QC-based normalization | (6) |
| removal of unwanted variation-random (RUV-random) | normalize_input_data_byqc | Perform IS-based normalization | Removing overall unwanted variation based on quality control metabolites | IS- or QC-based normalization | (7) |
| systematic error removal using random forest (SERRF) | normalize_input_data_byqc | Perform QC-based normalization | Removing unwanted systematic variation based on QC samples using the RF algorithm | IS- or QC-based normalization | (8) |
| local polynomial regression (LOESS) | normalize_input_data_byqc | Perform QC-based normalization | Removing unwanted systematic variation based on QC samples using the locally weighted regression | IS- or QC-based normalization | (9) |
| contrast normalization | normalize_input_data_bydata | Perform data-driven normalization | Nonlinear normalization for normalizing feature intensities using smooth curves | Data-driven normalization | (10) |
| cubic splines normalization | normalize_input_data_bydata | Perform data-driven normalization | Nonlinear baseline normalization producing a similar distribution of feature intensities | Data-driven normalization | (11) |
| EigenMS | normalize_input_data_bydata | Perform data-driven normalization | Removing systematic biases using a singular value decomposition-based method and preserving treatment effects with an ANOVA model | Data-driven normalization | (12) |
| linear baseline normalization | normalize_input_data_bydata | Perform data-driven normalization | Normalizing a sample by a scaling factor computed as the ratio of the mean intensity of the baseline to the sample mean | Data-driven normalization | (13) |
| Non-linear baseline normalization (Li-Wong) | normalize_input_data_bydata | Perform data-driven normalization | Baseline normalization assuming possible non-linear relationships between the baseline and the individual | Data-driven normalization | (14) |
| cyclic locally weighted regression (cyclic loess) | normalize_input_data_bydata | Perform data-driven normalization | Nonlinear normalization by comparing two samples, and using locally weighted regression for curve fitting | Data-driven normalization | (15) |
| mean | normalize_input_data_bydata | Perform data-driven normalization | Normalizing each sample by the mean value of its feature intensities | Data-driven normalization | (16) |
| median | normalize_input_data_bydata | Perform data-driven normalization | Normalizing each sample by the median value of its feature intensities | Data-driven normalization | (16) |
| probabilistic quotient normalization (PQN) | normalize_input_data_bydata | Perform data-driven normalization | Dividing all variables by the median of all quotients calculated from a sample and a reference | Data-driven normalization | (17) |
| quantile | normalize_input_data_bydata | Perform data-driven normalization | Normalization method that produces a similar distribution of feature intensities | Data-driven normalization | (13) |
| sum | normalize_input_data_bydata | Perform data-driven normalization | Normalizing each sample by the sum of its feature intensities | Data-driven normalization | (16) |
| variance stabilization normalization (VSN) | normalize_input_data_bydata | Perform data-driven normalization | Nonlinear normalization leading to equal variable variance | Data-driven normalization | (18) |
| auto scaling | scale_input_data | Perform data scaling | Scaling factor based on standard deviation making all metabolites become equally important and have a comparable scale; inflation of measurement errors; between-sample variation could not be corrected  $\frac{{(x}_{ij}-\bar{x_{i}})}{{sd}_{i}}$ | Data scaling | (19,20) |
| level scaling | scale_input_data | Perform data scaling | Scaling factor based on mean value suggested for studying large relative responses of a biological factor; inflation of measurement errors  $\frac{{(x}_{ij}-\bar{x_{i}})}{\bar{x_{i}}}$ | Data scaling | (19) |
| pareto scaling | scale_input_data | Perform data scaling | Scaling factor based on square root of standard deviation reducing weight of large values and keeping processed data closer to its original values; sensitive to large fold changes; very large fold changes still have a dominating effect  $\frac{{(x}_{ij}-\bar{x_{i}})}{\sqrt{{sd}_{i}}}$ | Data scaling | (19) |
| power scaling | scale_input_data | Perform data scaling | Pseudo scaling that can reduce heteroscedasticity and skewness; no rule for the choice of square root or cube root; multiplicative effects will not become additive; restriction on negative values  $\sqrt{x_{ij}}-\bar{\sqrt{x_{i}}}$ | Data scaling | (21) |
| range scaling | scale_input_data | Perform data scaling | Scaling factor based on difference between minimal and maximal intensities of a feature related to biology; Comparing metabolites relative to the response range making all metabolites become equally important; sensitive to outliers  $\frac{{(x}_{ij}-\bar{x_{i}})}{(\max_{} x_{i}-\min_{} x_{i})}$ | Data scaling | (19) |
| vast scaling | scale_input_data | Perform data scaling | Extension of auto scaling based on standard deviation and the coefficient of variation useful for metabolites with small fluctuations; require group structure for classification and biomarker identification  $\frac{{(x}_{ij}-\bar{x_{i}})}{{sd}_{i}}\times\frac{\bar{x_{i}}}{{sd}_{i}}$ | Data scaling | (19) |
| cube root transformation (cube) | transform_input_data | Perform data transformation | Pseudo scaling that can reduce heteroscedasticity and right skewness; differences of scaling effects between large and small values; transformation is weaker than the logarithm and the cube root  $\sqrt[3]{x_{ij}}$ | Data transformation | (21) |
| logarithm transformation, base 2 and 10 (log2, log10) | transform_input_data | Perform data transformation | Pseudo scaling that can reduce heteroscedasticity and right skewness; differences of scaling effects between large and small values; restriction on zero and negative values; problem  on values with a large relative standard deviation  $\log_{2} or \log_{10} (x_{ij})$ | Data transformation | (19) |
| generalized log transformation, base 2 and 10 (glog2, glog10) | transform_input_data | Perform data transformation | Pseudo scaling that can reduce heteroscedasticity and right skewness, and stabilize variance; differences of scaling effects between large and small values  $\log_{2}{or \log}_{10} \left( \frac{{(x}_{ij}+\sqrt{x_{ij}^{2}+{\min_{} X}^{2}})}{2} \right)$ | Data transformation | (16,22) |
| square root transformation (sqrt) | transform_input_data | Perform data transformation | Pseudo scaling that can reduce heteroscedasticity and right skewness; differences of scaling effects between large and small values; restriction on negative value; transformation is weaker than the logarithm and the cube root  $\sqrt{x}_{ij}$ | Data transformation | (19) |

The $i^{th}$ metabolite of the $j^{th}$sample represented as $x_{ij}$; $\bar{x_{i}}$ is the mean intensity; ${sd}_{i}$ is the standard deviation; $X$ is the matrix of all metabolite intensities.

**Table S2.** List of univariate analysis in the metabox 2.0

|  | **Pairwise analysis** | | **1-way ANOVA** | | **2-way ANOVA** | | **Correlation** | **Multi-level/non-independent** |
| --- | --- | --- | --- | --- | --- | --- | --- | --- |
|  | **Independent** | **Repeated** | **Independent** | **Repeated** | **Independent** | **Repeated** |  |  |
| **Parametric** | Student's t-test | Paired t-test | ANOVA -> Tukey's HSD | ANOVA -> Pairwise t-test | ANOVA -> Tukey's HSD | ANOVA -> Pairwise t-test | Pearson | Linear mixed-effects model |
| **Non-parametric** | Mann-Whitney U test | Wilcoxon signed-rank test | Kruskal-Wallis rank sum test -> Dunn’s test | Friedman rank sum test -> Pairewise Wilcoxon signed-rank test | Scheirer Ray Hare test -> Pairewise Mann-Whitney U test | - | Spearman | - |

**Reference**

1. Kokla, M., Virtanen, J., Kolehmainen, M., Paananen, J., and Hanhineva, K. (2019) Random forest-based imputation outperforms other methods for imputing LC-MS metabolomics data: a comparative study. *BMC Bioinformatics* **20**, 492

2. Breiman, L. (2001) Random Forests. *Machine Learning* **45**, 5-32

3. Hastie, T., Tibshirani, R., Narasimhan, B., and Chu, G. (2023) impute: impute: Imputation for microarray data. 1.72.3 Ed., R package

4. Stacklies, W., Redestig, H., Scholz, M., Walther, D., and Selbig, J. (2007) pcaMethods—a bioconductor package providing PCA methods for incomplete data. *Bioinformatics* **23**, 1164-1167

5. Redestig, H., Fukushima, A., Stenlund, H., Moritz, T., Arita, M., Saito, K., and Kusano, M. (2009) Compensation for systematic cross-contribution improves normalization of mass spectrometry based metabolomics data. *Anal Chem* **81**, 7974-7980

6. Sysi-Aho, M., Katajamaa, M., Yetukuri, L., and Orešič, M. (2007) Normalization method for metabolomics data using optimal selection of multiple internal standards. *BMC Bioinformatics* **8**, 93

7. De Livera, A. M., Sysi-Aho, M., Jacob, L., Gagnon-Bartsch, J. A., Castillo, S., Simpson, J. A., and Speed, T. P. (2015) Statistical methods for handling unwanted variation in metabolomics data. *Anal Chem* **87**, 3606-3615

8. Fan, S., Kind, T., Cajka, T., Hazen, S. L., Tang, W. H. W., Kaddurah-Daouk, R., Irvin, M. R., Arnett, D. K., Barupal, D. K., and Fiehn, O. (2019) Systematic Error Removal Using Random Forest for Normalizing Large-Scale Untargeted Lipidomics Data. *Anal Chem* **91**, 3590-3596

9. Cleveland, W. S., and Devlin, S. J. (1988) Locally Weighted Regression: An Approach to Regression Analysis by Local Fitting. *Journal of the American Statistical Association* **83**, 596-610

10. Åstrand, M. (2003) Contrast Normalization of Oligonucleotide Arrays. *Journal of Computational Biology* **10**, 95-102

11. Workman, C., Jensen, L. J., Jarmer, H., Berka, R., Gautier, L., Nielser, H. B., Saxild, H.-H., Nielsen, C., Brunak, S., and Knudsen, S. (2002) A new non-linear normalization method for reducing variability in DNA microarray experiments. *Genome Biology* **3**, research0048.0041

12. Karpievitch, Y. V., Nikolic, S. B., Wilson, R., Sharman, J. E., and Edwards, L. M. (2014) Metabolomics data normalization with EigenMS. *PLoS One* **9**, e116221

13. Bolstad, B. M., Irizarry, R. A., Astrand, M., and Speed, T. P. (2003) A comparison of normalization methods for high density oligonucleotide array data based on variance and bias. *Bioinformatics* **19**, 185-193

14. Li, C., and Hung Wong, W. (2001) Model-based analysis of oligonucleotide arrays: model validation, design issues and standard error application. *Genome Biol* **2**, RESEARCH0032

15. Emwas, A. H., Saccenti, E., Gao, X., McKay, R. T., Dos Santos, V., Roy, R., and Wishart, D. S. (2018) Recommended strategies for spectral processing and post-processing of 1D (1)H-NMR data of biofluids with a particular focus on urine. *Metabolomics* **14**, 31

16. Pang, Z., Chong, J., Li, S., and Xia, J. (2020) MetaboAnalystR 3.0: Toward an Optimized Workflow for Global Metabolomics. *Metabolites* **10**

17. Dieterle, F., Ross, A., Schlotterbeck, G., and Senn, H. (2006) Probabilistic Quotient Normalization as Robust Method to Account for Dilution of Complex Biological Mixtures. Application in 1H NMR Metabonomics. *Analytical Chemistry* **78**, 4281-4290

18. Huber, W., von Heydebreck, A., Sültmann, H., Poustka, A., and Vingron, M. (2002) Variance stabilization applied to microarray data calibration and to the quantification of differential expression. *Bioinformatics* **18**, S96-S104

19. van den Berg, R. A., Hoefsloot, H. C., Westerhuis, J. A., Smilde, A. K., and van der Werf, M. J. (2006) Centering, scaling, and transformations: improving the biological information content of metabolomics data. *BMC Genomics* **7**, 142

20. Kohl, S. M., Klein, M. S., Hochrein, J., Oefner, P. J., Spang, R., and Gronwald, W. (2012) State-of-the art data normalization methods improve NMR-based metabolomic analysis. *Metabolomics* **8**, 146-160

21. Yang, Q., Wang, Y., Zhang, Y., Li, F., Xia, W., Zhou, Y., Qiu, Y., Li, H., and Zhu, F. (2020) NOREVA: enhanced normalization and evaluation of time-course and multi-class metabolomic data. *Nucleic Acids Res* **48**, W436-W448

22. Rocke, D. M., and Durbin, B. (2003) Approximate variance-stabilizing transformations for gene-expression microarray data. *Bioinformatics* **19**, 966-972
