# Supplementary figures and images for: Data processing solutions to render metabolomics more quantitative: case studies in food and clinical metabolomics using Metabox 2.0
Source: Gigascience. 2024 Mar 15;13:giae005. doi: 10.1093/gigascience/giae005 (PMC10941642; doi:10.1093/gigascience/giae005)

(A) Data processing and analysis

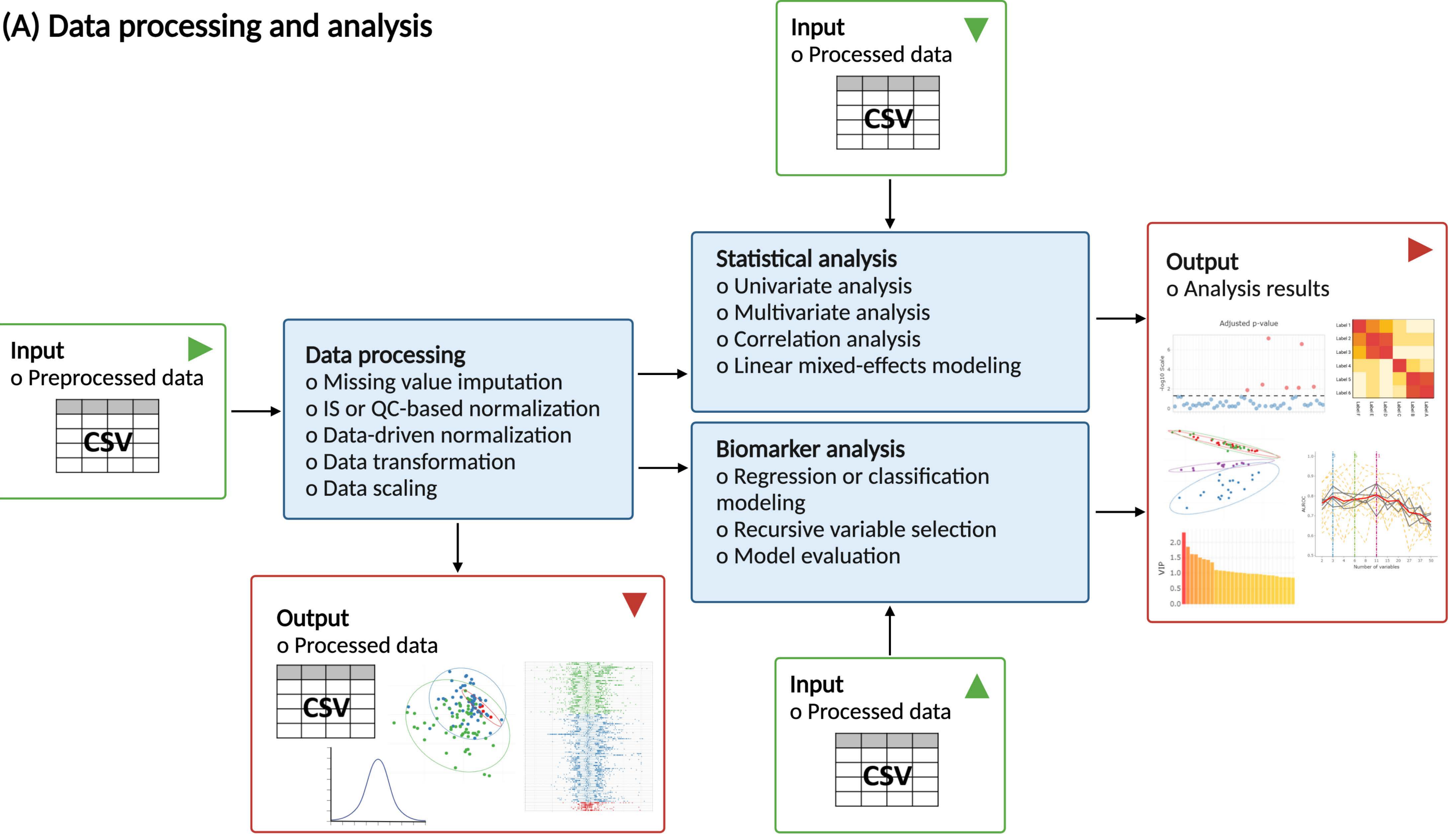

(B) Data integration

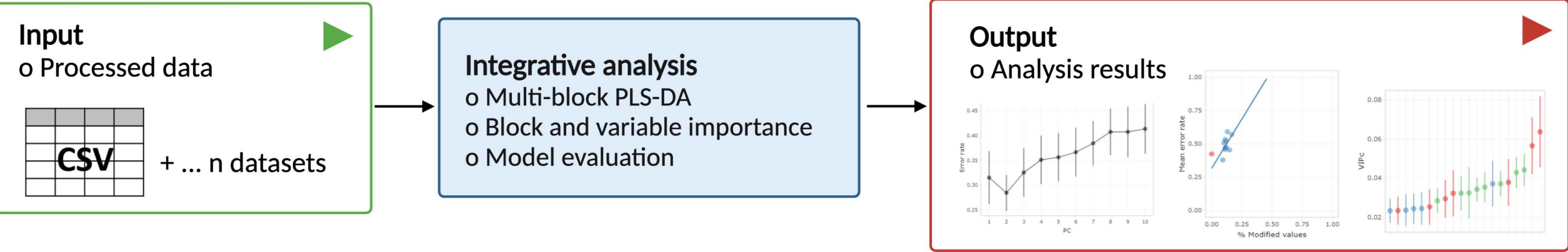

(C) Data interpretation

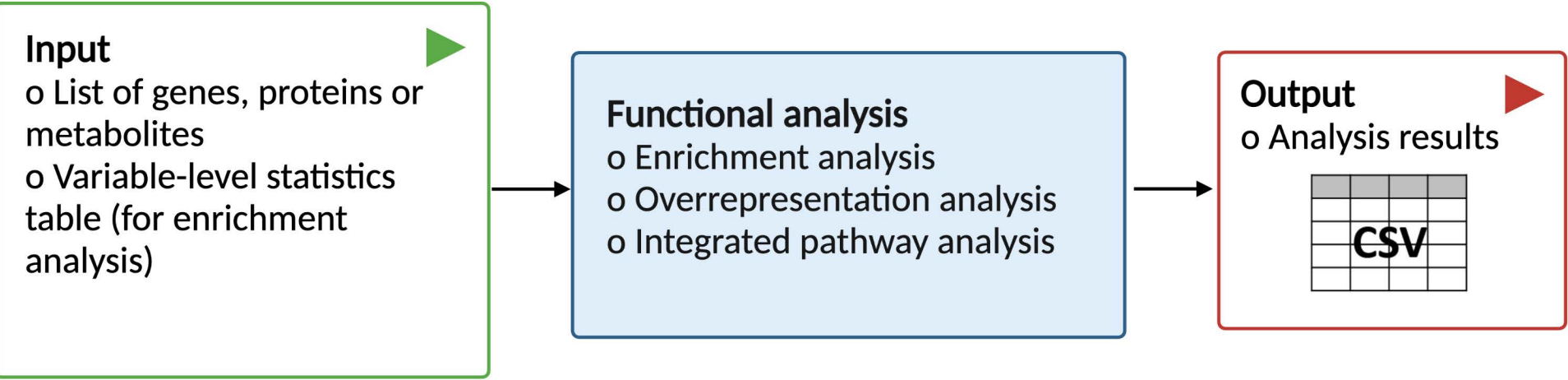

Supplement: giae005_Supplemental_Files [file giae005_supplemental_files.zip › R1_FigureS1.pdf]

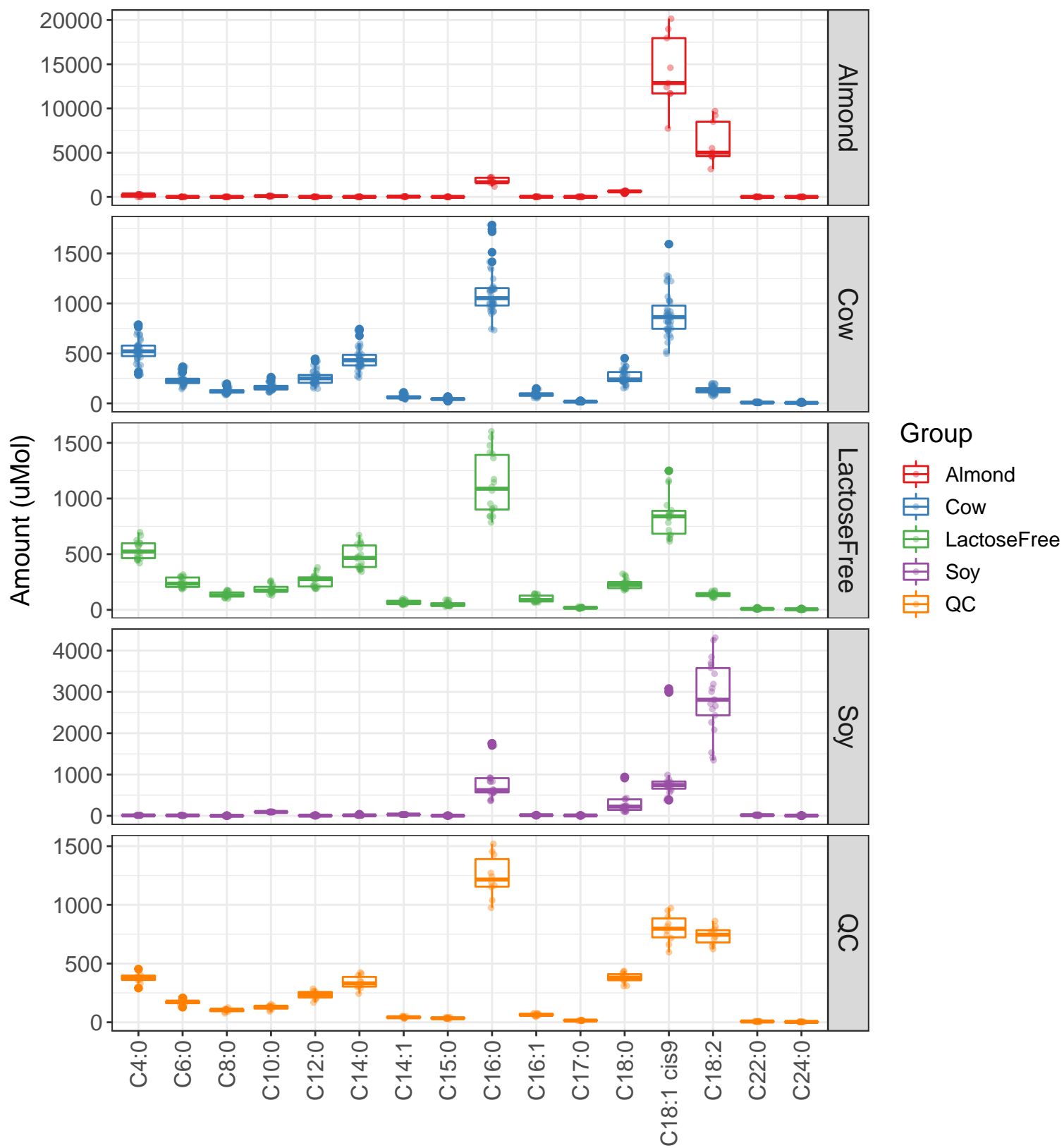

Supplement: giae005_Supplemental_Files [file giae005_supplemental_files.zip › R1_FigureS2.pdf]

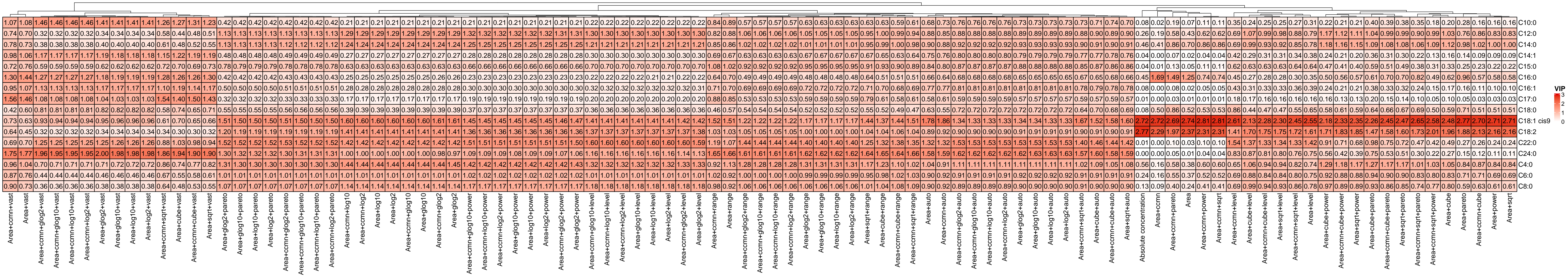

Supplement: giae005_Supplemental_Files [file giae005_supplemental_files.zip › R1_FigureS3.pdf]

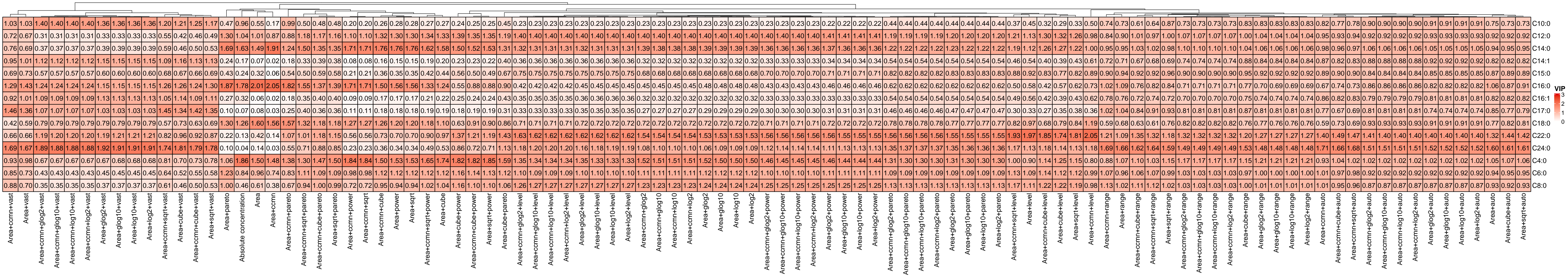

Supplement: giae005_Supplemental_Files [file giae005_supplemental_files.zip › R1_FigureS5.pdf]

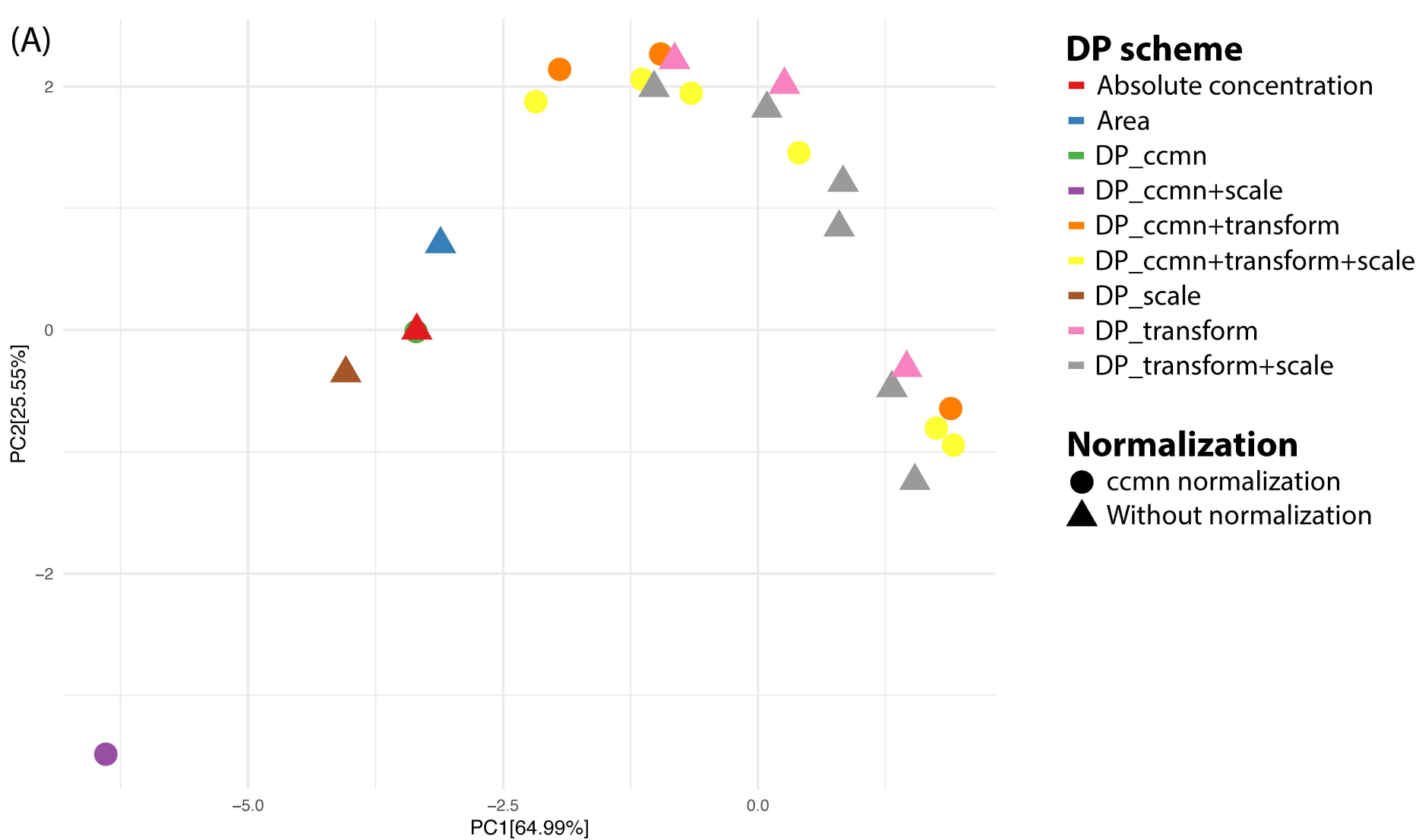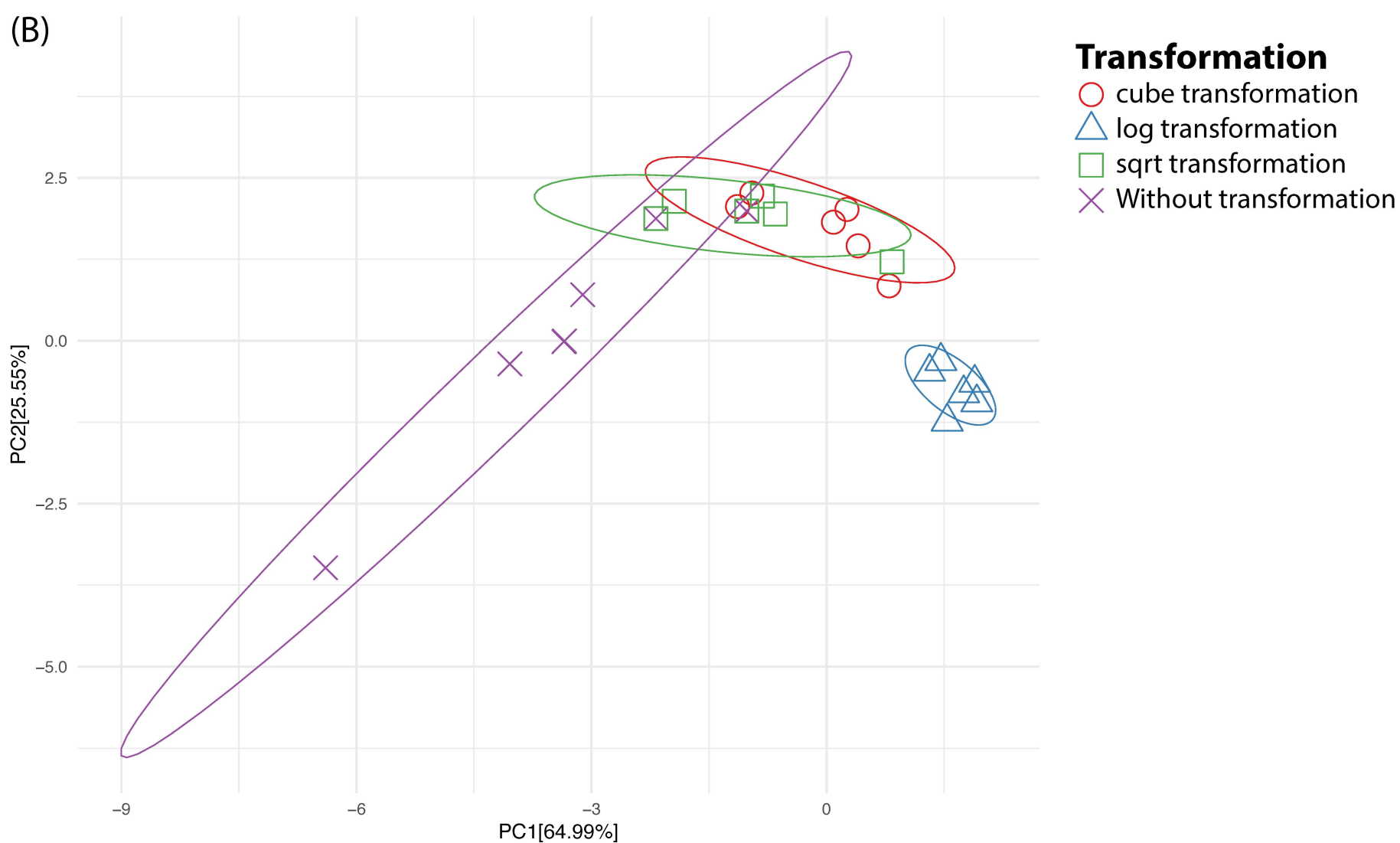

Supplement: giae005_Supplemental_Files [file giae005_supplemental_files.zip › R1_FigureS7.pdf]

(A)

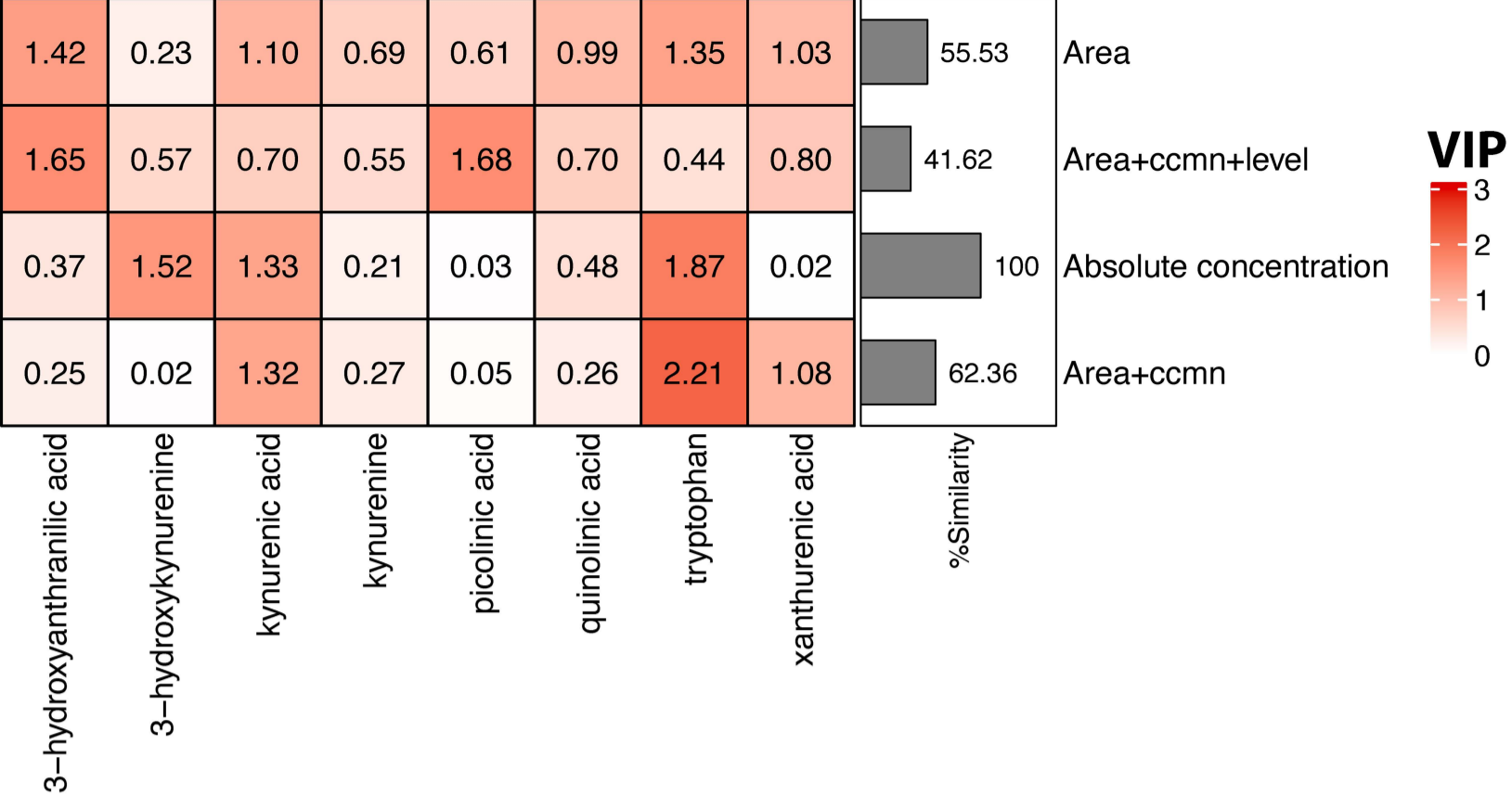

(B)

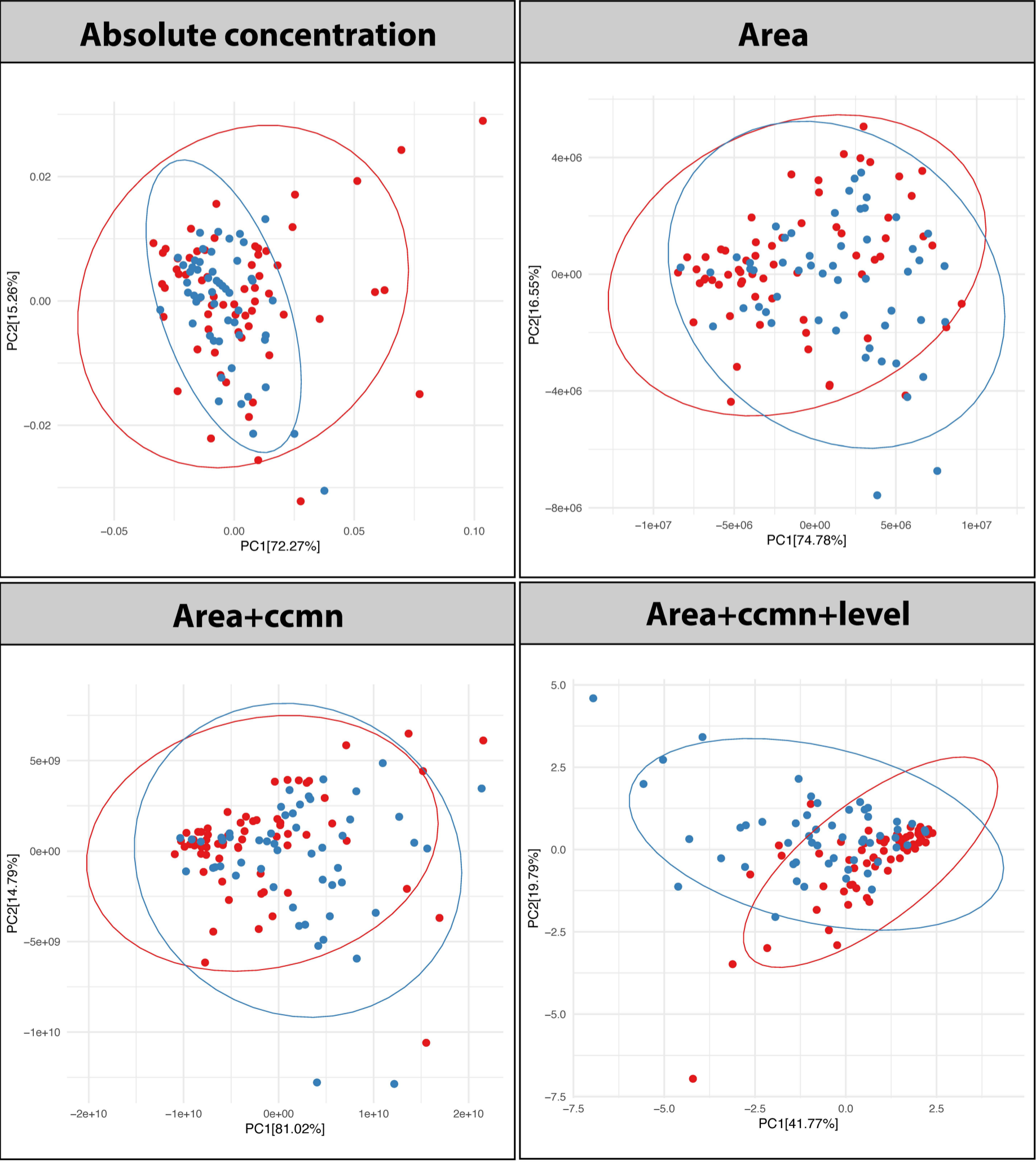

Group

LN

N

(C)

Absolute concentration

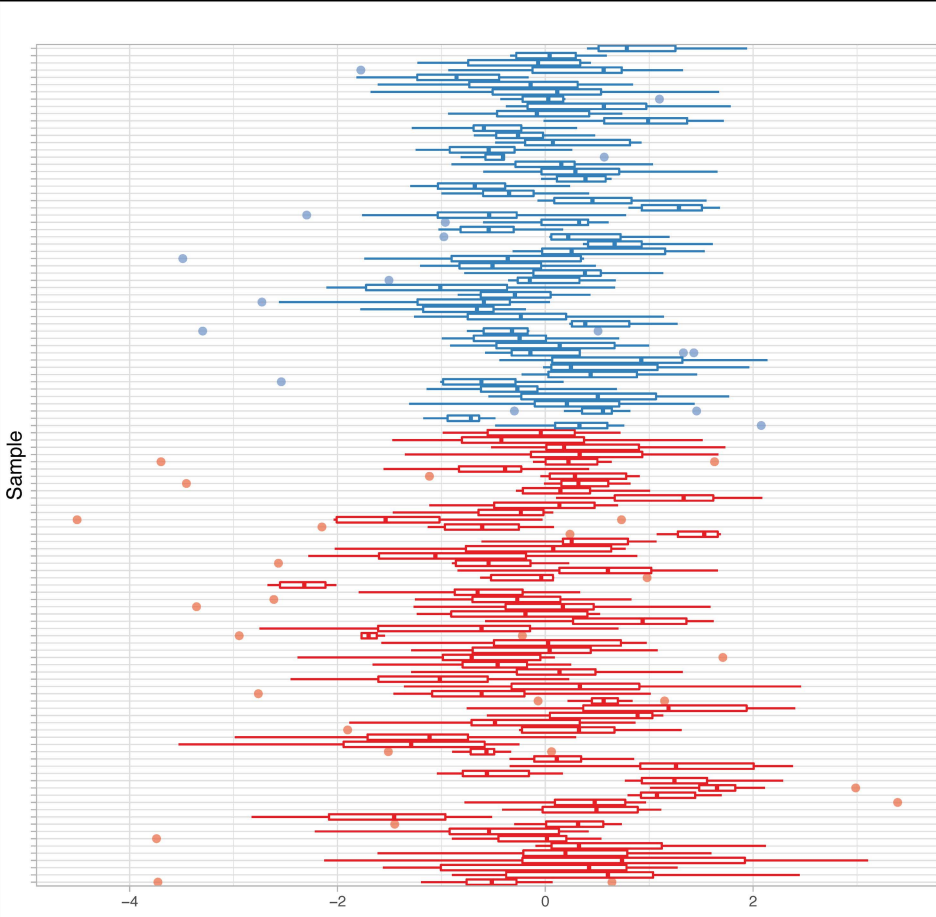

Area

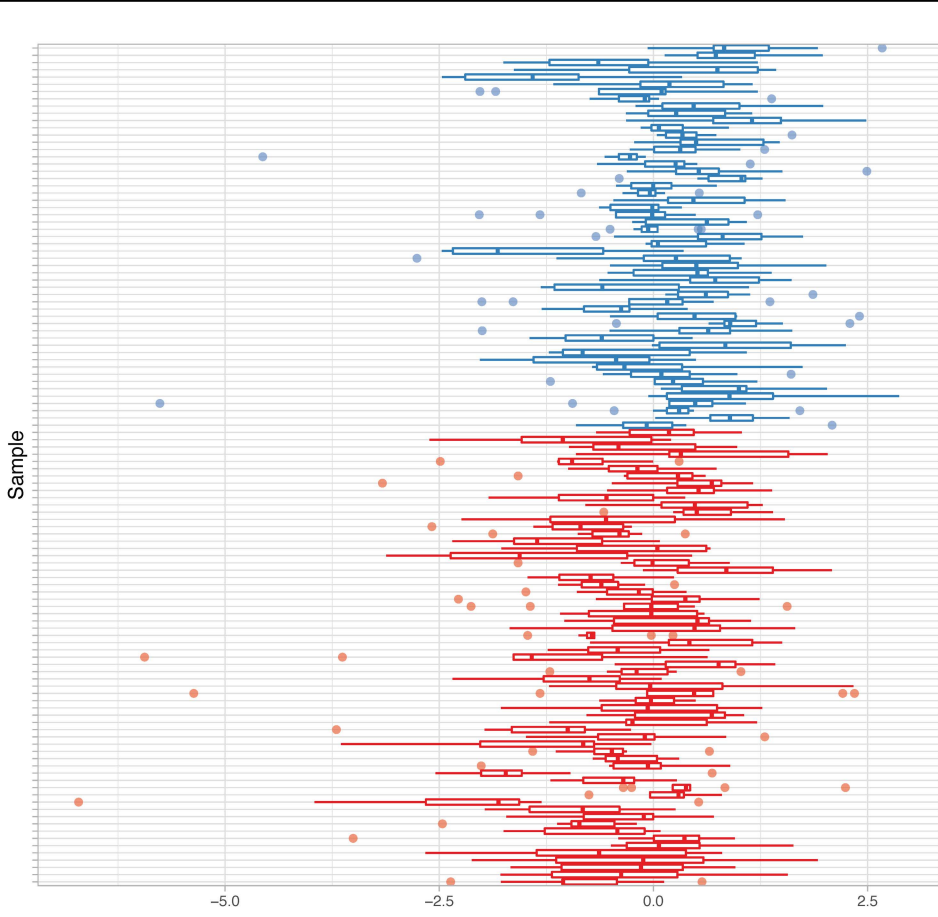

Area+ccmn

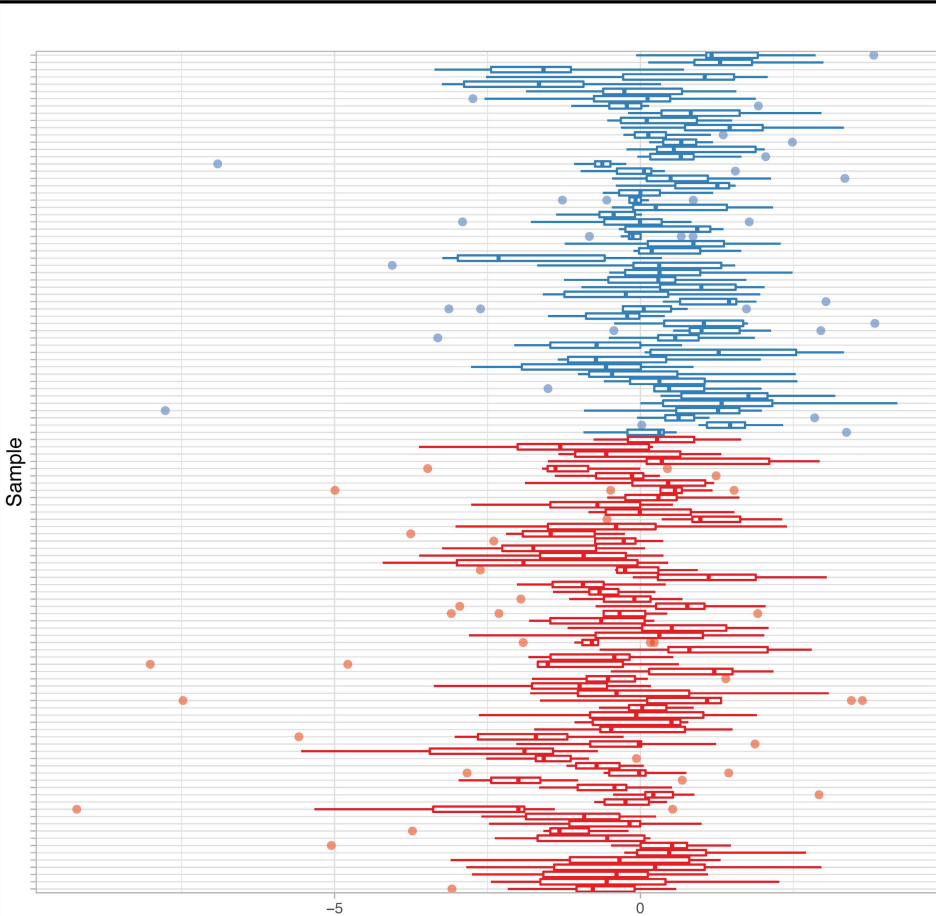

Area+ccmn+level

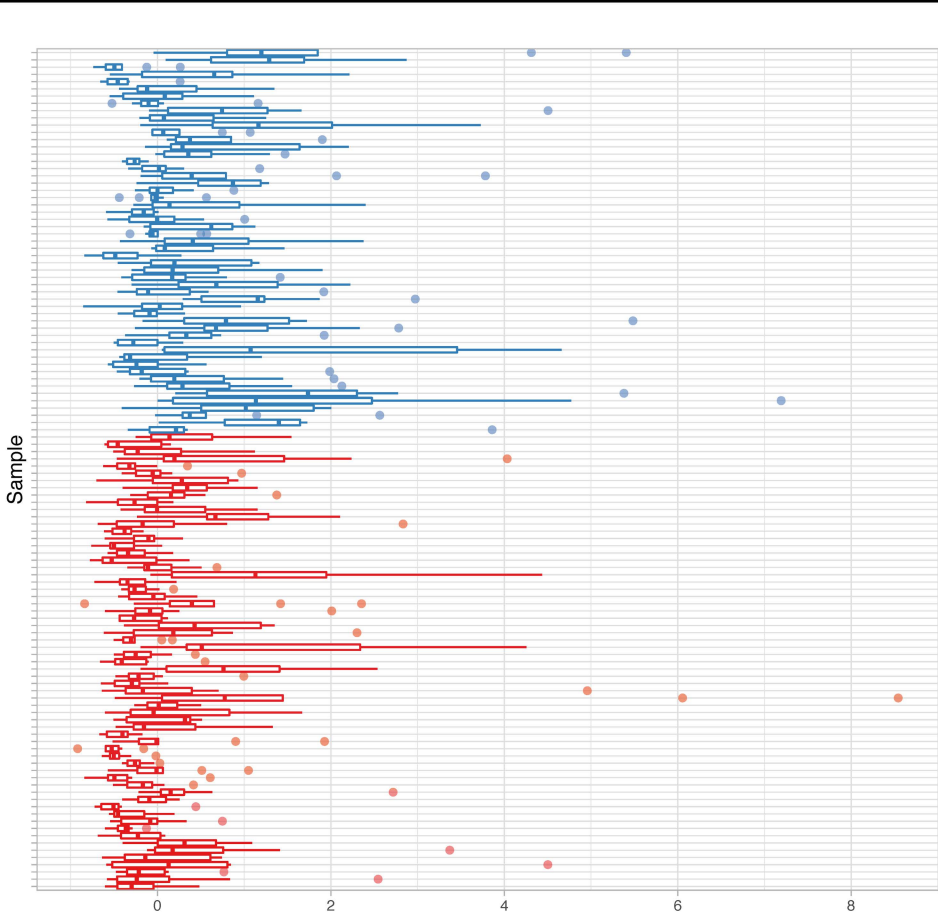

Group

LN

N

(D)

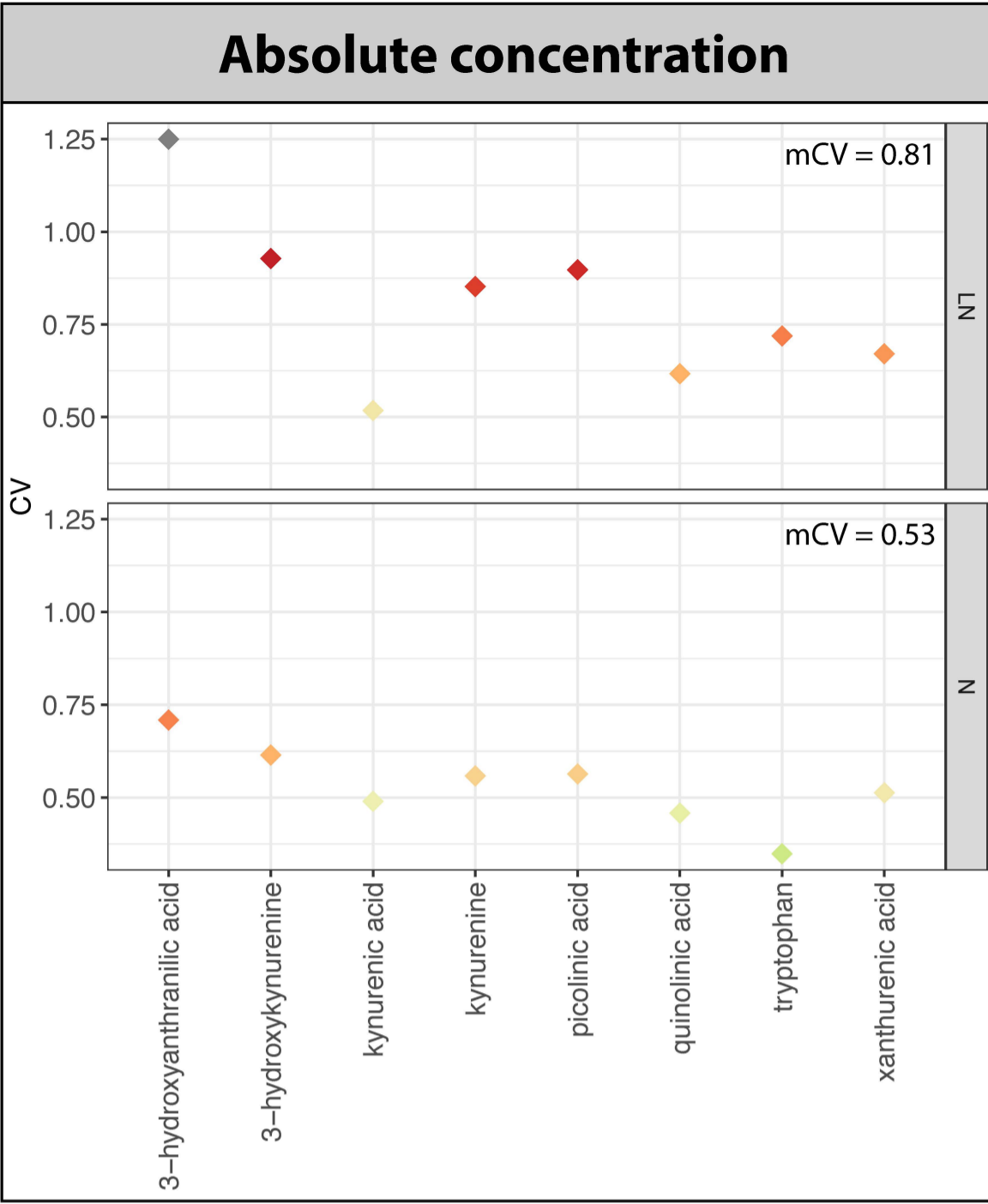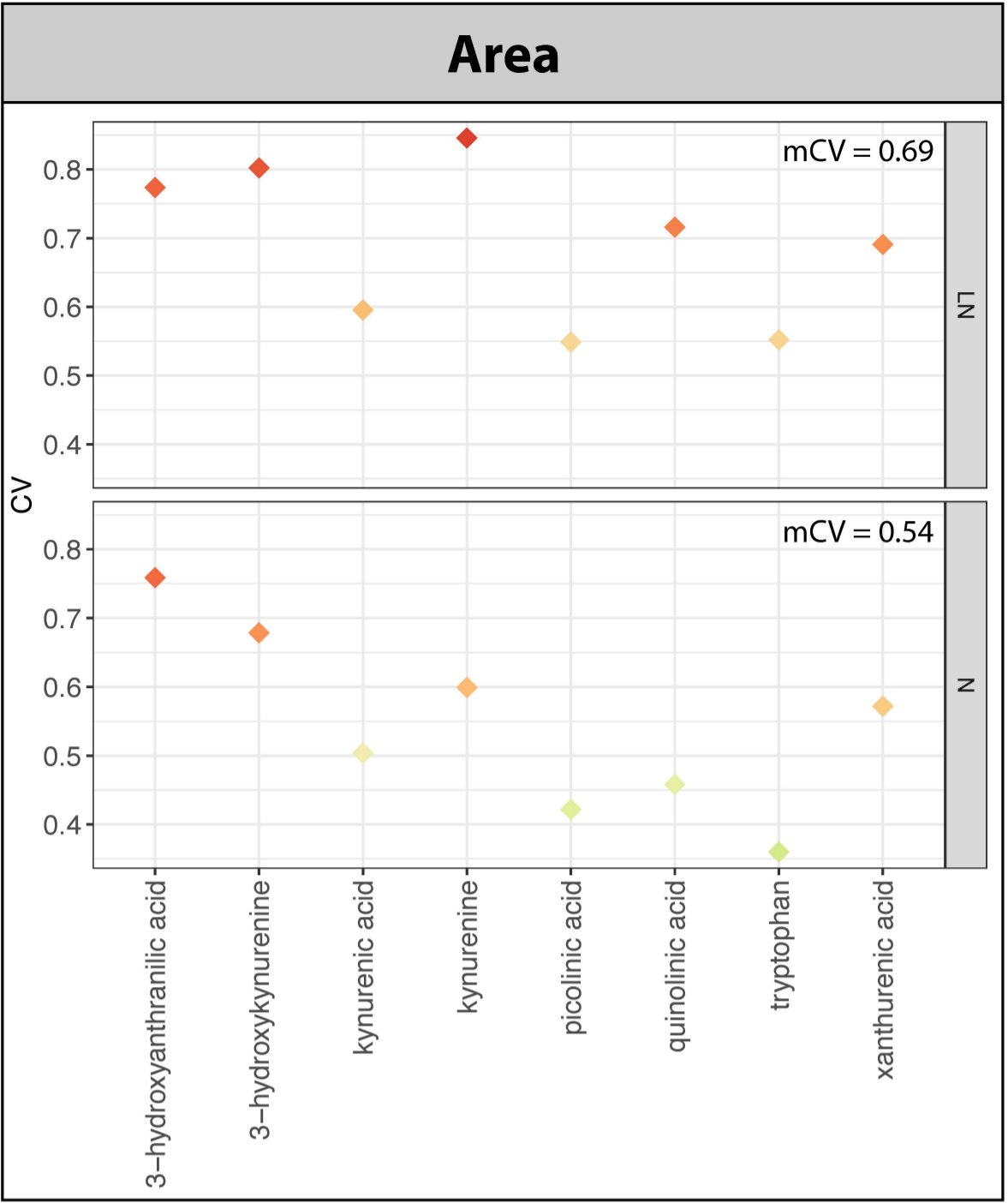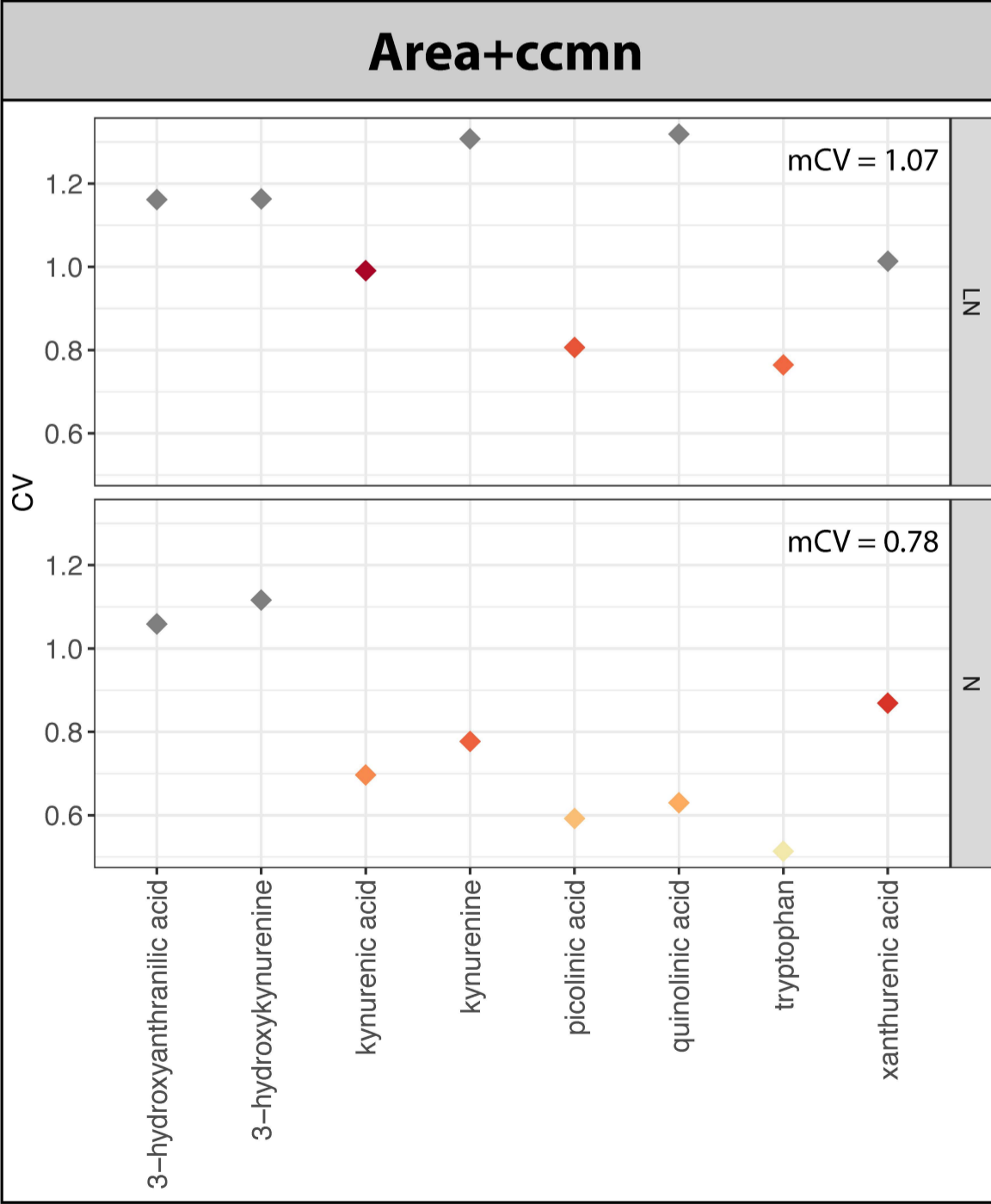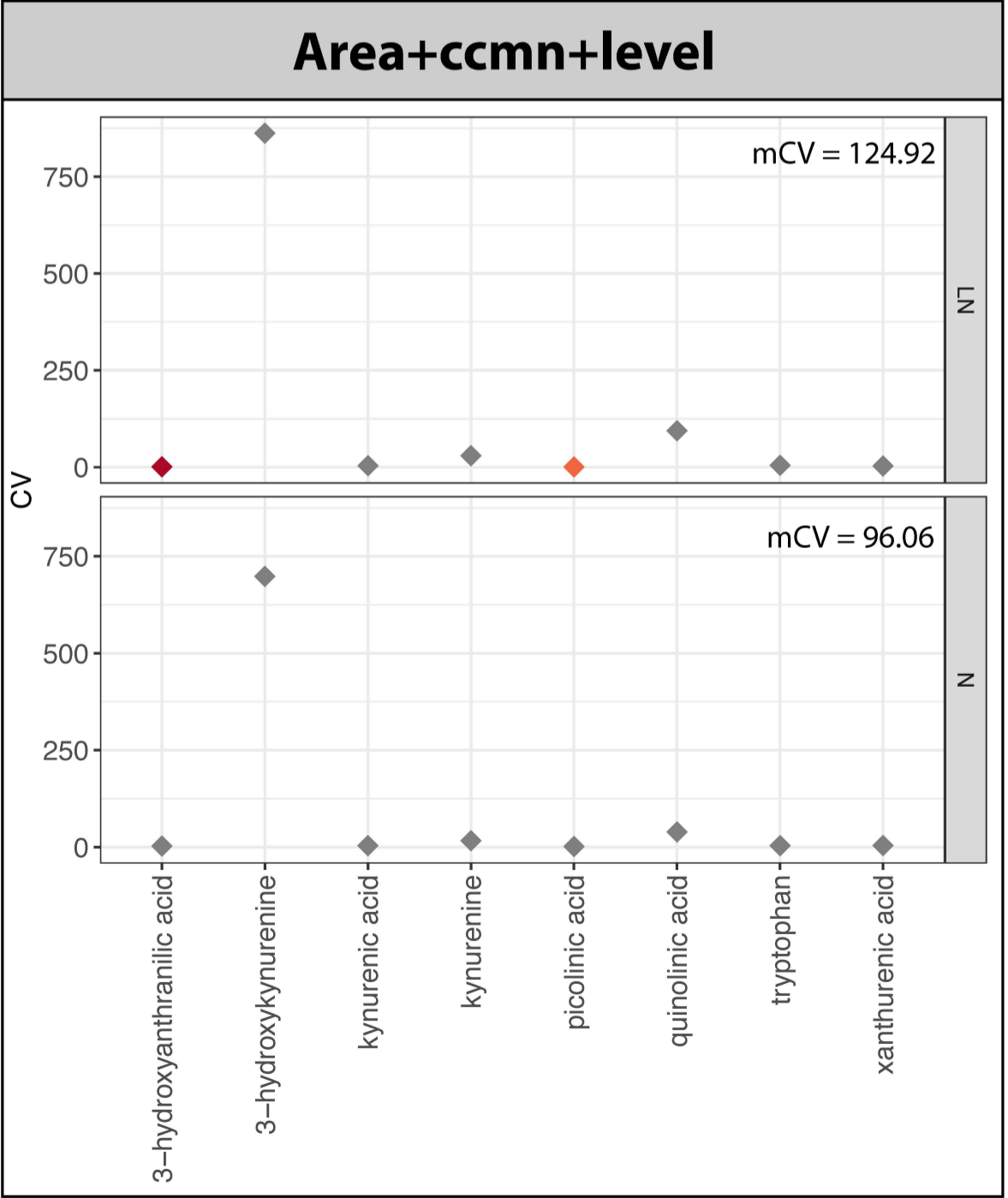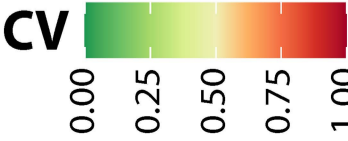

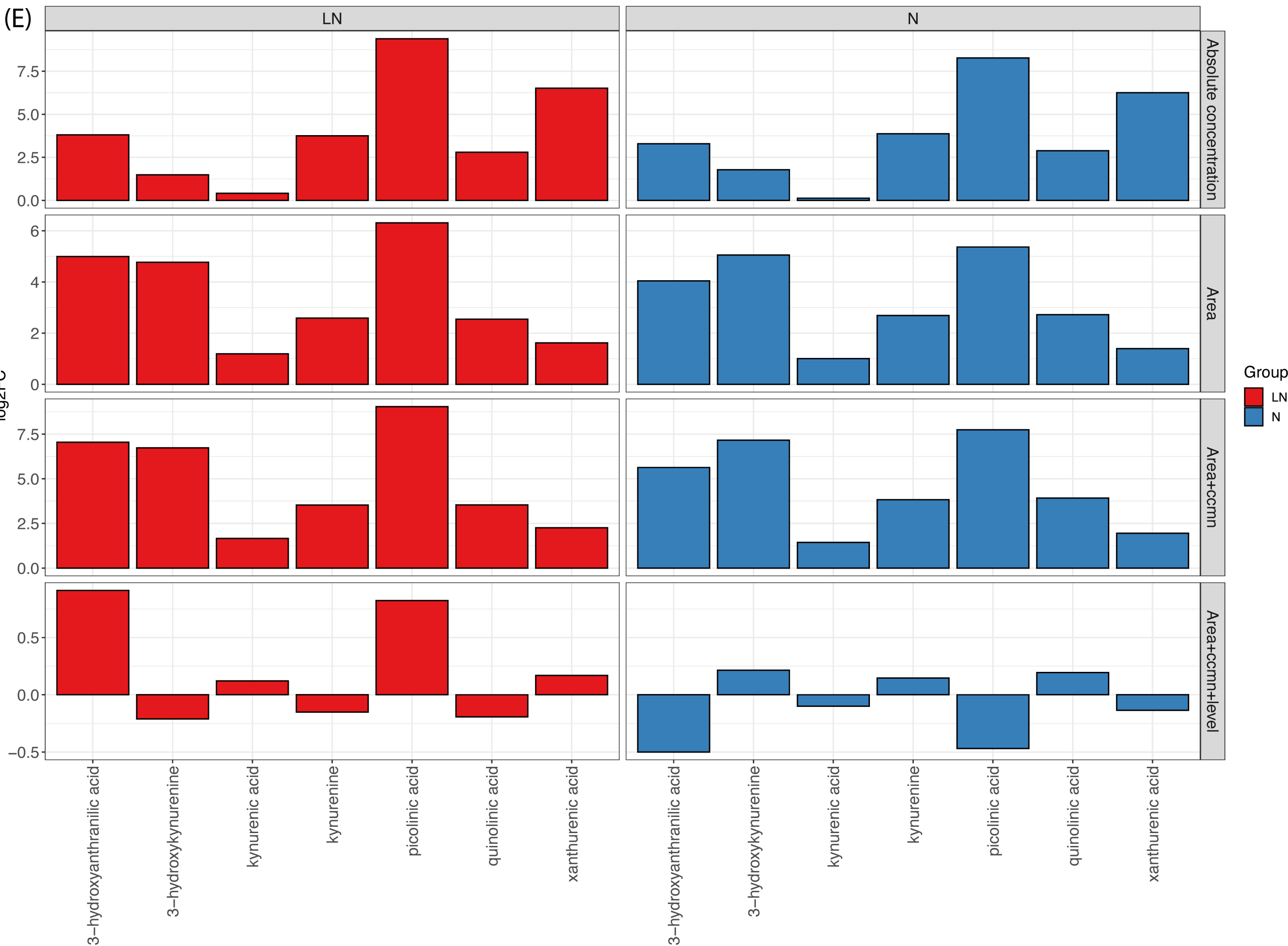

Supplement: giae005_Supplemental_Files [file giae005_supplemental_files.zip › R1_FigureS9.pdf]
