# Supplementary material for: Data processing solutions to render metabolomics more quantitative: case studies in food and clinical metabolomics using Metabox 2.0
Source: Gigascience. 2024 Mar 15;13:giae005. doi: 10.1093/gigascience/giae005 (PMC10941642; doi:10.1093/gigascience/giae005)
Supplement: giae005_Response_to_Reviewer_Comments_Original_Submission [file giae005_response_to_reviewer_comments_original_submission.pdf]

Author's Response To Reviewer Comments

Reviewer reports:

Reviewer #1: Wanichthanarak et al. present a benchmarking study of different data processing methods and their combinations. More than 90 method combinations which cover different normalization, transformation and scaling methods were evaluated using two metabolomics datasets. The performance of methods was evaluated primarily based on the comparison to the absolute concentration data. A major difference between this study and previous benchmarking studies is the utilization of absolute concentration data as a reference point for method evaluation. The results indicate that the cross-contribution compensating multiple standard normalization combined with square root data transformation was most appropriate for well-controlled studies. The authors also present a new version of Metabox (version 2) in the manuscript. Overall, the manuscript is well-written, and the study is of interest.

Answer: Thank you very much for your positive comments on our manuscript. We highly appreciate your time and consideration for our manuscript.

Minor comments:
1. How cross-contribution compensating multiple standard normalizations is performed? Details should be described in the manuscript.

Answer: We thank the reviewer for this comment. Accordingly, we have clarified this point in 'Analysis workflow' section as follows:
The ccmn normalization was performed using the normalize_input_data_byqc function from the R package Metabox 2.0. The function was implemented from the CRMN R package for normalization of metabolomics data [22]. Heptanoic methyl ester and anthranilic acid C13 were used as an IS in study I and study II, respectively. Known amounts of ISs were added to samples before sample preparation, so that metabolite peak areas were normalized with respect to the responses of the ISs.

2. What are the differences among Metabox 2.0, Metabox 1.0 and other commonly used pipelines in metabolomics? It will be useful to add a table to show the features of these different tools.

Answer: We thank the reviewer for this comment. We have provided comparison of Metabox 2.0, Metabox 1.0, and other tools in a Table S8.

3. The manuscript is mainly about the benchmarking of different data processing methods. However, the title of the manuscript seems to indicate that the manuscript is focused on the development of a new version of a data processing tool (Metabox 2.0). To better align with the actual content, I'd like to suggest the authors change the title to accurately reflect the primary focus of the manuscript.

Answer: We thank the reviewer for this comment. We have changed the title to 'Data processing solutions to render metabolomics more quantitative: case studies in food and clinical metabolomics using Metabox 2.0'. We would like to keep the word "Metabox" in there because we have been working to develop this tool since the last version. This will keep current users updated on new features and attract other potential users.

4. Page 13 - Effects on data properties: "For each metabolite, we considered …" should be "For each DP scheme, we considered …"?

Answer: We thank the reviewer for pointing out this point. This point has been fixed.

Reviewer #2: The manuscript titled "Metabox 2.0: The data processing solution that renders metabolomics more quantitative" provides a comprehensive evaluation of data processing methods in the context of metabolomics, with a focus on achieving results closely aligned with absolute concentrations. The authors address a crucial aspect in metabolomics research, particularly in distinguishing appropriate processing

procedures for a given experimental setup.

While the manuscript provides a comprehensive analysis of the impact of different data processing methods on semi-quantified metabolites, some critical questions need to be addressed before publication to improve the clarity of the method and to prove the robustness of the method.

1. While PCA plots are informative, the interpretation could be enhanced by explicitly stating the biological relevance of observed separations or clustering, such as enrichment analysis. A more detailed discussion on the biological implications of the observed patterns in the PCA plots would provide additional context.

Answer: We thank the reviewer for this comment. Although, biological implications are not the main focus of this manuscript, we have improved this point in 'Effects on multivariate analysis' section as follows:
Since C18:1 cis9 and C18:2n-6 were the important plant UFAs, a clustering of plant-based milk products was observed. Meanwhile, the whole and lactose-free bovine milk were clustered together because their FA profiles were similar (Figure S2). The key distinction between these bovine milk types was the absence of lactose in lactose-free bovine milk [18].

And in 'Effects on multivariate analysis in the absence of highly abundant metabolites' section as follows:
Even without the plant UFAs, the clustering of plant-based and bovine milk types persisted. This was because of the differences in FA compositions as reported by Jariyasopit et al., [18]. Both bovine milk types were more enriched with saturated FA compared to plant-based milk products (Figure S2).

And in 'Data processing effects on the semi-quantified metabolites in urine samples' section as follows:
However, the clustering of different subject groups was largely due to tryptophan (Figure S9A), This metabolite was reported as a potential biomarker for chronic kidney diseases [17].

2. While the study effectively compares various DP methods, a critical examination of the performances, limitations, and assumptions of each method could further inform the readers, in the form of tables or figures. Discussing the potential biases introduced by certain methods or scenarios where specific methods might not be suitable would contribute to a more balanced evaluation.

Answer: We thank the reviewer for this comment. We have included the principles, equations, and limitations of the DP methods in Table S1.
In 'Discussion' section, we provided discussion on the performance of vast and level scaling based on different characteristics of milk and urine data as follows:
The vast-scaled milk and level-scaled urine produced the most divergence among VIP results from the CONC data. This is due to the fact that vast scaling is more suitable for data sets with small induced fluctuations [11, 24], which is not the case in this study. The FAs with a large variation were considered less important, while a low-deviated metabolite became more significant after vast scaling. In contrast to level scaling, this approach is suggested for a study that involves large relative responses of a biological factor [11, 24]. This method failed when using the urine sample data because the signal-to-noise ratio was low.

And we have discussed more on the performance of log transformation as follows:
Transformation by log family is a commonly used approach in omic data analysis. Its transformation is stronger than cube and sqrt methods (i.e., transformed data is more divergent from the original). The log transformation performs well for data with constant relative standard deviation [24]. However, it is not always the case in metabolomics, where variance gets larger with an increasing intensity level. The log transformation tends to reduce the large variance for large values, but it rather inflates the variance of metabolites close to zero [50]. In this study, its performance was modest for both milk and urine sample data sets (VIP similarity = 35-45%). By using the log transformation, one needs to balance the trade-off between obtaining more discriminant metabolites and gaining more false positives.

3. The decision to exclude major metabolites is justified, but a more extensive discussion on the potential consequences and limitations of this choice would add depth. Addressing how excluding major metabolites may impact the overall conclusions or generalizability of the findings is essential. In another word, how

robustness the method would be by only comparing quantified and semi-quantified FAs?

Answer: We thank the reviewer for this comment. As previously discussed, metabolites present in high concentrations might influence discriminant analysis. In our study, the plant UFAs (C18:1 cis-9 and C18:2) have very high concentrations compared to the other FAs. We, therefore, demonstrated this point by using data sets with C18:1 cis-9 and C18:2, and without both FAs. We have clarified this point in 'Effects on multivariate analysis in the absence of highly abundant metabolites' section as follows:
The absolute amounts of C18:1 cis-9 and C18:2 were relatively high compared to those of the other FAs (Figure S2). They were the main discriminants between almond milk, soymilk, and plant-based and bovine milk in the PLS-DA (Figure 3). The previous section showed a case study involving variables with strong relative responses of a biological factor (milk types). We continued our evaluation of the milk data set, excluding the major metabolites C18:1 cis-9 and C18:2. This was to represent a case study without extreme relative responses.

And we have provided more discussion in 'Discussion' section as follows:
Class separation in multivariate models such as PCA, and PLS-DA is attributed to metabolites with high loadings, which are usually proportional to the concentration or magnitude of fold change [24, 50, 51]. These dominant sources of variation could be informative markers or obscure dominators. We showed that, with the presence of very high concentrations of plant UFAs (C18:1 cis-9 and C18:2), they were always the main discriminants between almond milk, soymilk, and plant-based and bovine milk. Without both plant UFAs, the second most abundant FAs (C16:0, C4:0, and C14:0) became the key source of variation between plant-based and bovine milk types. However, vast, range, and auto greatly minimized the importance of plant UFAs and resulted in a higher loading for metabolites with low measured levels. Different transformation and scaling methods adjust scale-size effects to a certain degree, allowing low-concentration metabolites to be more important [24]. If a low-abundant metabolite could reflect the variation of interest, it is worth considering the separation of very high-abundant metabolites to enable exploration of low-concentration regions.

4. The study focuses on the semi-quantitative analysis and its alignment with quantitative data. However, a critical discussion on the inherent limitations and potential biases introduced by semi-quantitative methods compared to absolute quantification would be valuable.

Answer: We thank the reviewer for this comment. We have discussed these issues and added them in 'Discussion' section.
In the field of metabolomics, quantitative analysis is important for understanding cellular metabolism because the abundances of metabolites affect both free energy and metabolic reactions [47]. Nonetheless, a number of obstacles, including the absence of standardized methods and the accessibility of reference standards, lead the majority of metabolomics research to be conducted using a semi-quantitative analysis. Although semi-quantitative analysis does not provide the true value of a metabolite's concentration, it is still useful for the discovery of important metabolites in many studies [3]. Often, the potential bias or technical errors introduced by this approach can be removed or minimized through techniques in analytical chemistry and bioinformatics [2, 7]. The major limitation of semi-quantitative compared to quantitative analysis is that it is challenging to compare results across different studies, leading to difficulty translating the potential metabolites into practice, especially in clinical research [48].

5. The study could benefit from a sensitivity analysis, exploring how the results might vary under different conditions or datasets. Examining the robustness of conclusions by introducing existing pairs of quantified and semi-quantified dataset.

Answer: We thank the reviewer for this comment. We agree with this comment. Unfortunately, most studies either provide quantitative or semi-quantitative data, there is a very limited number of both semi-quantified data and its quantitative companion publicly available for comparison. In this study, we used our own data sets, which were recently published with rigorous method validation. We assure that the use of milk and urine sample data sets could represent common scenarios in metabolomics, including a data set for studying the relative responses of a biological factor and a data set with a complex matrix, as stated in the Discussion section. We believe that our findings and method evaluation strategies will be a helpful resource for the metabolomic community. However, we are welcome any further comments or suggestions.

6. English need to be significantly improved.

Answer: We thank the reviewer for this comment. The manuscript was proofread by SAGE Author Services prior to submission. This revised manuscript was also proofread by Dr. Jonathan Robinson as stated in 'Acknowledgments' section.

In summary, the study's approach to evaluating DP methods with respect to quantitative data serves as a novel and valuable contribution to the field. However, the generalizability and the robustness of Metabox 2.0 need to be answered before publication.

Answer: Thank you very much for this comment. We appreciate your time and the time you have to spend on this manuscript.
