# Supplementary material for: Data processing solutions to render metabolomics more quantitative: case studies in food and clinical metabolomics using Metabox 2.0
Source: Gigascience. 2024 Mar 15;13:giae005. doi: 10.1093/gigascience/giae005 (PMC10941642; doi:10.1093/gigascience/giae005)
Supplement: giae005_Reviewer_1_Report_Original_Submission — Cheng Zhao -- 11/18/2023 Reviewed [file giae005_reviewer_1_report_original_submission.pdf]

Reviewer Report

Title: Data processing solutions to render metabolomics more quantitative: case studies in food and clinical metabolomics using Metabox 2.0

Version: Original Submission **Date:** 11/18/2023

Reviewer name: Cheng Zhao

Reviewer Comments to Author:

The manuscript titled "Metabox 2.0: The data processing solution that renders metabolomics more quantitative" provides a comprehensive evaluation of data processing methods in the context of metabolomics, with a focus on achieving results closely aligned with absolute concentrations. The authors address a crucial aspect in metabolomics research, particularly in distinguishing appropriate processing procedures for a given experimental setup.

While the manuscript provides a comprehensive analysis of the impact of different data processing methods on semi-quantified metabolites, some critical questions need to be addressed before publication to improve the clarity of the method and to prove the robustness of the method.

1. While PCA plots are informative, the interpretation could be enhanced by explicitly stating the biological relevance of observed separations or clustering, such as enrichment analysis. A more detailed discussion on the biological implications of the observed patterns in the PCA plots would provide additional context.
2. While the study effectively compares various DP methods, a critical examination of the performances, limitations, and assumptions of each method could further inform the readers, in the form of tables or figures. Discussing the potential biases introduced by certain methods or scenarios where specific methods might not be suitable would contribute to a more balanced evaluation.
3. The decision to exclude major metabolites is justified, but a more extensive discussion on the potential consequences and limitations of this choice would add depth. Addressing how excluding major metabolites may impact the overall conclusions or generalizability of the findings is essential. In another word, how robustness the method would be by only comparing quantified and semi-quantified FAs?
4. The study focuses on the semi-quantitative analysis and its alignment with quantitative data. However, a critical discussion on the inherent limitations and potential biases introduced by semi-quantitative methods compared to absolute quantification would be valuable.
5. The study could benefit from a sensitivity analysis, exploring how the results might vary under different conditions or datasets. Examining the robustness of conclusions by introducing existing pairs of quantified and semi-quantified dataset.
6. English need to be significantly improved.

In summary, the study's approach to evaluating DP methods with respect to quantitative data serves as a novel and valuable contribution to the field. However, the generalizability and the robustness of Metabox 2.0 need to be answered before publication.

Methods

Are the methods appropriate to the aims of the study, are they well described, and are necessary controls included? Choose an item.

Conclusions

Are the conclusions adequately supported by the data shown? Choose an item.

Reporting Standards

Does the manuscript adhere to the journal's guidelines on minimum standards of reporting? Choose an item.

Choose an item.

Statistics

Are you able to assess all statistics in the manuscript, including the appropriateness of statistical tests used? Choose an item.

Quality of Written English

Please indicate the quality of language in the manuscript: Choose an item.

Declaration of Competing Interests

Please complete a declaration of competing interests, considering the following questions:

- Have you in the past five years received reimbursements, fees, funding, or salary from an organisation that may in any way gain or lose financially from the publication of this manuscript, either now or in the future?
- Do you hold any stocks or shares in an organisation that may in any way gain or lose financially from the publication of this manuscript, either now or in the future?
- Do you hold or are you currently applying for any patents relating to the content of the manuscript?
- Have you received reimbursements, fees, funding, or salary from an organization that holds or has applied for patents relating to the content of the manuscript?
- Do you have any other financial competing interests?
- Do you have any non-financial competing interests in relation to this paper?

If you can answer no to all of the above, write 'I declare that I have no competing interests' below. If your reply is yes to any, please give details below.

I declare that I have no competing interests

I agree to the open peer review policy of the journal. I understand that my name will be included on my report to the authors and, if the manuscript is accepted for publication, my named report including any attachments I upload will be posted on the website along with the authors' responses. I agree for my report to be made available under an Open Access Creative Commons CC-BY license (http://creativecommons.org/licenses/by/4.0/). I understand that any comments which I do not wish to be included in my named report can be included as confidential comments to the editors, which will not be published.

Choose an item.

To further support our reviewers, we have joined with Publons, where you can gain additional credit to further highlight your hard work (see: https://publons.com/journal/530/gigascience). On publication of this paper, your review will be automatically added to Publons, you can then choose whether or not to claim your Publons credit. I understand this statement.

Yes Choose an item.
